# Elevated free fatty acid uptake via CD36 promotes epithelial-mesenchymal transition in hepatocellular carcinoma

**DOI:** 10.1038/srep14752

**Published:** 2015-10-01

**Authors:** Aritro Nath, Irene Li, Lewis R. Roberts, Christina Chan

**Affiliations:** 1 Genetics Program, Michigan State University, 567 Wilson Road, Rm 2240E, East Lansing, Michigan 48824, USA; 2 Department of Microbiology and Molecular Genetics, Michigan State University, 567 Wilson Road, Rm 2215, East Lansing, Michigan 48824, USA; 3 Division of Gastroenterology and Hepatology, Mayo Clinic College of Medicine, 200 First St. SW, Rochester, Minnesota 55905, USA; 4 Department of Chemical Engineering and Materials Science, Michigan State University, 428 South Shaw Lane, Rm 2527, East Lansing, Michigan 48824, USA

## Abstract

Hepatocellular carcinoma (HCC) is the second-leading cause of cancer-related death worldwide, and the factors influencing HCC progression are poorly understood. Here we reveal that HCC progression via induction of epithelial-mesenchymal transition (EMT) is closely associated with the expression of CD36/fatty acid translocase and elevated free fatty acid (FFA) levels. Although obesity is manifested as elevated FFA levels, the degree of EMT was not associated with the body mass index of the patients, highlighting the specific roles of CD36 and FFA uptake. Treatment of human liver cancer cell lines with FFAs exacerbated the EMT phenotype, whereas chemical inhibition of CD36 mitigated these effects. Furthermore, the Wnt and TGF-β signaling pathways were activated upon FFA treatment, potentially acting as upstream activators of the EMT program. These results provide the first direct evidence associating CD36 and elevated FFAs with HCC progression.

Primary liver cancer or hepatocellular carcinoma (HCC) is the second most common cause of cancer related deaths worldwide^1^. The incidence rates of HCC have more than tripled in the United States over the past four decades with a steadily increasing mortality rate^2^,^3^. Surgical resection and liver transplantation are potentially curative treatment modalities available for HCC detected at early stages, however, most cases are diagnosed at an advanced stage at which radiotherapy and chemotherapy are either not feasible or are ineffective^4^. Advanced HCC tumors demonstrate high proclivity towards vascular invasion resulting in intrahepatic metastases, which are strongly associated with high post-resection recurrence rates and overall poor prognosis^5^,^6^. Therefore, understanding the mechanisms of HCC progression is important for developing new therapeutic approaches. Evidence from epidemiological studies suggested a link between obesity, manifested in the form of elevated fatty acids, and HCC tumorigenesis and increased mortality^7^,^8^,^9^. Surprisingly, the influences of obesity and elevated fatty acid levels have not been evaluated with respect to the molecular pathogenesis of invasive HCC.

Cancer cells frequently exhibit alterations in fatty acid metabolism to sustain growth and proliferation, fulfill energy requirements and provide metabolites for anabolic processes^10^. Evidence shows that fatty acids are actively transported across cell membrane by specialized proteins instead of by passive diffusion^11^. In the liver, the major proteins involved in fatty acid transport and trafficking included the fatty acid transport proteins, FATP2 (SLC27A2) and FATP5 (SLC27A5) and the fatty acid binding proteins (FABP1, FABP4, and FABP5). The CD36 or fatty acid translocase protein mediates the uptake of fatty acids in a variety of cell types but is expressed at very low levels in normal liver cells, however, its expression is increased in hepatocytes from rodent models of diet-induced obesity, which also correlated with elevated fatty acid uptake^12^,^13^. Thus, alterations in CD36 expression could be involved in enhancing the uptake of FFA into the livers of obese HCC patients.

In a previous study exploring the lipotoxic pathways activated by saturated FFAs, we reported a synergistic association between abrogation of insulin signaling and loss of desmoplakin protein^14^, an obligate component of the desmosomal cell adhesion complex. As desmosomes are lost during epithelial-mesenchymal transition (EMT)^15^, our study suggested a possible role of FFAs in this phenomenon. EMT can be described as a set of well-orchestrated changes, driven by the expression of key transcription factors including *SNAIL*, *ZEB* and *TWIST*, which allow epithelial cells to acquire a mesenchymal phenotype, resulting in enhanced migration, invasiveness, and resistance to apoptosis, thus facilitating progression to a metastatic phenotype^16^,^17^. Several clinical reports described enhanced expression of EMT-genes in advanced stage HCC, correlating with poor prognosis, increased rates of metastases and elevated mortality rates^18^,^19^,^20^. Based on our findings, combined with the epidemiological observations, we hypothesized that elevated FFAs may play a role beyond lipotoxicity or activation of pro-inflammatory pathways, and promote the induction of EMT in HCC.

Here we investigated the association between obesity and elevated uptake of FFAs with the induction of EMT program in human HCC. We first probed the association using mRNA expression data derived from the TCGA liver cancer dataset and then confirmed the observations in human HCC tumor samples. Our data suggests that elevated expression of fatty acid uptake proteins including CD36, and not BMI, was associated with EMT progression. We further established that the saturated FFA palmitate induced EMT in HCC cells by activating the Wnt/β-catenin and TGF-β signaling pathways. Additionally, inhibition of CD36 resulted in reduced migration in liver cancer cells, confirming a critical role of the fatty acid uptake protein in the progression of the EMT program.

## Results

### CD36 expression, and not BMI, is associated with degree of EMT

**Figure 1:**
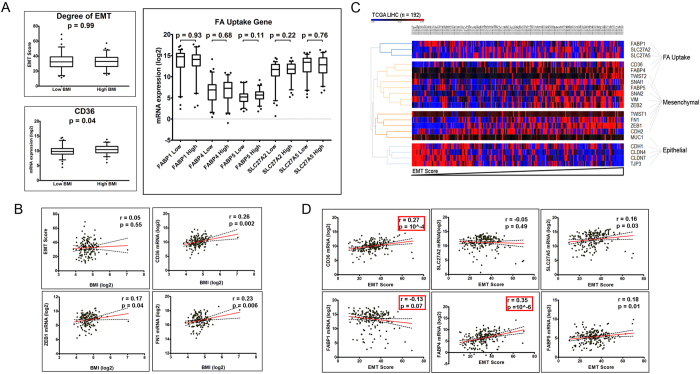
Fatty acid uptake and EMT markers in TCGA liver cancer dataset. (**A**) 158 samples from the TCGA LIHC database were analyzed to determine the effect of patient’s BMI on EMT and FA uptake gene expression. Box plot showing comparison of EMT scores, mRNA expression levels of hepatic FA uptake genes and *CD36* in groups of patients with BMI < 25 (Low BMI, n = 75) or ≥25 (High BMI, n = 83) P-values indicate significance levels from two-tailed Student’s T-test. (**B**) Scatter plots showing correlation between BMI (X-axis) and EMT score, *CD36*, *ZEB1* or *FN1* (Y-axis). The solid red line indicates linear fit, with 95% confidence intervals indicated by dotted black lines. r indicates Pearson’s correlation coefficient and p-values indicate significance of correlation (two-tailed), with the red boxes marking significant associations from the multivariate regression analysis. (**C**) Heatmap showing relative expression levels of hepatic fatty acid uptake genes and EMT genes in the TCGA liver cancer dataset. Gene clusters (vertical axis) were obtained by hierarchical clustering and samples (horizontal axis) were ordered according to the EMT score. (**D**) Scatter plot showing association between the fatty acid uptake genes (Y-axis) and EMT score (X-axis) drawn from 158 patient samples in the TCGA dataset. The solid red line indicates linear fit, with 95% confidence intervals indicated by dotted black lines. r indicates Pearson’s correlation coefficient and p-values indicate significance of correlation (two-tailed), with the red boxes marking significant associations from the multivariate regression analysis (n = 158). Clinical data for each patient is available in Supplementary Table 1 (Bonferroni adjusted p-value cut-off for the analysis = 0.0016).

We investigated the association between patient BMI, expression levels of hepatic fatty acid uptake proteins and EMT progression in the TCGA liver cancer dataset. The TCGA samples were grouped according to the patient’s BMI and an EMT score was assigned to each sample to assess the degree of EMT (Supplementary Table 1). Based on stratification of patients on BMI (low BMI < 25, high BMI ≥ 30), we observed that neither the EMT scores nor the expression levels of FA uptake genes were significantly different between the two BMI groups (Fig. 1A, Supplementary Table 2). Similarly, the pair-wise correlations between BMI and EMT score and FA uptake genes were not statistically significant (Fig. 1B, Supplementary Table 2). We further performed a multivariate regression analysis to verify the association between BMI, EMT score and FA uptake genes controlling for age at initial diagnosis, sex and pathological staging of the patients, and confirmed that the associations with BMI were not significant (Table 1). Next, we clustered the genes involved in FA uptake, epithelial and mesenchymal genes in the TCGA dataset with samples ordered according to EMT score (Fig. 1C). As expected, the epithelial and mesenchymal genes clustered with other members of their respective groups. Intriguingly, the FA uptake genes clustered closer to the mesenchymal genes than the epithelial genes. Therefore, we next investigated the association between EMT score and the expression levels of the FA uptake genes. We found that the pair-wise correlations between EMT scores with *CD36* (p = 10^−4^) and *FABP4* (p = 10^−6^) were statistically significant (Fig. 1D, Supplementary Table 2). Further, multivariate regression analysis controlling for age, sex and staging confirmed the significance of association between EMT score with *CD36* (p = 0.002)*, FABP1* (p = 0.005) and *FABP4* (p = 1.8 × 10^−6^) (Table 1).

These results suggest that while BMI itself did not have an influence, the expression of FA uptake genes was strongly associated with the degree of EMT in HCC patients. Amongst the significant FA uptake genes, *FABP*s are constitutively expressed in liver cells and are involved in intracellular trafficking of FFAs, whereas *CD36* is not expressed in normal hepatocytes and is specifically involved in transporting exogenous FFAs across the cell membrane. Given its lack of expression in normal hepatocytes and significant association with EMT scores, *CD36* emerged as an ideal candidate for subsequent studies.

**Figure 2:**
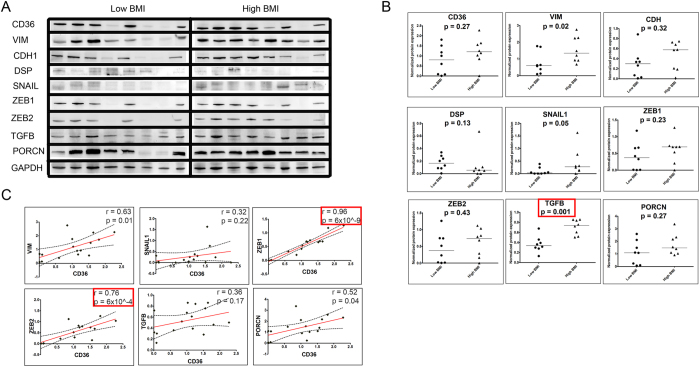
CD36 and EMT marker expression in human HCC tumors. (**A**) Western blots showing expression levels of CD36 and various EMT markers in tumor samples grouped according to BMI. High BMI group (n = 8) represents tumor samples obtained from individuals with BMI ≥ 30 and low BMI group (n = 8) indicates individuals with BMI ≤ 25. Equal amounts of sample (40 μg) were loaded in each well, run under identical conditions and blotted with specific primary antibodies against the indicated proteins. For clarity, blots have been cropped to show relevant bands (full length blots are presented in Supplementary Fig. 3) (**B**). Expression levels of proteins were determined by quantifying the background-subtracted band intensities normalized to GAPDH. Each dot represents relative expression levels of individual samples, with the horizontal bar indicating the median of the distribution. P-values indicate significance of difference in means between low BMI and high BMI groups determined by Mann-Whitney U test, with red boxes marking significant associations from multivariate regression analysis. (**C**) Scatter plot showing association between CD36 (X-axis) and EMT marker (Y-axis) expression. The solid lines indicate linear fit and the dotted lines indicate 95% confidence intervals. r indicates Pearson’s correlation coefficient and p-values indicate significance of correlation (two-tailed), with red boxes marking significant associations from multivariate regression analysis (n = 16). Specific clinical data for each patient is available in Supplementary Table 3.

To verify the association between FA uptake gene expression and EMT observed in TCGA data, we further investigated the protein expression of CD36 and EMT genes in the human HCC tumor samples. The clinical samples were divided into two groups according to the patient’s BMI information. Total cellular proteins were resolved and immunoblotted to detect the expression levels of various EMT markers (Fig. 2A). Vimentin (VIM), a mesenchymal intermediate filament network protein, was measured along with E-cadherin (CDH) and desmoplakin (DSP), cell-adhesion molecules that served as indicators of epithelial characteristics. Expression levels of the mesenchymal marker VIM (p = 0.02) were significantly higher in high BMI tumor samples, but levels of epithelial markers CDH (p = 0.32) or DSP (p = 0.13) were not significantly altered (Fig. 2A,B). Next, we measured the expression levels of ZEB1, ZEB2 and SNAIL1 as transcriptional activation markers of EMT. While the expression levels of SNAIL1 (p = 0.05) were significantly higher in high BMI tumor samples, ZEB1 (p = 0.23) and ZEB2 (p = 0.43) did not differ significantly (Fig. 2A,B). In addition to the EMT markers, we further measured expression levels of TGFB and PORCN as respective indicators of the activation of TGF-β and Wnt/β-catenin signaling pathways. In the high BMI patient group, the expression levels of TGFB (p = 0.001) were significantly higher, but PORCN (p = 0.27) levels did not change (Fig. 2A,B). We next used a multivariate linear regression model to test the association between BMI and the expression levels of the protein markers controlling for sex and tumor grade. Interestingly, this analysis revealed that except TGFB (p=0.004), none of the EMT markers tested in this study were significantly associated with BMI in the human protein samples (Table 2).

Next, we compared expression levels of the EMT markers with CD36 expression across all the samples, irrespective of BMI. As shown in Fig. 2C, the expression of VIM (p = 0.01), SNAIL1 (p = 0.22), ZEB1 (p = 6 × 10^−9^) and ZEB2 (p = 6 × 10^−4^) were positively correlated with CD36 expression. Additionally, the expression levels of TGFB (p = 0.17) and PORCN (p = 0.04) also showed positive correlation with CD36 expression. We next performed the multivariate regression analysis controlling for sex and tumor grade and found that both ZEB1 (p = 2.2 × 10^−7^) and ZEB2 (p = 0.006) were significantly associated with the expression levels of CD36 in the human protein samples (Table 2). While BMI of the patients did not influence the expression of EMT markers, the significant association of CD36 with ZEB1 and ZEB2 expression corroborates the observations made in the TCGA analysis - that elevated CD36 expression may be indicative of the activation of EMT program. Additionally, the expression levels of TGFB and PORCN also showed positive correlation with CD36 expression, suggesting that the TGF-β and Wnt/β-catenin signaling pathways may be the drivers of EMT program activation in the tumor samples with elevated CD36 levels. These results indicate that the elevated levels of CD36 and the consequently elevated uptake of FFAs may be involved in HCC progression via EMT.

### FFA treatment enhances migration and invasion

Having found an association between the fatty acid uptake protein CD36 and EMT regulators, we sought to study the effects of fatty acids themselves on the metastatic behavior of liver cancer cells. We hypothesized that elevated FFAs enhance EMT rates in liver cancer cells. To test this, we first evaluated the rates of migration in human liver cancer cell lines treated with various FFAs. Human HepG2 cells were cultured in media containing carrier control (BSA), the saturated FFA palmitate (PA), monounsaturated FFA oleate (OL), n-6 polyunsaturated FFA linoleic acid (LA), the combinations of PA + LA, or the combination of OL + LA (Supplementary Fig. 1A,B). As shown in Supplementary Fig. 1A,C, the percentage wound closure in BSA was not significant (Day 7 p = 0.39, Day 10 p = 0.16). In comparison, cells treated with PA and OL exhibited significant wound closure by day 7 (Day7, Day10 p < 0.01). However, the cells maintained in polyunsaturated LA also did not show a significant reduction in open-wound area (Day 7 p = 0.63) (Supplementary Fig. 1B,D). We further evaluated the behavior of cells maintained in media containing a combination of different FFAs observing that cells co-treated with PA and LA still showed significant migration (Day 7 p = 0.02) but this effect was not seen in cells co-treated with OL and LA (Day 7 p = 0.16) (Supplementary Fig. 1B,D), suggesting that while saturated FFAs significantly enhanced migration rates, polyunsaturated FFAs did not enhance migration in liver cancer cells.

**Figure 3:**
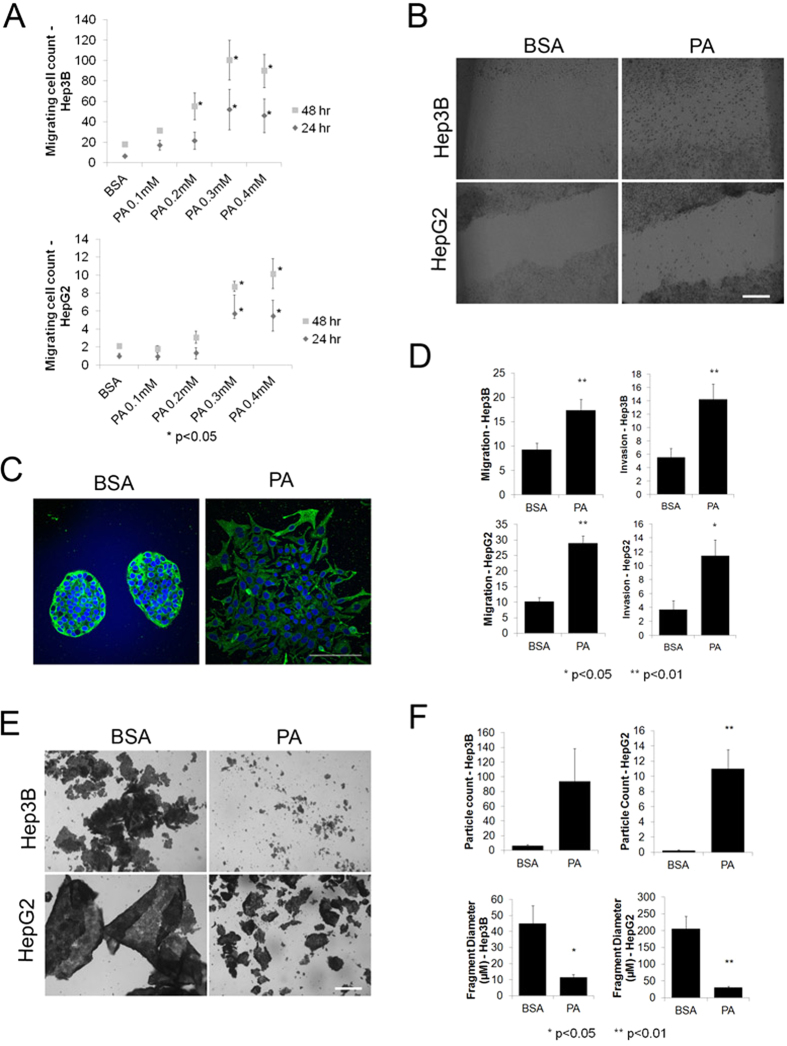
PA mediated EMT induction. (**A**) Scratch-wound cell migration assays with HepG2 and Hep3B cells treated with different concentrations of PA (0.1 mM to 0.4 mM) and BSA (control). The plot indicates average number of migrating cells in the wound area at 24 and 48 hours following 5-day treatment normalized to 0 hour time point (n = 3). P-values indicate the significance levels compared to BSA at a given time point determined by Tukey’s test following one-way ANOVA. (**B**) Phase contrast microscopy images of HepG2 and Hep3B cells treated with BSA or PA (0.3 mM) at 48 hours (40× magnification, scale bar = 50 μm). (**C**) Confocal images of HepG2 demonstrating changes in morphology upon PA treatment. Keratin 18 and nuclear staining are indicated by green and blue fluorescence respectively. (40× magnification, scale bar = 100 μm). (**D**) Modified Boyden’s chamber assays with HepG2 and Hep3B cells. The bar graph indicates the average number of migrating or invading cells (±SEM, Standard Error of the Mean) counted in 5 fields of view per insert (n = 3). P-values indicate significance levels determined by Student’s t-test (two-tailed). (**E**) Bright field images at 5× (scale bar = 400 μm) magnification showing fragmentation in monolayers of BSA or PA treated Hep3B or HepG2 cells following dispase treatment and application of mechanical stress. (**F**) Particle analysis following dispase assay. Bar graphs showing average particle count and fragment diameter (±SEM) determined from 5 independent images panels obtained after dispase treatment and applying mechanical stress to BSA or PA treated HepG2 and Hep3B cells. P-values indicate significance levels determined by Student’s t-test.

Given the strongest effect of PA amongst the different FFAs on cell migration, we further determined the minimum effective dose of PA required to induce migration of liver cancer cells. The subsequent experiments were performed over a shorter time frame (24–48 hours) after pre-treating the cells for 5 days. We evaluated the effects of varying PA concentrations on HepG2 and Hep3B cell migration and observed significantly higher migration with 0.3mM or 0.4mM PA treatment (24/48 hours p < 0.05) (Fig. 3A,B). Thus, the minimum effective dose of PA (0.3 mM) capable of inducing migration was similar to the PA levels found in HCC patients and therefore was used for all subsequent experiments.

As expected in cells undergoing EMT, we noticed a distinct change in the morphology of PA treated HepG2 cells. The cells were immunostained for the cytoskeletal protein keratin 18 following 5 days of treatment with BSA or PA. Interestingly, while all BSA treated HepG2 cells appeared as round or polygonal shaped, some colonies within PA treated cells exhibited an elongated, spindle-shape, characteristic of mesenchymal cells, along with reduced keratin 18 levels (Fig. 3C). This observation further provided evidence that PA treatment may be inducing EMT in the liver cancer cells.

The effects of palmitate on the migration and invasiveness of HepG2 and Hep3B cells were confirmed using the modified Boyden’s chamber assay. These experiments were performed by seeding 5-day treated PA or BSA cells in cell culture inserts in the absence of serum. After 24 hours, both HepG2 and Hep3B cells treated with PA exhibited enhanced migration and invasion compared to BSA treated controls (Fig. 3D).

EMT induction also decreases cell adhesion. To test if PA treatment resulted in loss of liver cancer cell adhesion, we performed a dispase-based cell dissociation assay. After the 5-day treatment with PA or BSA, cultured monolayers were released using the dispase enzyme and subjected to mechanical stress. We found that applying mechanical stress on PA treated HepG2 and Hep3B cells resulted in fragmentation of the monolayer into a large number of smaller-sized particles. In comparison, control cells resulted in fewer, larger-sized particles or sheets (Fig. 3E,F). Thus, we inferred that PA treatment promoted loss of cell adhesion in both HepG2 and Hep3B cells, thereby destabilizing the monolayer.

### Cytotoxicity vs. EMT in FFA treated cells

**Figure 4:**
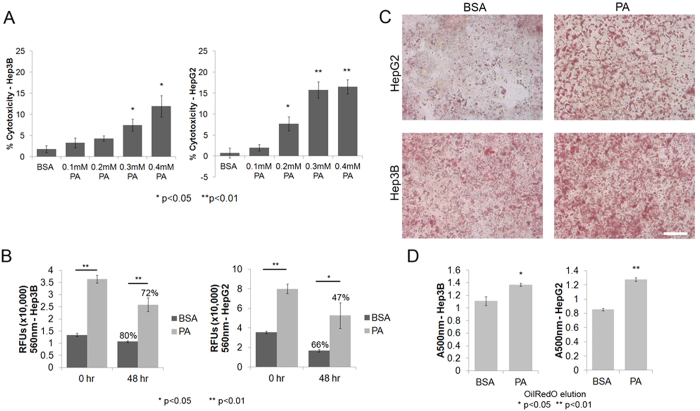
Cytotoxicity vs. metabolic activity. (**A**) Bar graph showing average cytotoxicity (±SEM) determined using LDH assay in HepG2 or Hep3B cells following 5 day (with serum) and 2 day (without serum) treatment with BSA or PA at different concentrations (n = 3). P-values indicate significance levels comparing average LDH release in PA treated cells with BSA treated cells determined by Student’s t-test (two tailed). (**B**) Bar graph showing the relative metabolic activity represented by average fluorescence (±SEM) from reduced AlamarBlue reagent (resazurin) in HepG2 or Hep3B cells following 5 day treatment (with serum) and 2 day (without serum) with BSA or PA (n = 3). P-values indicate significance levels determined by Student’s t-test (two tailed). Numbers above bars indicate the percentage of proliferating cells at 48 hours compared to 0 hours. (**C**) Neutral lipids and fatty acids in HepG2 or Hep3B cells were stained with Oil Red O following 5 day (with serum) and 2 day (without serum) treatment with BSA or PA. Bright-field microscopy images were recorded at 40× magnification (scale bar = 50 μm). (**D**) Relative levels of lipids and fatty acids in BSA and PA treated cells were quantified by eluting the Oil Red O dye and measuring relative absorbance at 500 nm. Bar graph shows average absorbance (±SEM) in BSA and PA treated cells. P-values indicate significance levels comparing average absorbance in BSA or PA treated cells determined by Student’s t-test (two tailed).

A number of previous studies have established that PA is in fact cytotoxic to liver cancer cells, and elevated levels may cause lipotoxicity. Indeed, when HepG2 and Hep3B cells were treated with PA at different concentrations, we noticed significant cytotoxicity at the concentrations corresponding to enhanced migration in cell lines (Fig. 4A). Note this experiment was performed after the 48-hour serum-free treatment with PA or BSA and serum-starvation can also contribute to cytotoxicity. Next, we assessed the metabolic rates in the PA and BSA treated cells before and after serum starvation. While the metabolic activity rates were reduced in both serum-starved PA (HepG2 66%, Hep3B 71%) and BSA (HepG2 47%, Hep3B 80%) treated cells after 48 hours, the basal metabolic rates were significantly higher in PA treated cells when compared to BSA at both 0 (HepG2 2.2 fold, Hep3B 2.7 fold) and 48 hours (HepG2 3.1 fold, Hep3B 2.4 fold) (Fig. 4B). Thus, despite lipotoxicity, the PA treated cells were overall more metabolically active than the BSA treated cells. We further measured metabolic activity by staining for neutral lipids and fatty acids using Oil Red O dye. As shown in Fig. 4C, both PA-treated HepG2 and Hep3B cells show enhanced staining with Oil Red O after 48 hours of serum-starvation compared to BSA treated cells. Additionally, we quantified the relative levels of the dye eluted from stained cells and found significantly higher levels in PA treated cells (Hep3B p<0.05, HepG2 p < 0.01) (Fig. 4D).

**Figure 5:**
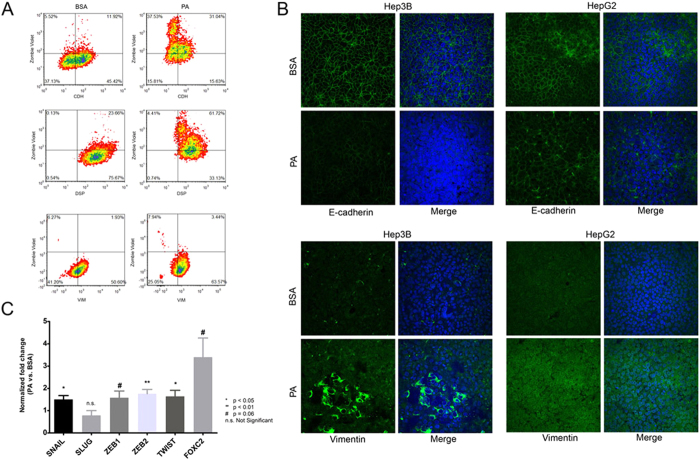
Population effect of PA on EMT marker expression. (**A**) Density plots showing distribution of cells co-stained with zombie violet (Y-axis) and either CDH, DSP or VIM (X-axis) in HepG2 cells treated with BSA or PA (0.3 mM). The bars represent staining thresholds for zombie violet (horizontal bar) or the protein markers (vertical bar). Lower left quadrant represents background staining, upper left quadrant represents cells stained with only zombie violet, upper right quadrant represent cells co-stained with zombie violet and protein marker and lower right quadrant represent cells stained with protein marker only. The numbers in the plot represent the percentage of total cells within that quadrant. (**B**) Confocal images showing specific regions with altered expression levels of CDH and VIM in HepG2 and Hep3B cells. Blue indicates nuclear staining and green indicates CDH or VIM staining. Images were recorded at 40× with identical exposure and PMT settings maintained between BSA and PA treated cells for a given marker (scale bar = 100 μm). C Bar graph showing fold-change in mRNA expression levels of various EMT transcription factors determined by qRT-PCR in PA (0.3 mM) treated HepG2 cells compared to corresponding expression levels in BSA treated cells (*GAPDH* normalized). P-values indicate significance levels compared to BSA determined by Student’s t-test (two tailed), n = 3.

From these experiments, we hypothesized that while PA induced cell death in a proportion of the liver cancer cells, a distinct surviving population of cells remained metabolically active and acquired the ability to undergo EMT. To further confirm this point, we measured the changes in expression levels of various EMT markers in the PA treated liver cancer cells. We used flow cytometry to analyze the population distribution of cytotoxicity and EMT markers (Fig. 5A, Supplementary Fig. 5). We stained PA or BSA treated HepG2 cells with a cell death indicator dye (Zombie Violet) followed by formaldehyde fixation. The fixed cells were then permeabilized and immunophenotyped for CDH1, DSP (epithelial markers), and VIM (mesenchymal marker). After background subtraction from unstained cell populations, we analyzed the percentage of cells that were positively stained for each marker. As expected, we found that upon PA treatment, a larger percentage of cells stained positive for Zombie Violet as compared to BSA treatment (upper left quadrant, Fig. 5A). Moreover, another separate population of cells stained positive for the epithelial and mesenchymal markers (lower right quadrant Fig. 5A). The percentage of cells that stained positive for CDH in BSA (45.42%) was significantly higher than PA (15.63%) (χ^2 ^= 21.43, df = 1, p < 0.001). Similarly, a higher percentage of DSP staining was observed in BSA (75.67%) compared to PA (33.13%) (χ^2 ^= 26.7, df = 1, p < 0.001). In contrast, a larger number of PA cells (63.57%) showed positive staining for VIM, compared to BSA cells (50.6%) (χ^2 ^= 3.5, df = 1, p = 0.03).

The expression of the EMT markers was further confirmed by observing the immunostaining using confocal fluorescence microscopy. HepG2 and Hep3B cells were fixed, permeabilized and stained to detect CDH and VIM expression (Fig. 5B). A distinct population of PA treated cells showed reduced CDH expression and increased VIM expression in both HepG2 and Hep3B cells. Within the population of PA treated Hep3B cells, a few cells show very high expression levels of VIM, further corroborating presence of distinct cell populations.

The mRNA expression levels of the EMT transcription factors *SNAIL1* (p < 0.05), *ZEB2* (p<0.01) and *TWIST1* (p < 0.05) were significantly higher in PA treated HepG2 cells and similarly *ZEB1* and *FOXC2* were expressed at higher levels in the PA treated cells (p = 0.06) (Fig. 5C). The lower fold-change observed in the qRT-PCR experiment reflects population average across all cells, including cytotoxic and EMT cells, within a treatment condition.

### FFA treatment activates Wnt and TGF-β signaling

**Figure 6:**
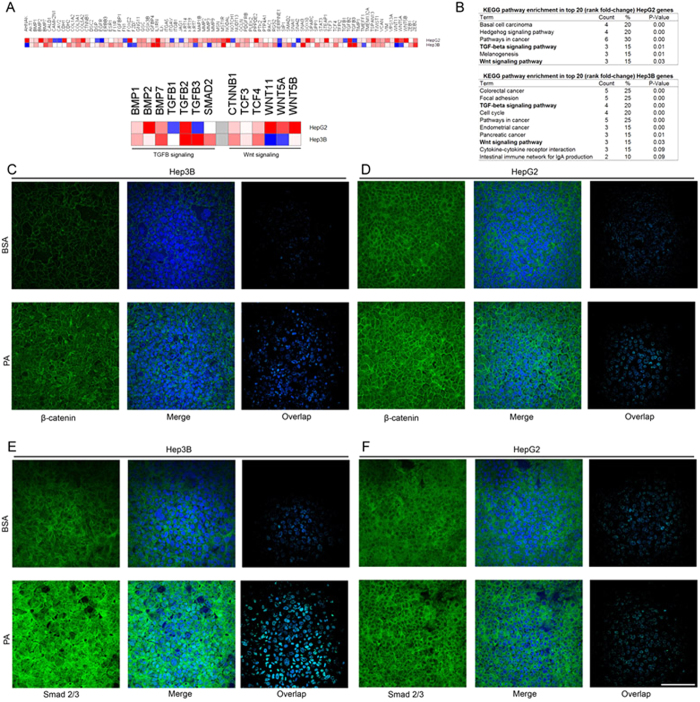
EMT pathways induced by PA. (**A**) Heatmap showing relative fold-change in mRNA expression levels of various EMT-related genes in PA (0.3 mM) vs. BSA treated cells measured by EMTarray. The enlarged heatmap shows fold change in expression levels of the components of TGFB- and Wnt-signaling pathways included in the array. (**B**) The top 20 genes ranked according by fold-change in the array were enriched for KEGG pathways. The table shows the term (KEGG pathway) enriched, number and percentage of genes in the input set belonging to the pathway and significance levels of enrichment. (**C**–**F**) Confocal images showing expression of β-catenin or SMAD2/3 (green) and nuclei (blue) in HepG2 and Hep3B cells treated with BSA or PA (0.3 mM). The overlap panel shows pixels where both green and blue fluorescence co-localize, therefore indicating cells displaying translocation of β-catenin or SMAD2/3 transcription factors to the nucleus. Images were recorded at 40× with identical exposure and PMT settings maintained between BSA and PA treated cells for a given marker (scale bar = 100 μm).

With evidence to support that PA treatment activated the EMT program, we next sought to understand the driving mechanism behind EMT activation in the PA treated cells. We used the mRNA samples from BSA and PA treated HepG2 and Hep3B cells and profiled the expression levels of genes that were representative of known EMT-inducing signaling pathways using a PCR-based array (Fig. 6A). In this study, we ranked the genes obtained from the array according to the PA vs. BSA fold-change and analyzed the enrichment of KEGG-pathways in the top 20 genes (Fig. 6B, Supplementary Table 4). While the hedgehog signaling pathway was enriched in the HepG2 cells, we did not find a significant increase in the sonic hedgehog ligand (*SHH)* and its downstream transcriptional activator *GLI1* mRNA levels in PA treated HepG2 cells (Supplementary Fig. 2). We found that the TGF-beta and Wnt signaling pathways were significantly enriched in the set of top 20 genes from both HepG2 and Hep3B cells (Fig. 6B). Interestingly, a distinct set of genes within these pathways were upregulated in the two cell lines (Fig. 6A). Recall that in the human HCC tumor samples in Fig. 2C, we found a positive correlation between CD36 with TGFB (TGF-beta signaling ligand) and PORCN (Wnt signaling mediator). Furthermore, known transcriptional targets of Wnt and TGF signaling pathways including *CALD1, CDH1, CDH2, COL3A1, COL5A2, ESR1, FOXC2, GSK3B, TCF4, TGFB2, WNT5A, and WNT5B* were among the top 20 genes upregulated in PA treated HepG2 or Hep3B cells (Supplementary Table 4). These results provide a point of convergence between the correlations observed in the human HCC studies and the experiments with PA treated liver cancer cells – that TGF-beta and Wnt signaling pathways are activated in both cases and may be the drivers behind activation of EMT program.

The activation of TGF-beta and Wnt signaling pathways promote the subsequent activation of EMT transcription factors, driving the expression of several other anti-apoptotic and proliferative oncogenes. This requires the activation and nuclear translocation of downstream transcriptional effectors of TGF-beta and Wnt signaling pathways. Thus, to assess the activation of the two pathways, we investigated the intracellular localization of SMAD2/3 and β-catenin in BSA and PA treated cells. The overall expression levels of β-catenin were higher in both HepG2 and Hep3B cells after PA treatment (Fig. 6C,D). Moreover, the expression of β-catenin was restricted to the cell membrane and cytoplasm in BSA treated cells, while in PA treated cells we clearly observed its increased localization to the nucleus, suggesting that the Wnt signaling pathway was activated upon PA treatment of both HepG2 and Hep3B cells. The expression levels of SMAD2/3 were higher in PA treated Hep3B cells (Fig. 6E) but did not seem to change in PA treated HepG2 (Fig. 6F) cells, corroborating the data from the array (Fig. 6A). Additionally, SMAD2/3 staining showed considerably higher nuclear localization in PA treated Hep3B cells (Fig. 6E), but this change was not distinct in the PA treated HepG2 cells (Fig. 6F). This indicated differing mechanisms of EMT induction in the two cell lines and a proclivity of the hepatoblastoma-derived HepG2 cells towards activation of the Wnt-signaling pathway.

**Figure 7:**
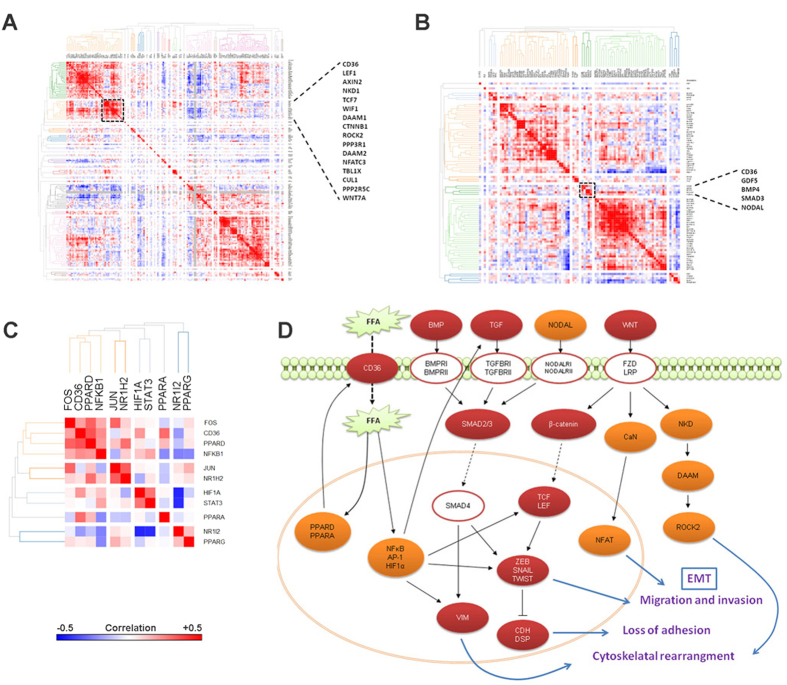
Correlation matrix heatmaps showing the association between mRNA expression z-scores (TCGA) of *CD36* and (**A**) Wnt signaling pathway genes (KEGG) (**B**) TGF beta signaling pathway (KEGG) (**C**). PPARs and transcriptional mediators of inflammatory pathways known to be induced by free fatty acids. Genes are clustered with average linkage hierarchical clustering using 1-Pearson’s correlation as distance metric. Black boxes in (**A**,**B**) show the nearest neighbors of CD36 with strong positive correlation, with enlarged list showing the names of genes in that cluster. (**D**) Overview of the influence of elevated free fatty acid uptake on cell signaling pathways resulting in the induction of EMT program. Red ovals represent genes that were upregulated *in vitro* in PA treated HCC cells; orange ovals represent genes that show positive correlation with *CD36* in the TCGA dataset; white ovals represent genes that did not show a significant correlation with CD36 in the TCGA dataset. Dashed black arrows show transport/translocation, solid black arrows and bar-headed lines indicate positive and negative regulation respectively. Blue arrows indicate consequence of activation of the proteins in context of the EMT program.

We further investigated the mRNA expression profiles of the Wnt (Fig. 7A) and TGF-beta (Fig. 7B) signaling genes in the TCGA dataset in context of *CD36* expression (Supplementary Table 5). The components of these two signaling pathways that share a similar expression pattern as *CD36* (marked clusters in dashed box) reveal possible mechanism by which these pathways may be associated with elevated FFA uptake. The BMP family ligands (*GDF5*, *BMP4*), *NODAL*, and the transcriptional effector (*SMAD3*) of TGF-beta signaling pathway show similar expression pattern as *CD36*. Similarly, the Wnt ligand (*WNT7A)* and the canonical Wnt signaling transcription factors *CTNNB1*, *LEF1* and *TCF7* clustered with *CD36.* Additionally, genes involved in non-canonical Wnt signaling pathways including planar polarity pathway (*DAAM1, ROCK2)* that regulates cytoskeletal rearrangement, and the Wnt/Ca^2+^ pathway (*PPP3R1, NFATC3*) that regulates cell adhesion and migration, also exhibit similar expression patterns as *CD36*. Furthermore, we found that transcriptional targets of TGF-β signaling: *COMP, ROCK2, THBS2, AXIN2, BTRC, CCND2, CSNK1A1, CTBP2, FRAT1, FZD4, NFAT5, NFATC3, NKD2, PPP2R5C, PRKACB, and PRKX,* as well as, targets of WNT/β-catenin signaling: *BMP4, ID2, AXIN2, LEF1, and MMP7*, were significantly associated with *CD36* expression in TCGA HCC dataset (Supplementary Table 5).

Next, we analyzed the expression patterns of transcription factors that are known to be activated by elevated free fatty acids (Fig. 7C). We found that the fatty-acid responsive transcription factors, *PPARA* and *PPARD,* that can transcriptionally upregulate *CD36*^21^, show strong positive correlation with *CD36* (Supplementary Table 5). Furthermore, a number of downstream transcriptional effectors of inflammatory pathways (TNF-α, Jak/Stat, IL1-signaling) activated by free fatty acids, including *NFKB1*, *FOS* and *HIF1A* show similar expression pattern as *CD36*. These transcription factors can upregulate the expression of Wnt and TGF-beta signaling pathway components, and also upregulate expression of EMT transcription factors. Figure 7D provides an outline of the influence of elevated uptake of FFA on the various signaling pathways constructed using the results from our *in vitro* experiments with PA treated HCC cells, and the evidence from TCGA analysis. As shown in the schematic, the uptake of FFA via CD36 is a key event in the induction of EMT program in HCC cells. Therefore, we hypothesized that inhibition of CD36 would result in abrogation of the EMT phenotype.

**Figure 8:**
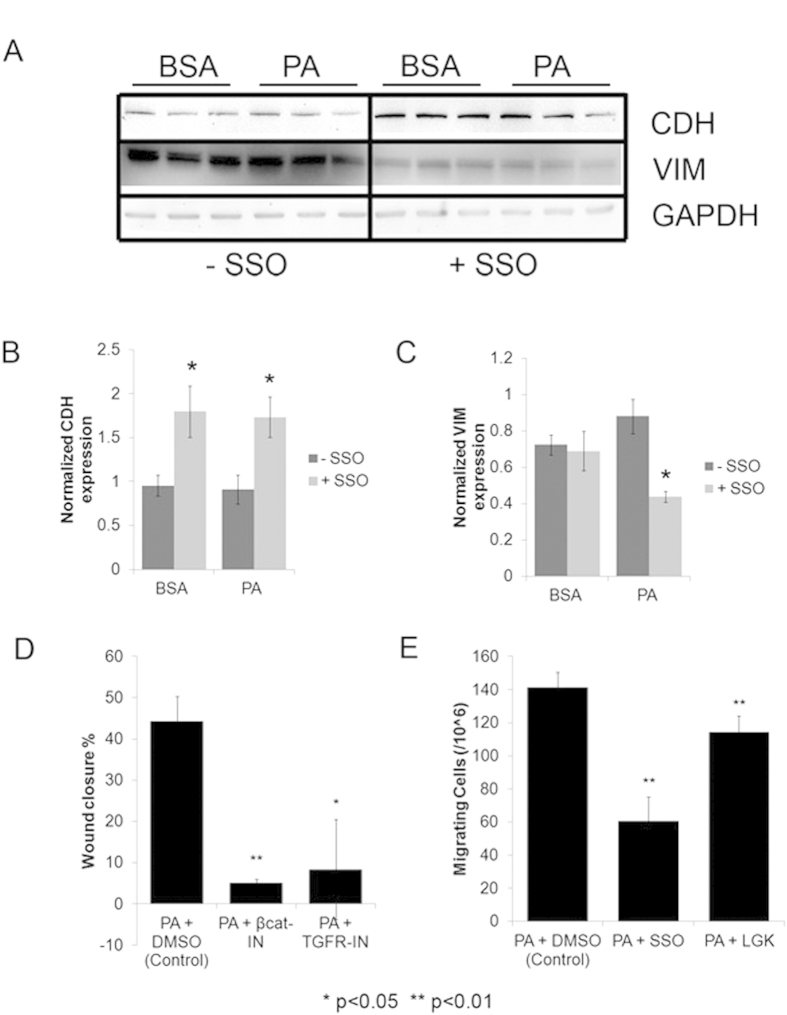
CD36 and TGF-beta/Wnt-signaling mediate PA effects. (**A**) Western blots showing expression of CDH and VIM in HepG2 cells treated with BSA or PA (0.3 mM) for 5 days with serum. Additionally, the cells were co-treated with either 100 μM SSO (CD36 inhibitor) or DMSO (solvent control). Equal amounts of sample (40 μg) were loaded in each well, run under identical conditions and blotted with specific primary antibodies against the indicated proteins. For clarity, blots have been cropped to show relevant bands (full length blots are presented in Supplementary Fig. 4). (**B**–**C**) Bar graphs showing quantification of blots indicating expression levels of CDH and VIM normalized to GAPDH in BSA or PA treated cells (n = 3). P-values indicate significance levels comparing SSO treated (+SSO) BSA or PA treated cells with DMSO control (-SSO) cells determined by Student’s t-test (two tailed). (**D**) HepG2 cells were treated with PA (0.3 mM) and DMSO (control), β-catenin/Tcf inhibitor III (100nM) or TGF-β1 RI kinase inhibitor (50 nM) in presence of serum for 5-days, followed by woundhealing assay in serum-free media over 48 hours. Bar graph indicates average wound closure (wound closure = 100 - % open wound area) normalized to 0 hour time point (n = 3). P-value indicates significance levels comparing the open wound area in inhibitor treated cells with control cells determined by Student’s t-test (two tailed). (**E**) HepG2 cells were treated with PA (0.3 mM) and DMSO (control), CD36 inhibitor SSO (100μM) or porcupine inhibitor LGK974 (100 nM) in presence of serum for 5-days, followed by Boyden’s chamber migration assay over 48  hours in serum-free media, with 10% FBS containing regular medium as chemoattractant. Bar graph indicates the average number of migrating cells (n = 3). P-value indicates significance levels comparing the number of migrating cells with inhibitor treatment against control, determined by Student’s t-test (two tailed).

To verify the critical role of CD36 in mediating the effects of PA on the liver cancer cells, HepG2 cells were cultured in BSA or PA along with Sulfo-N-succinimidyl oleate (SSO) – a chemical that binds irreversibly to the CD36 receptor and inhibits fatty acid uptake^22^. After the 5-day treatment period, we measured the protein expression levels of CDH and VIM (Fig. 8A). Note that the PA treatment expression levels reflect the population average across both cytotoxic and EMT cells. We found that inhibiting CD36 activity with SSO resulted in significant upregulation of CDH in not only PA treated cells, but also in BSA treated cells (Fig. 8B). In other words, the inhibition of CD36 was sufficient to recover the mild-EMT phenotype that was observed in BSA treated cells as well. Additionally, the expression levels of VIM were significantly reduced in PA treated cells when CD36 was chemically inhibited (Fig. 8C).

To assess the role of TGF-beta and Wnt signaling pathways in mediating the activation of EMT program, we measured the effect of inhibiting these pathways in PA treated HepG2 cells. We found that migration rates were significantly reduced in PA treated cells when they were co-treated with a β-catenin/Tcf inhibitor III inhibitor against the Wnt signaling pathway^23^ or TGF-β1 RI kinase inhibitor against the TGF-beta signaling pathway^24^ (Fig. 8D). Additionally, the inhibition of the PORCN enzyme activity with LGK974^25^ also resulted in significant reduction of migration rates (Fig. 8E). Finally, the inhibition of CD36 with SSO reduced migration rates in PA treated cells (Fig. 8E), confirming the importance of CD36 in mediating PA induced EMT in the liver cancer cells. These experiments confirm that the induction of EMT by PA is mediated by CD36 and requires activation of TGF-beta and Wnt signaling pathways.

Finally, we used the TCGA HCC dataset to assess the influence of stratification of tumor samples based on low or high CD36 expression clusters (Supplementary Table 6) and low (<25) or high BMI (≥30) groups (Supplementary Table 7). The analysis revealed that none of the 3395 chemical and genetic perturbation gene sets were enriched in either of low and high BMI groups (p-value < 0.05, FDR < 25%). Additionally, no gene sets were enriched in low CD36 group; however 27 gene sets were enriched in the high CD36 group and 10 out of the 27 significant gene sets are liver cancer signatures, lending further support to the specific role of CD36 in liver cancer independent of BMI.

## Discussion

Cancer cells frequently exhibit alterations in fatty acid metabolism to sustain growth and proliferation, fulfill energy requirements and provide metabolites for anabolic processes^10^. It has been observed that FFA levels are significantly elevated in the plasma of HCC patients^26^,^27^. However, the precise role of elevated FFA levels in tumorigenesis and cancer progression remains controversial. While a number of studies have shown that accumulation of FFAs, especially saturated FFA palmitate, leads to mitochondrial dysfunction and generation of reactive oxidative species (ROS) that contributes to loss of cellular homeostasis and cell death, a phenomenon termed as lipotoxicity^28^ other studies have shown TNF-α and IL-6 cytokine-induced inflammation^29^, aggravated insulin resistance^30^ and enhance endoplasmic reticulum stress^31^ in hepatocytes. Although obesity has been strongly associated with progression and high mortality rates in HCC patients^7^,^32^, the role of FFAs in HCC progression has remained undefined. Our results provide the first direct evidence that elevated FFA uptake via CD36 is associated with EMT induction in HCC. However, we also observed that BMI was not associated with the degree of EMT, highlighting the specific role of FFAs and their cellular uptake in this process. Note that BMI itself may not be an accurate indicator of the level of fatty acids in an individual, as athletic and fit individuals with higher bone density or muscle mass could have high BMI despite low fat levels. Further, BMI at the time of HCC diagnosis may not be fully reflective of the patients BMI during carcinogenesis due to the frequent occurrence of paraneoplastic weight loss once the cancer is established.

While the fundamental role of hepatic FFA pool is to provide energy and metabolites for other anabolic pathways, our results suggest a role of FFAs beyond cellular sustenance. We observed that Wnt and TGF-β signaling pathways were activated by palmitate and there could be several mechanisms involved. Oncogenic transcription factors, including NF-κB, AP-1 and STAT3, activated by inflammatory cytokines like TNF-α and IL-6 could connect the activation of Wnt and TGF-β signaling pathways with elevated FFA^33^. The activation of NF-κB in response to elevated FA levels was shown previously by our laboratory^34^, and may drive EMT via activation of WNT and TGF signaling. For instance, NF-κB was shown to upregulate Wnt signaling transcription factor *LEF1*^35^, and along with AP-1 co-regulated the expression of *WNT10B* ligand^36^. Moreover, previous studies have shown that the influence of unsaturated FFAs on the EMT phenotype in hepatocytes and other cell types was dependent on the mTOR/NF-κB pathway^37^ or PI3K/Akt- NF-κB pathway^38^. In addition, *TGFβ*, *ZEB1* and *ZEB2* expression were shown to be regulated by AP-1^36^. The STAT3 transcription factor was shown to mediate the induction of EMT by regulating *SNAIL*^37^, *ZEB1*^38^, *TWIST*^39^ and the mesenchymal markers *VIM* and *CDH12*^40^. A different mechanism of EMT induction could be the activation of the unfolded protein response or ER-stress pathway by FFA^41^. The involvement of downstream transcription factor of ER-stress, HIF1α, in promoting EMT is well documented in multiple cancers via the activation of Wnt signaling^42^ and the direct regulation of *TWIST*^43^, *TCF3* and *ZEB*^44^. In addition to fatty acid uptake, CD36 also mediates uptake of HDL (high density lipoproteins)^45^ and oxidized LDL (low density lipoproteins)^46^ in hepatocytes. A recent study suggests the uptake of oxidized LDL requires binding of fatty acids to CD36^47^. Elevated oxidized LDL accumulation has been associated with increased ROS accumulation, induction of ER-stress and activation inflammatory pathways in the liver^48^,^49^,^50^, thereby potentially activating the pro-EMT signaling pathways. Thus, the CD36-mediated increase in hepatic FFA accumulation could facilitate the production of EMT-inducing factors.

The phenotypic effects that we observed in our studies may also be explained on the basis of the scavenger-receptor signaling pathway associated with FA binding with CD36. Previously it was shown that inhibition of CD36 activity by SSO inhibited FA uptake^51^,^52^, but could also inhibit CD36-dependent FA signaling^53^,^54^,^55^. CD36 is a multi-functional protein and exhibits a variety of different signaling functions upon binding with different ligands. CD36 crosstalk with TLR2/6 heterodimer results in activation of TLR-dependent cytokine production (NF-κB)^56^. Its interaction with thrombospondin 1 (TSP1) also directly activates an apoptotic response via the kinases p38 and JNK^57^. The complex of oxidized low-density lipoprotein (oxLDL) and CD36 leads to increased activation of focal adhesion kinase 1 (FAK1) and RAC (via VAV1) which ultimately results in changes in cell morphology, such as loss of polarity and actin polymerization^58^. In addition, knockout experiments have identified that mitogen-activated protein kinase (MAPK) and caspase-3 can be activated downstream of CD36 signaling^59^. In the context of EMT, the relationships of these pro-inflammatory pathways with CD36 point to a possibility that FFA may activate EMT via CD36 signaling, thereby explaining in part the strong influence of SSO treatment on the expression of EMT markers and migration rates. The creation of an environment promoting EMT would not only play a role in progression of HCC, but may also provide a conducive environment for liver metastases arising from other organs, such as colorectal cancers. The majority of colorectal cancer patients (>50%) develop liver metastases, and while surgical resection of the liver metastases facilitates long-term survival, only a small fraction of the patients are candidates for resection^60^. Given the need for new targets for both advanced colorectal cancers and HCC, future studies investigating the mechanism of EMT induction, and the role of elevated FFAs and FA uptake proteins are warranted.

## Materials and Methods

### Human HCC tumor samples, TCGA data and EMT score calculation

De-identified, IRB-approved frozen samples were obtained from the “International Registry of Patients with or at Risk for Hepatobiliary Cancers, Including Hepatocellular Carcinoma, Cholangiocarcinoma, and Gallbladder Adenocarcinoma, and those Patients with Normal Risk Factors”. This Human Subjects Research falls under Exemption 4 because the research involves the study of existing data, records, and pathological or diagnostic specimens of de-identified IRB-exempt samples from Dr. Lewis Roberts. The research was approved by the Mayo Clinic Institutional Review Board (IRB) under protocol 14-005015 with waiver of the requirement to obtain informed consent and was carried out in accordance with the approved guidelines. All experimental protocols were approved by the Mayo Clinic IRB. Patient clinical information, including body mass index, was provided by Mayo Clinic. Total cellular proteins were extracted from the tumor samples using T-PER protein extraction reagent (Pierce, 78510) supplemented with protease inhibitor cocktail tablets (Roche, 04693159001) according to the manufacturer’s protocol.

Liver cancer gene expression data (mRNA, normalized RNAseqV2 RSEM) were retrieved from the TCGA database^61^ using the cBioPortal for cancer genomics^62^,^63^. Expression levels were log_2_ transformed and EMT scores were calculated using a previously published metric^64^. EMT score = Sum of mesenchymal gene expression (*CDH2, FN1, SNAI1, SNAI2, TWIST1, TWIST2, VIM, ZEB1, ZEB2*) – Sum of epithelial gene expression (*CDH1, CLDN4, CLDN7, MUC1, TJP3*). Hierarchical clusters and heatmap visualizations were generated using the GENE-E matrix analysis platform (http://www.broadinstitute.org/cancer/software/GENE-E/index.html) with TCGA gene expression data (mRNA, RNAseq z-scores) retrieved using the cBioPortal. Clusters were obtained using the average linkage method with 1-Pearson’s correlation coefficient as the distance metric. Gene set enrichment analyses were performed using GSEA module available from the GenePattern genomics server (http://genepattern.broadinstitute.org/).

### Cell lines, culture medium and treatment

Human liver cancer cell lines (HepG2, Hep3B) were obtained from ATCC, cultured in DMEM (Gibco, 11965), supplemented with 10% FBS (Gibco, 16000) and 1% penicillin/streptomycin (Gibco, 15140), and maintained in a humid, 5% CO_2_ environment at 37 °C.

FFA preparation media was made by dissolving 2% w/v BSA (US Biologicals, A1311) in serum-free DMEM supplemented with 1% penicillin/streptomycin. Palmitate medium was prepared from a high concentration (40 mM) stock solution made by dissolving sodium palmitate (Sigma, P9767) in dH_2_O heated to 70° C. An aliquot from the stock was then added drop wise to 2% BSA medium with constant stirring to achieve the desired final palmitate concentration. Oleate (Sigma, O7501) and linoleic acid (Sigma, L8134) media were prepared by directly dissolving the appropriate amounts in BSA media. FFA media with serum was prepared by adding FBS to a final concentration of 10%.

Chemical inhibitors: 0.5 mg/ml TGF-β RI Kinase Inhibitor (Calbiochem, 616451), 1mg/ml β-Catenin/Tcf Inhibitor III iCRT3 (Calbiochem, 219332), 5mg/ml LGK-974 (Selleck chemicals, S7143) and 25 mg/ml Sulfosuccinimidyl Oleate (SCBT, sc-208404) stocks were prepared in 100% DMSO and diluted to indicated concentrations for *in vitro* experiments.

### Dispase-based dissociation assay

Dispase assay was adapted from previous studies^65^,^66^. Cultured monolayers in 6-well plates were washed twice with ice-cold PBS. 2 ml of Dispase (1 U/ml) enzyme solution (Stemcell technologies, 07923) was added to each well and incubated at 37 °C for 30 min. The released monolayers were then carefully aspirated into a 15 ml centrifuge tube and washed with ice-cold PBS by centrifuging at 1 g for 1 min. The monolayers were then disrupted by applying mechanical stress by pipetting 5× with a 1 ml pipette (tube labels were concealed and pipetted in randomized order to avoid bias). The resulting fragments were stained with crystal violet (0.05% w/v in methanol) and transferred to 6-well plates for recording images with a light microscope. Images were analyzed to calculate fragment number and size distribution using ImageJ software^67^.

### Scratch-wound healing, migration and invasion assays

For scratch-wound healing assays, cells were seeded in 24-well plates with regular media. After a 24-hour acclimatization period, the cells were washed with PBS and cultured for 5 days in FFA or BSA media with serum to encourage EMT induction. Media was refreshed every 48 hours. Following the 5 day incubation, cells were washed with PBS and using the tip of a sterile 10 uL pipette tip, a single scratch was made on the cell surface within each well. Perpendicular marks were made to standardize viewing fields. Cells were then cultured in the corresponding serum-free FFA or BSA media and wound healing was monitored over a 48-hour time course. The serum-free media avoided confounding effects of proliferation. Three standardized bright-field images were recorded for each scratch at the 0, 24, and 48-hour time points. The rates of migration were assessed by either quantifying the percentage of open wound area using T-scratch package^68^ or by counting the number of individual migrating cells in the wound area using automated particle analysis with ImageJ software.

For modified Boyden’s chamber assays, following 5 days culture in FFA or BSA media with serum, cells were collected by trypsinization, neutralized with regular media, washed with PBS by centrifugation at 200 g for 3 min. and counted using a hemocytometer. The cells were then re-suspended in FFA or BSA medium without serum and seeded on BD Biocoat 8μm membrane inserts (BD Biosciences, 354480) with Matrigel coating (for invasion assay) or without Matrigel coating (for migration assay). The inserts were placed in wells containing regular DMEM media containing 10% FBS as chemoattractant. After 24 hours, the inserts were removed, washed with PBS, fixed in methanol and stained with crystal violet (0.05% w/v in methanol). The bottom surfaces of the stained inserts were then observed under a light microscope, and the numbers of stained cells were counted in 5 fields/insert.

### Cytotoxicity/metabolic activity assays and Oil Red O staining

Cytotoxicity and metabolic activity were assessed by microplate assays. Cells were seeded in 96-well plates and treated with PA or BSA with serum over 5 days, followed by serum free treatment. Cytotoxicity was detected using the LDH cytotoxicity detection kit (Roche, 11644793001) following the manufacturer’s protocol. Metabolic activity was determined by AlamarBlue assay (Pierce, 88952) following the manufacturer’s protocol.

For Oil Red O staining, cells treated in 6-well plates were washed with PBS and fixed with 10% formaldehyde at room temperature for 1 hour. Cells were washed 2× with dH_2_O and washed 2× with 60% isopropanol for 5 min. each at room temperature. The cells were then allowed to dry in a fume hood for 30 min. Next, 1 ml of Oil Red O (Sigma, O0625) staining solution (35 mg/ml in 60% isopropanol) was added and incubated for 10 min. at room temperature. Cells were washed 4× with dH_2_O and bright-field images were recorded at 40× magnification. For the elution assay, the cells were allowed to air-dry for 30 min and the dye was eluted by adding 1ml 100% isopropanol and incubating on a shaker for 10 min. The eluted dye was mixed by pipetting and aliquoted in a 96-well plate to measure absorbance at 500 nm with 100% isopropanol as blank.

### qRT-PCR and arrays

Total mRNA for qRT-PCR and PCR array studies was extracted from cells using RNeasy Mini Plus kit (Qiagen, 74134) according to the manufacturer’s protocol. cDNA was synthesized from 1μg of total mRNA using High-capacity cDNA reverse transcript kit (Applied Biosystems, 4368814) following the manufacturer’s protocol. qRT-PCR experiments were performed in a MyiQ thermal cycler (BioRad) with Sybr-Green mastermix (BioRad, 170–8882) and 200 ng of template/reaction using primers (Operon) designed using the Primer3/NCBI Primer-BLAST tool^69^ (Table 3).

The following thermal cycling settings were used: 95 °C for 10 min. followed by 40 cycles at 95 °C for 30 sec., 62 °C for 1 min. and 72 °C for 1 min. The fold-change values were calculated as delta-delta Ct (ddCt) values from a minimum of three independent biological replicates.

The PCR array study was performed with an 84-gene EMT pathway-specific array (SABiosciences, PAHS-090Z) according to the manufacturer’s protocol. The top 20 genes were ranked by fold change magnitude and analyzed for KEGG pathway enrichment using the DAVID bioinformatics tool^70^.

### Western blots

Cells were washed with ice-cold PBS and incubated with ice-cold RIPA buffer supplemented with protease inhibitor cocktail tablets (Roche, 04693159001) for 30 min. on ice with gentle shaking. The extracts were transferred to pre-chilled tubes and centrifuged for 10 min. at 10,000 g to separate cell debris from supernatant with proteins. Total protein levels were quantified with micro BCA assay (Pierce, 23235), reduced in Laemmli buffer (NEB, B7703S) and resolved on 10% SDS-PAGE gels by loading 20–40 ug of protein per well. The resolved proteins were transferred to 0.45 μm nitrocellulose membrane and blocked (5% BSA or milk in TBST) for 1 hour at room temperature. The membranes were incubated with specific primary-antibodies (Table 4) diluted in incubation buffer (2.5% BSA, 0.01% sodium azide in TBST) overnight at 4 °C, followed by incubation with isotype-specific secondary antibodies diluted (1:1000) in TBST for 1 hour at room temperature. For reprobing, the membranes were incubated 2× in stripping buffer (0.2 M glycine, 0.1% SDS, 1% Tween20, pH = 2.2) for 5 min each followed by two washes with PBS and TBST for 5 min. each. The membranes were then blocked and incubated in antibodies as described above. Chemiluminescence was detected with ECL substrate (Pierce, 34080) in a BioRad gel doc. Band intensities were quantified with ImageJ software and normalized to loading control (GAPDH).

### Confocal microscopy

Cells were cultured in glass-bottom 24-well plates (*In Vitro* Scientific) and treated with FFA or BSA medium. After treatment, cells were washed 2× with ice-cold PBS and fixed with 3.7% formaldehyde for 10 min. at 37 °C, washed 2× with PBS, and permeabilized with 0.5% TritonX-100 for 10 min. at room temperature. After 2× washes with PBS, the samples were incubated in blocking buffer (1% BSA in PBS) for 1 hour at 37 °C. Next, the cells were incubated with diluted primary antibodies (CDH 1:500, VIM, 1:500, β-catenin 1:1000, SMAD2/3 1:2000) in incubation buffer (0.5% BSA with 0.01% Sodium Azide in PBS) overnight at 4 °C. After the overnight incubation, samples were washed 2× with PBS and incubated in respective secondary antibodies (AlexaFluor 488) diluted in PBS for 1 hour at 37 °C in the dark. Cells were washed 2× with PBS and incubated in nuclear counter stain Hoechst 3342 (Invitrogen) for 10 min. at room temperature. After the final incubation, cells were washed twice with PBS and covered with ProLong Gold (Molecular Probes) anti-fade solution for imaging. Images were recorded with an Olympus FluoView 1000 Inverted IX81 microscope, using a 40× oil objective using identical exposure and PMT settings for each primary antibody-fluorophore pair across the different treatment conditions.

### Flow cytometry

Cells were washed twice with ice-cold PBS, trypsinized, and counted. Next, the cells were resuspended in PBS to a final concentration of 1 × 10^6^ cells/ml. A total of 100 ul of the cell suspension was aliquoted into microcentrifuge tubes. For live/dead staining, we added 1ul of Zombie Violet stain (BioLegend, 423113) into each tube and incubated for 30 min. at room temperature in the dark (all subsequent steps were performed under minimal light conditions). The cells were then washed with ice-cold incubation buffer (0.5% BSA in PBS with 0.01% Sodium Azide) by centrifugation at 200 g for 1 min. and fixed with a freshly prepared 4% paraformaldehyde solution in PBS (pH = 6.9) for 10 min. at 37 °C. The samples were placed on ice for 1 min., washed with incubation buffer and subsequently permeabilized with 0.5% Triton X-100 in PBS for 20 min. on ice. For primary antibody incubation, cells were washed twice with incubation buffer and resuspended in primary antibodies diluted in incubation buffer (CDH 1:200, DSP 1:200, VIM-647 1:50) for 1 hour at room temperature. For secondary antibody staining (CDH and DSP), cells were washed twice with incubation buffer and incubated in isotype-specific Alexa488 secondary antibody diluted (1:1000) in incubation buffer for 30 min. at room temperature. After completion of all staining steps, the cells were washed with incubation buffer and resuspended in 500 ul incubation buffer. The samples were then analyzed on an LSRII flow cytometer system (BD Biosciences) with 1 × 10^4^ events recorded per sample.

## Additional Information

**How to cite this article**: Nath, A. *et al.* Elevated free fatty acid uptake via CD36 promotes epithelial-mesenchymal transition in hepatocellular carcinoma. *Sci. Rep.* **5**, 14752; doi: 10.1038/srep14752 (2015).

## Supplementary Material

Supplementary Information

## Figures and Tables

**Table 1:** Multiple linear regression analysis of TCGA LIHC data.

**y = BMI**	**Coefficients**		**Sig.**	**95.0% Confidence Interval for B**	
	**B**	**Std. Error**		**Lower Bound**	**Upper Bound**
CD36	0.309	0.474	0.516	−0.629	1.246
FABP1	0.430	0.263	0.105	−0.091	0.950
FABP4	0.172	0.325	0.597	−0.470	0.814
FABP5	1.065	0.558	0.058	−0.038	2.168
SLC27A2	0.208	0.367	0.572	−0.517	0.933
SLC27A5	−0.081	0.359	0.822	−0.790	0.629
CDH2	0.525	0.671	0.435	−0.801	1.850
FN1	2.629	0.953	0.007	0.746	4.512
SNAI1	0.236	0.499	0.637	−0.751	1.223
SNAI2	-0.207	0.516	0.689	−1.227	0.812
TWIST1	0.025	0.434	0.954	−0.833	0.883
TWIST2	−0.305	0.450	0.499	−1.197	0.587
VIM	0.831	0.815	0.309	−0.778	2.440
ZEB1	0.472	0.976	0.629	−1.457	2.401
ZEB2	1.166	0.650	0.075	−0.119	2.451
CDH1	0.205	0.503	0.684	−0.789	1.199
CLDN4	0.319	0.234	0.175	−0.143	0.781
CLDN7	0.491	0.392	0.213	−0.284	1.267
MUC1	0.568	0.316	0.074	−0.055	1.192
TJP3	0.435	0.331	0.190	−0.218	1.089
Bonferroni corrected p−value	0.003				
y = EMT score					
CD36	1.498	0.486	0.002	0.537	2.458
FABP1	−0.762	0.265	0.005	−1.286	−0.238
FABP4	1.580	0.318	0.000	0.952	2.208
FABP5	1.481	0.590	0.013	0.316	2.647
SLC27A2	−0.469	0.385	0.226	−1.230	0.293
SLC27A5	0.271	0.378	0.474	−0.475	1.017
Bonferroni corrected p−value	0.008				

P-values controlled for BMI, gender, age, and staging (1–2 = low grade, 3–4 = high grade (n = 158).

P-values controlled for gender, age, and staging (n = 158).

**Table 2:** Multiple linear regression analysis of human protein data.

**y = CD36**	**Coefficients**		**Sig.**	**95.0% Confidence Interval for B**	
	**B**	**Std. Error**		**Lower Bound**	**Upper Bound**
VIM	0.558	0.301	0.090	−0.104	1.220
ZEB1	1.595	0.142	0.000	1.282	1.907
ZEB2	1.121	0.326	0.006	0.403	1.838
TGFB	0.969	1.148	0.417	−1.558	3.497
PORCN	0.282	0.233	0.252	−0.231	0.796
SNAIL	0.292	0.509	0.577	−0.827	1.411
Bonferroni corrected p-value	0.008				
Confounding variables: BMI, Sex, grade (n = 16)					
y = BMI					
CD36	1.917	3.499	0.594	−5.706	9.540
VIM	5.810	3.229	0.097	−1.226	12.846
ZEB1	1.337	5.938	0.826	−11.601	14.275
ZEB2	−0.525	5.580	0.927	−12.684	11.633
TGFB	24.406	6.908	0.004	9.356	39.457
PORCN	2.828	2.708	0.317	−3.072	8.727
CDH	3.344	8.586	0.704	−15.363	22.051
DSP	−13.061	12.515	0.317	−40.329	14.207
SNAIL	7.881	5.131	0.150	−3.298	19.060
Bonferroni corrected p-value	0.006				

Confounding variables: Sex, Grade (1–2 = low grade, 3–4 = high grade) (n = 16).

**Table 3:** List of primers for qRT-PCR.

**Primer**	**Sequence (5′->3′)**
*SNAIL* Forward	CACTATGCCGCGCTCTTTC
*SNAIL* Reverse	GCTGGAAGGTAAACTCTGGATTAGA
*SLUG* Forward	GGACACATTAGAACTCACACGGG
*SLUG* Reverse	GCAGTGAGGGCAAGAAAAAGG
*ZEB1* Forward	ATGCAGCTGACTGTGAAGGT
*ZEB1* Reverse	GAAAATGCATCTGGTGTTCC
*ZEB2* Forward	TATGGCCTACACCTACCCAAC
*ZEB2* Reverse	AGGCCTGACATGTAGTCTTGTG
*TWIST* Forward	GCAGGGCCGGAGACCTAG
*TWIST* Reverse	TGTCCATTTTCTCCTTCTCTGGA
*FOXC2* Forward	GCAGGGCTGGCAGAACAG
*FOXC2* Reverse	CGCGGCACTTTCACGAA
*GAPDH* Forward	CAGCCGCATCTTCTTTTGCG
*GAPDH* Reverse	TGGAATTTGCCATGGGTGGA

**Table 4:** List of antibodies for western blots and immunofluorescence staining.

**Antibody**	**Source (catalog)**	**Western Blot Dilutions**
ZEB1	Origene (TA802298)	1:2000
ZEB2	Origene (TA802113)	1:2000
PORCN	Millipore (MABS21)	1:2000
CD36	Millipore (CBL168)	1:2000
SNAI1	SantaCruz (sc28199)	1:1000
DSP	Abcam (ab71690)	1:500
CDH1	Cell Signaling (3195)	1:1000
VIM	Cell Signaling (3932)	1:1000
TGFB	Cell Signaling (3711)	1:1000
GAPDH	Cell Signaling (5174)	1:2000
VIM-647	Cell Signaling (9856)	−
CTNNB1	Cell Signaling (8480)	−
SMAD2/3	Cell Signaling (8685)	−
