# Supplementary material for: Elevated free fatty acid uptake via CD36 promotes epithelial-mesenchymal transition in hepatocellular carcinoma
Source: Sci Rep. 2015 Oct 1;5:14752. doi: 10.1038/srep14752 (PMC4589791; doi:10.1038/srep14752)
Supplement: Supplementary Information [file srep14752-s1.pdf]

Elevated free fatty acid uptake via CD36 promotes epithelial-mesenchymal transition in hepatocellular carcinoma

Aritro Nath[1], Irene Li[2], Lewis R. Roberts[3] and *Christina Chan[1, 2, 4]

[1] Genetics Program, Michigan State University, 567 Wilson Road, Rm 2240E, East Lansing, Michigan 48824, USA

[2] Department of Microbiology and Molecular Genetics, Michigan State University, 567 Wilson Road, Rm 2215, East Lansing, Michigan 48824, USA

[3] Division of Gastroenterology and Hepatology, Mayo Clinic College of Medicine, 200 First St. SW, Rochester, Minnesota 55905, USA

[4] Department of Chemical Engineering and Materials Science, Michigan State University, 428 South Shaw Lane, Rm 2527, East Lansing, Michigan 48824, USA

*Corresponding author

krischan@egr.msu.edu

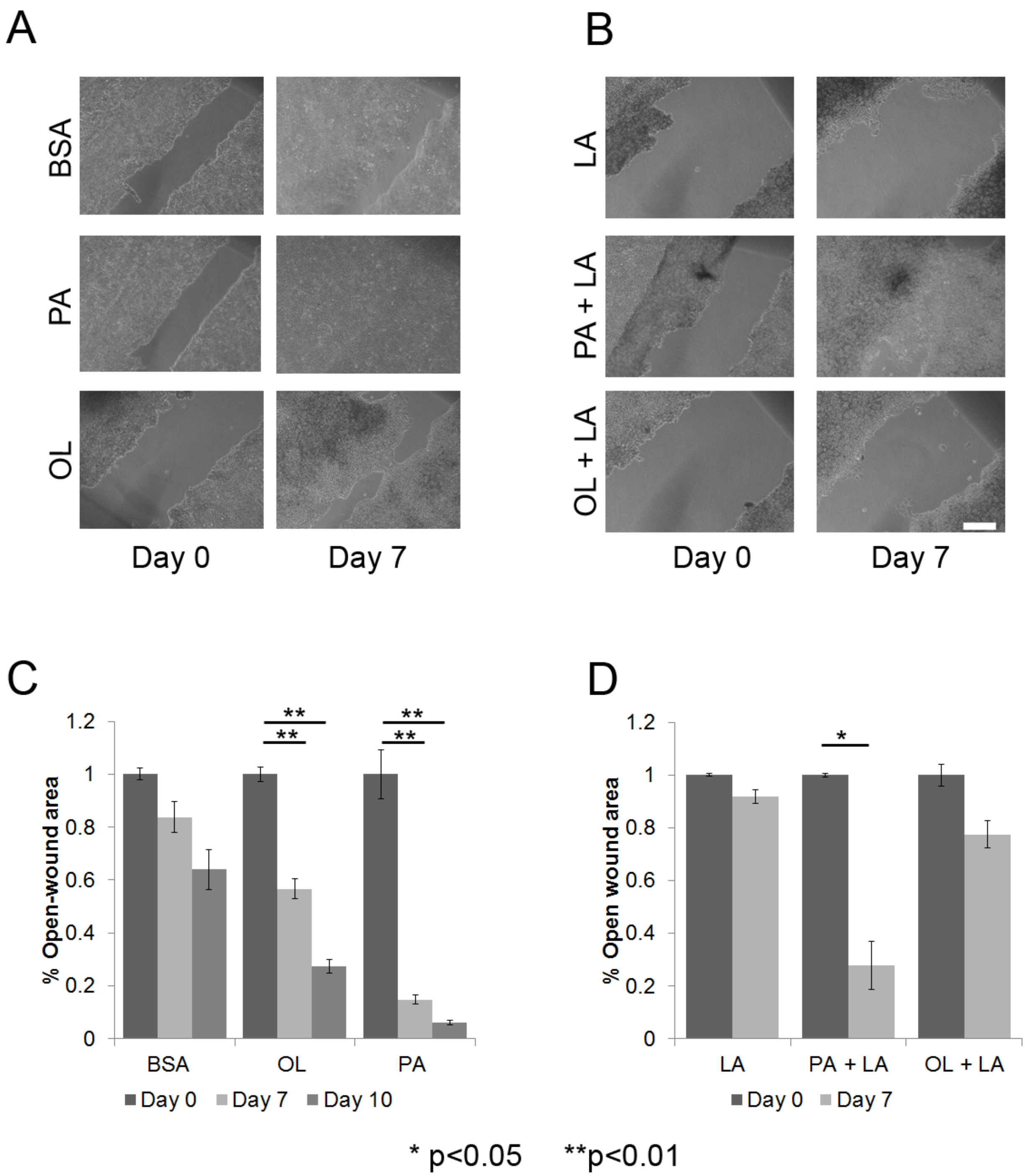

Supplementary Figure 1: Differential effects of FFAs on migration. A & B. Scratch-wound healing assays. HepG2 cells were grown to 80% confluence in regular medium, and a scratch was introduced on the monolayer. The cells were then cultured in regular media containing either

BSA (control), PA (0.4mM), OL (0.4mM), LA (0.4mM), PA (0.2mM) + LA (0.2mM) or OL (0.2mM) + LA (0.2mM). The migration of the wound front was monitored over a 10-day period to establish an empirical time-frame within which the cell front acquired the ability to migrate across the wound. Phase contrast images were recorded at 40X magnification (scale bar=50µm) C & D. Bar graph showing the open wound area in treated cells calculated using the T-scratch package at different time points normalized to open wound area at 0 hr. P-values indicate significance levels (n=3) determined by Student’s t-test (two tailed).

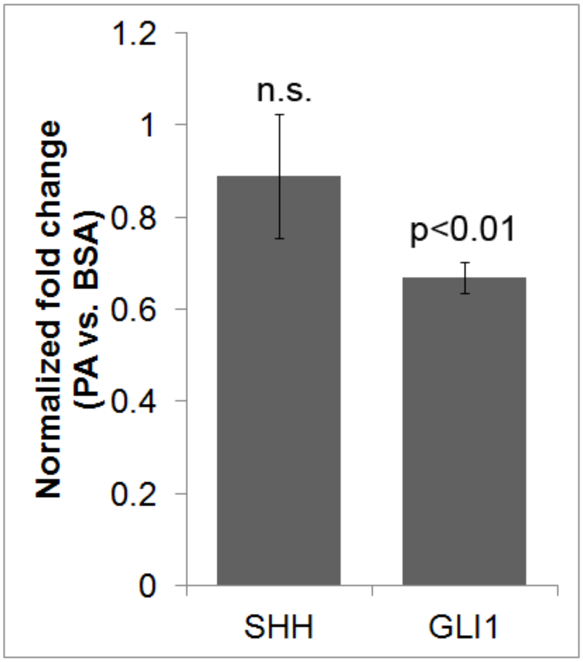

Supplementary Figure 2 mRNA expression levels of Hedgehog ligand *SHH* and downstream transcription factor *GLI1*in PA vs. BSA treated HepG2 cells. Bar graphs showing fold-change in *GAPDH* normalized expression levels of *SHH* and *GLI1*in PA vs. BSA treated HepG2 cells. P-value indicates comparison of average delta Ct values between PA and BSA using two tailed Student's t-test.

Supplementary Figure 3

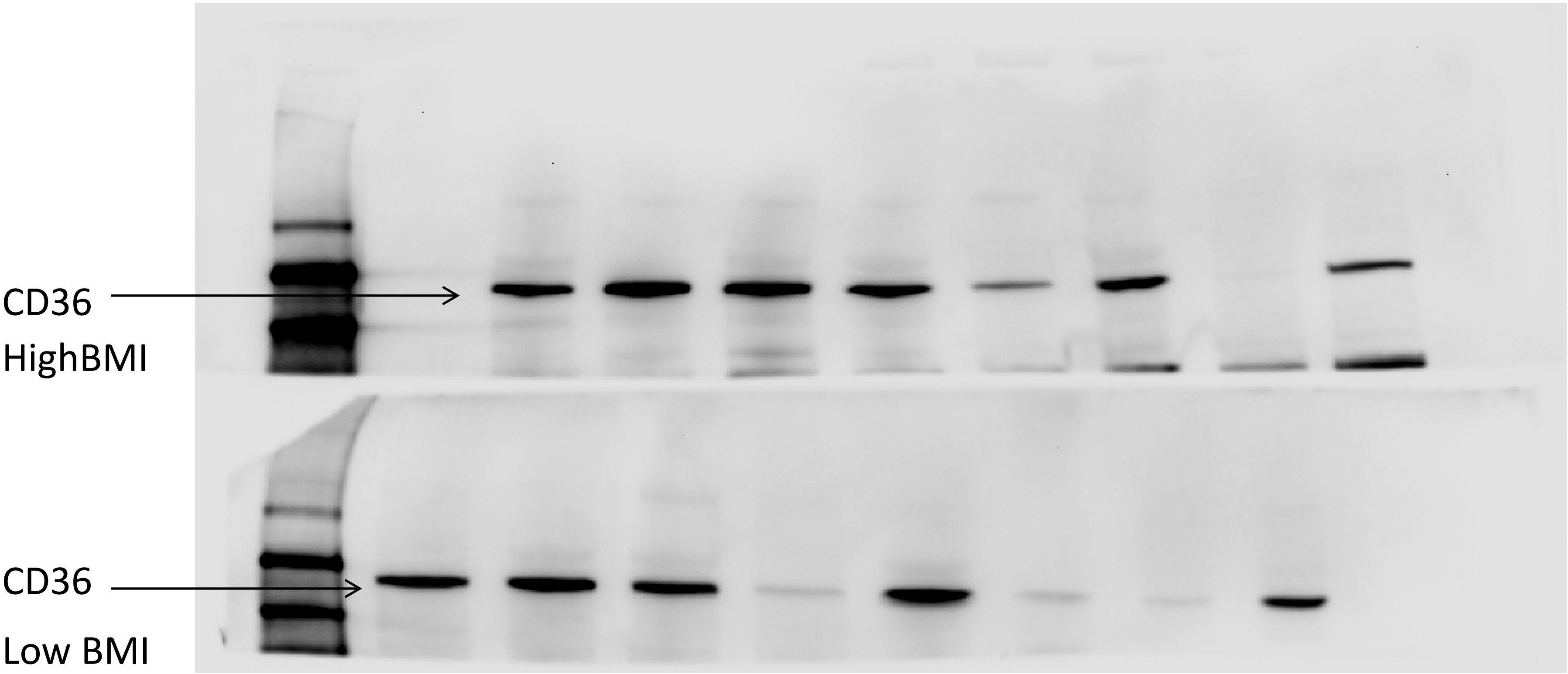

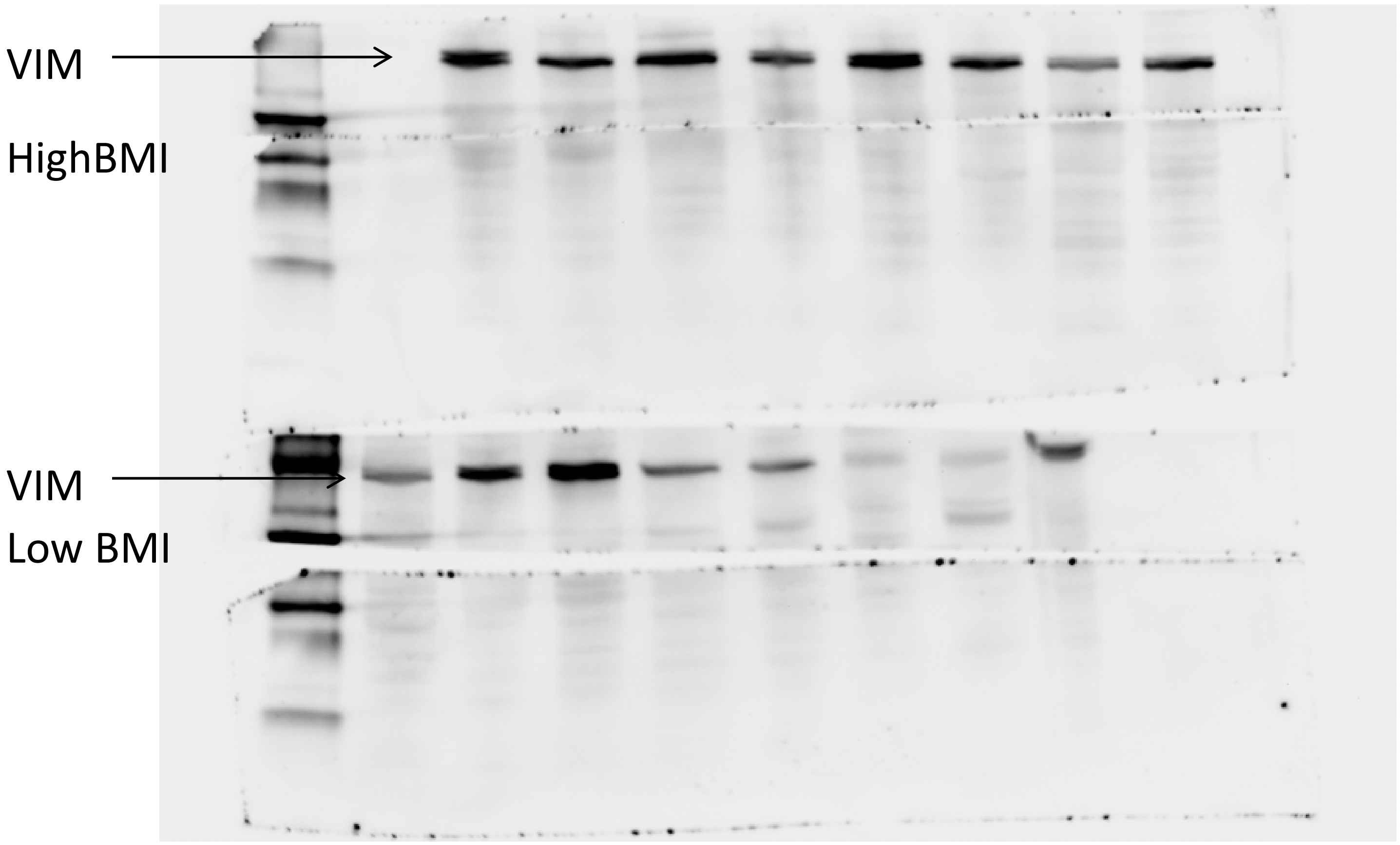
VIM
HighBMI
VIM
Low BMI

CDH1

HighBMI

CDH1

Low BMI

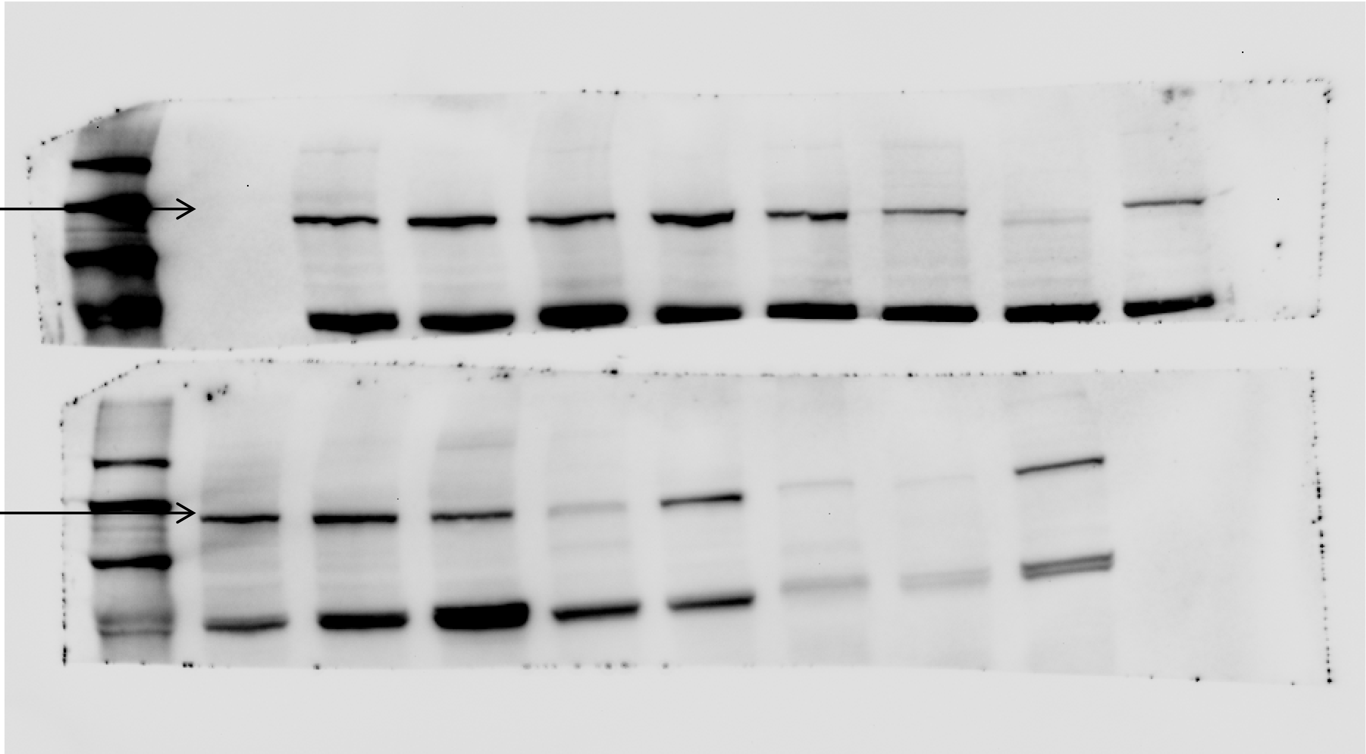

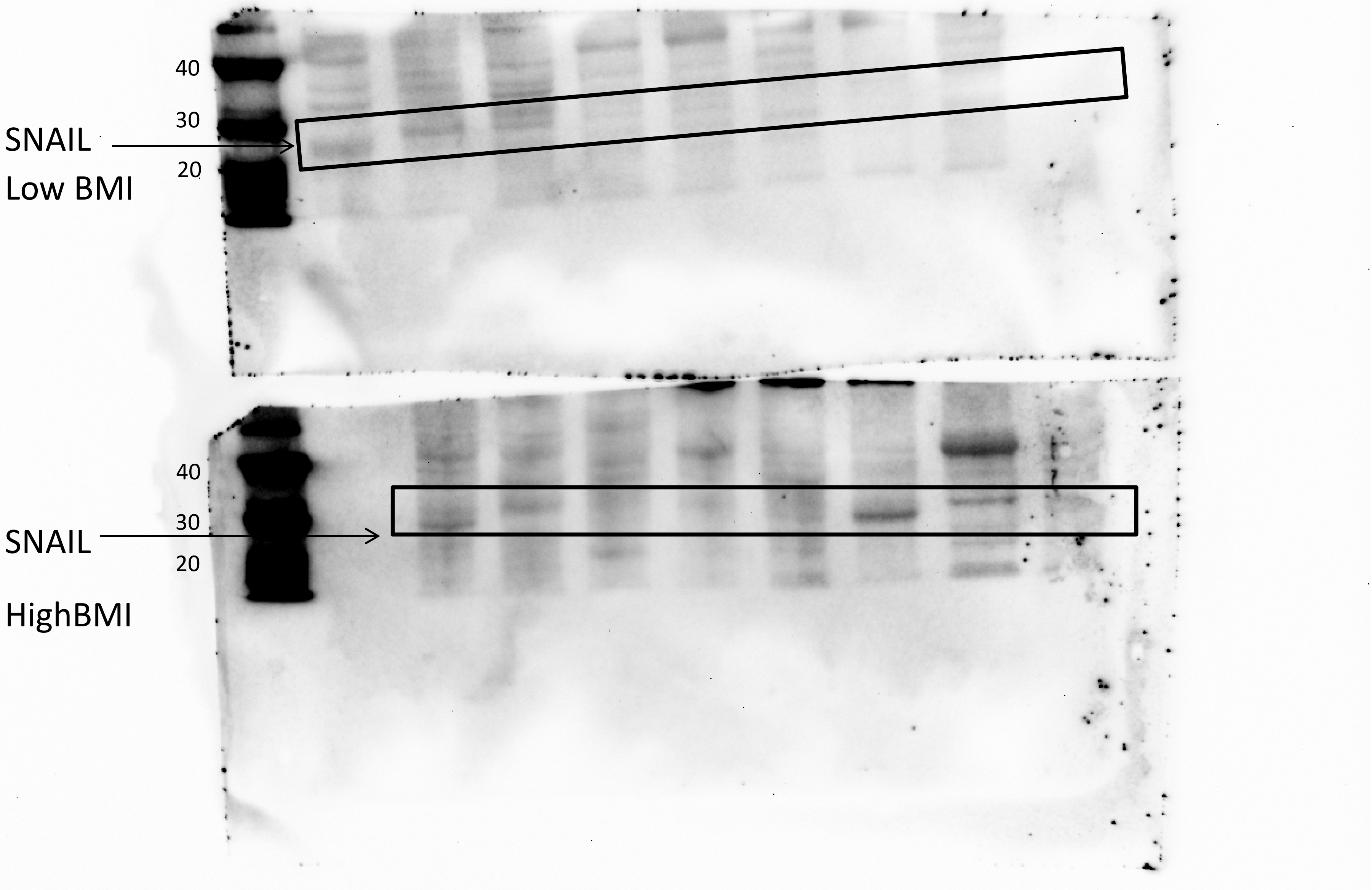
40
30
SNAIL
20
Low BMI
40
30
SNAIL
20
HighBMI

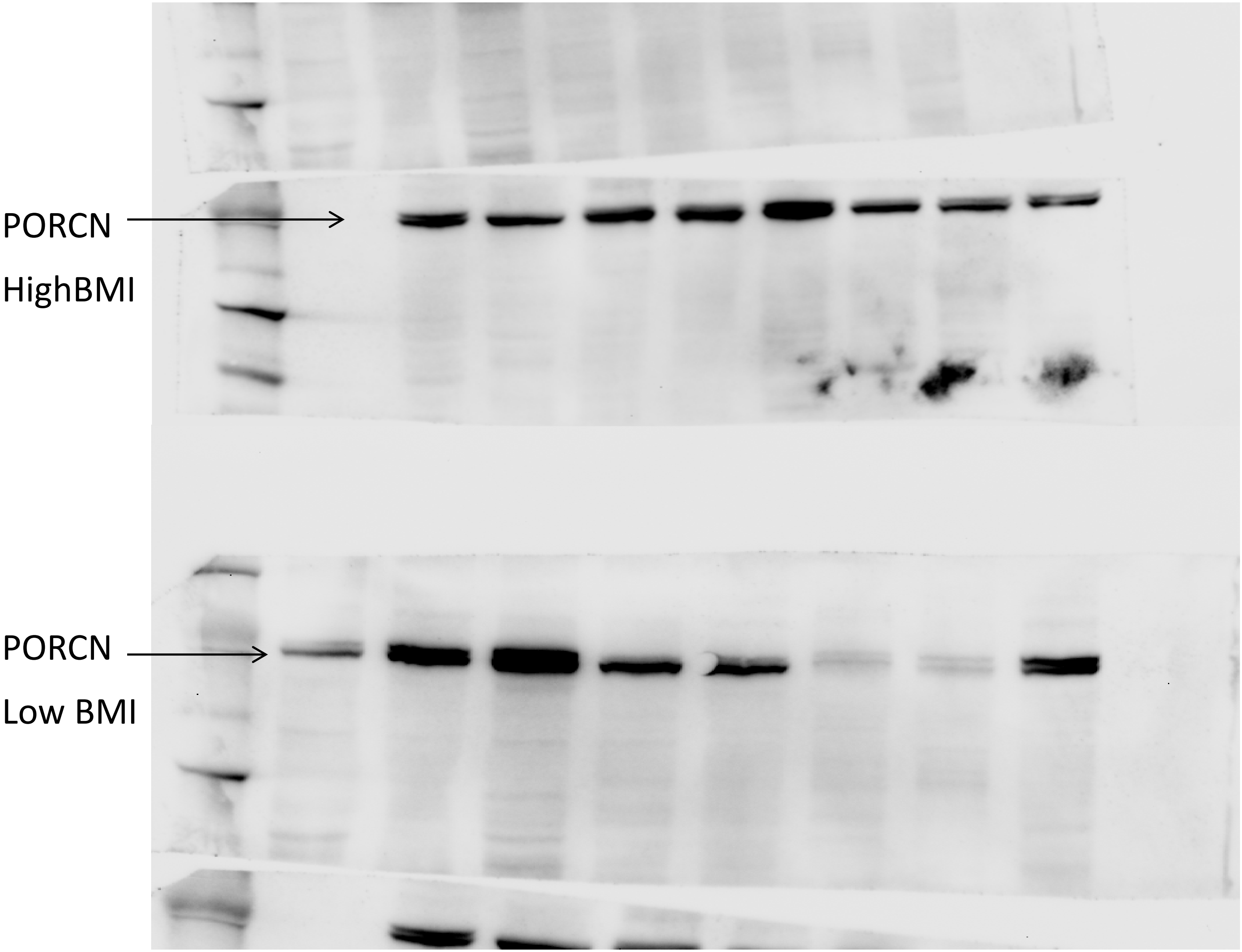
PORCN
HighBMI
PORCN
Low BMI

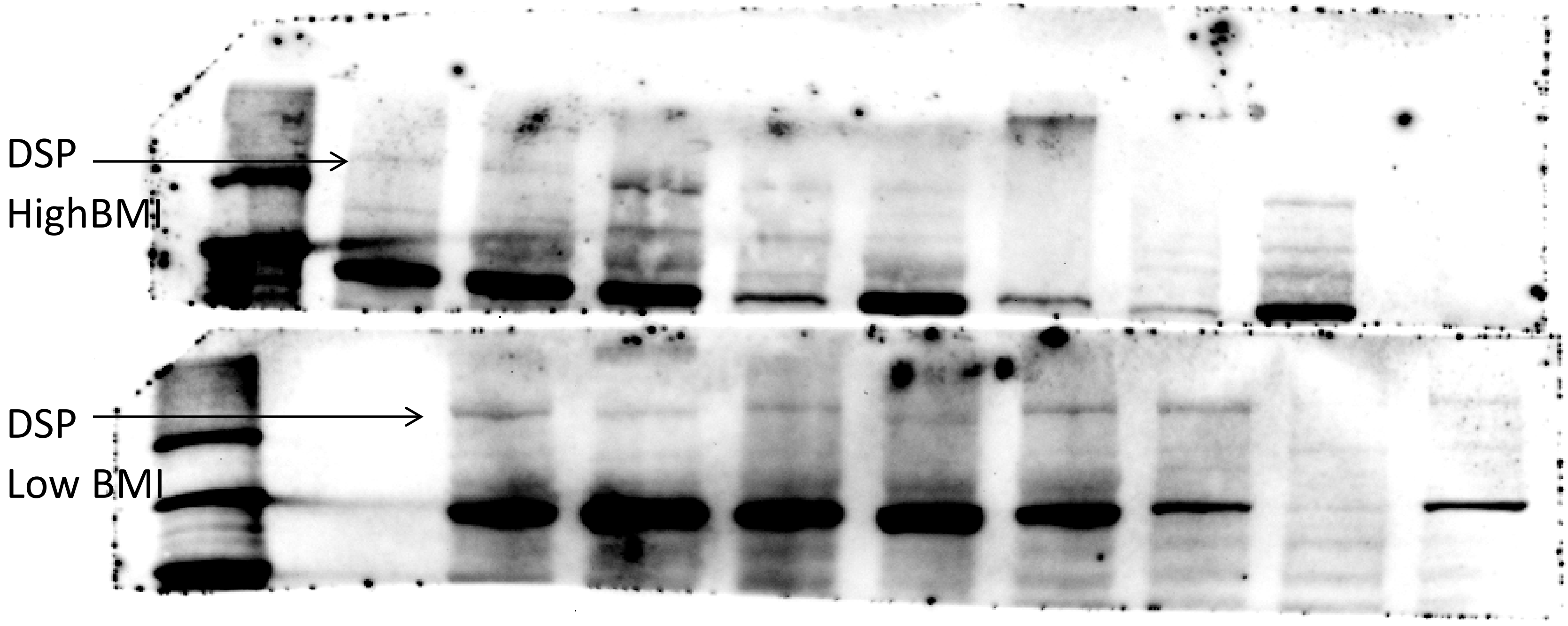
DSP
HighBMI
DSP
Low BMI

TGFB

Low BMI

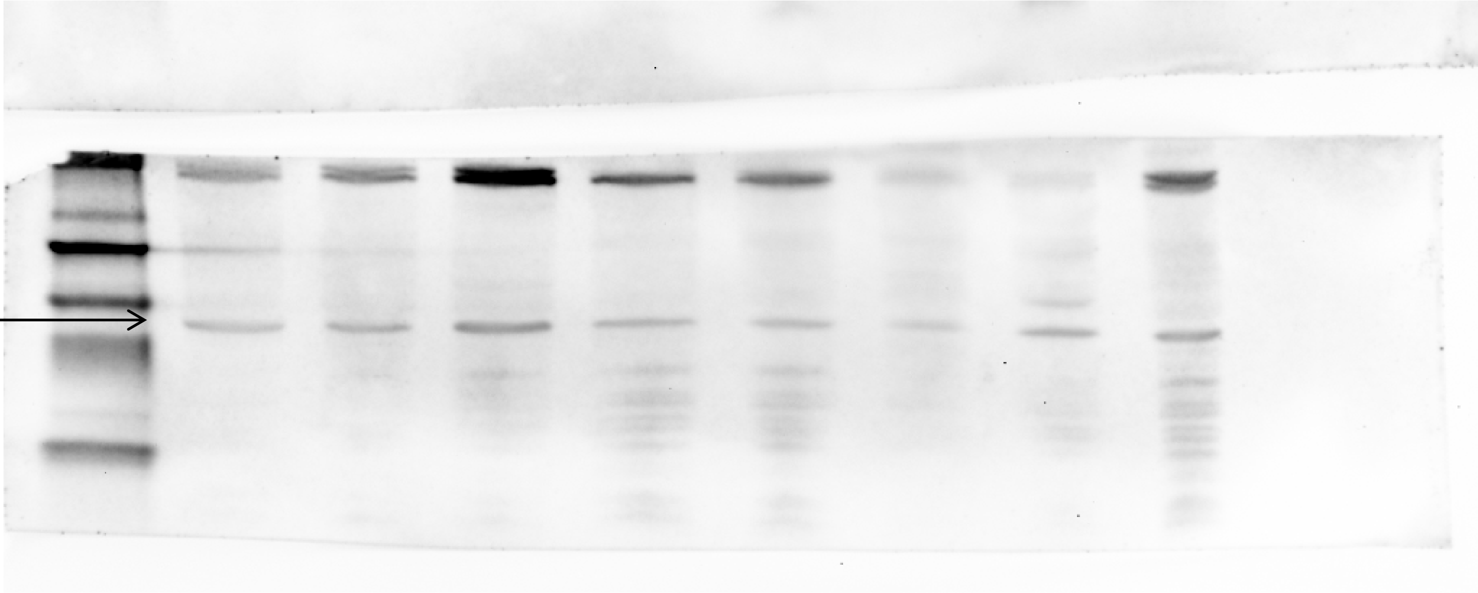

TGFB

HighBMI

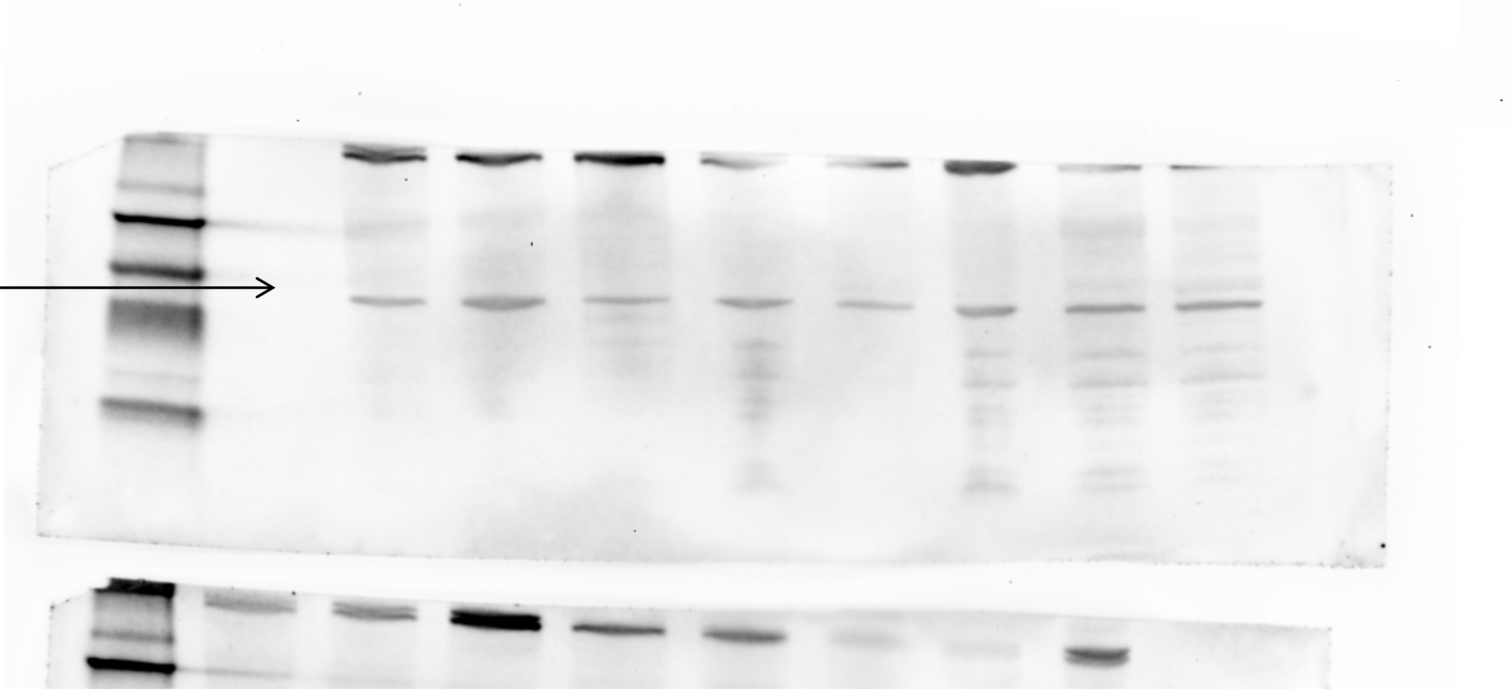

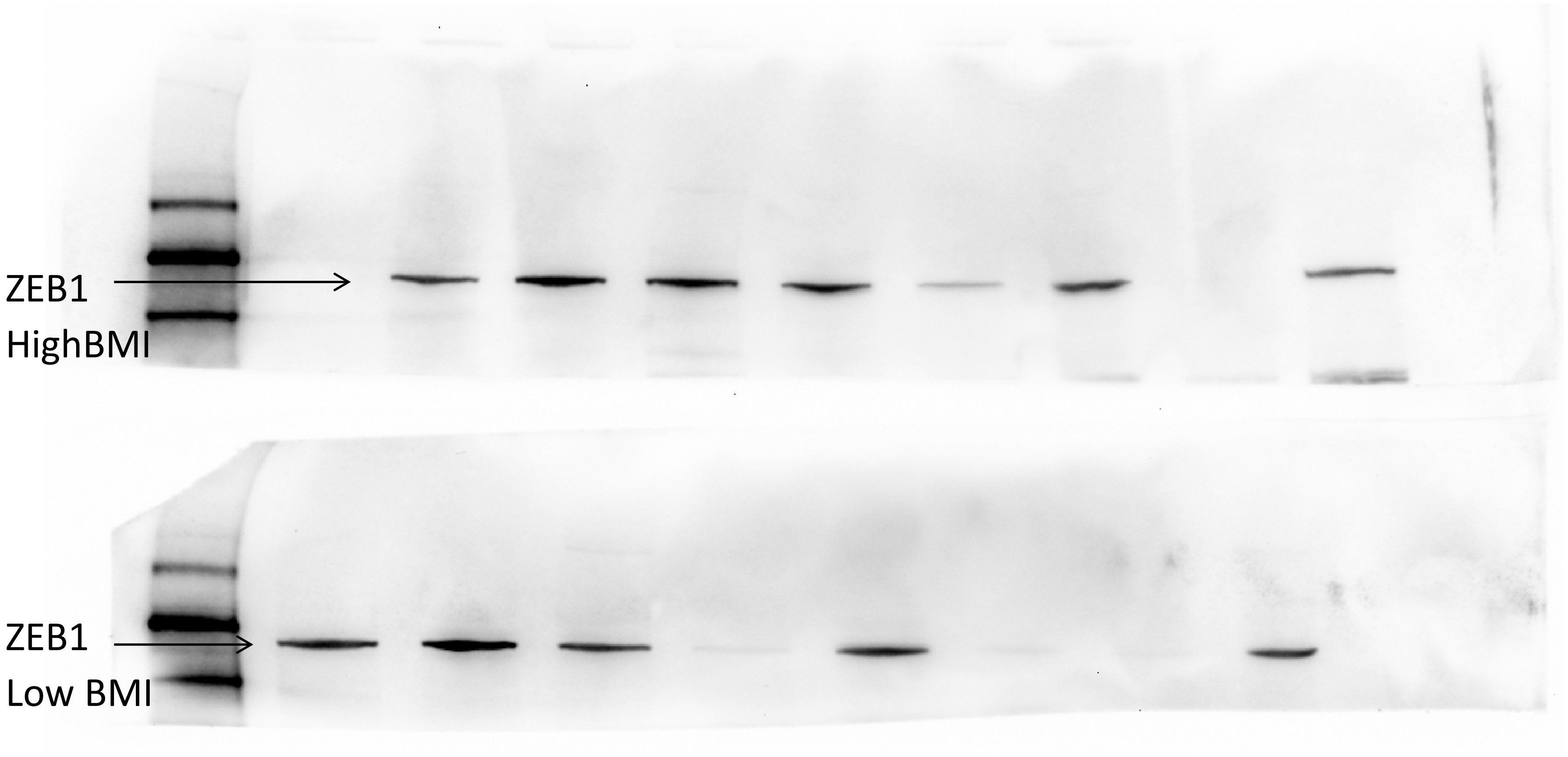
ZEB1
HighBMI
ZEB1
Low BMI

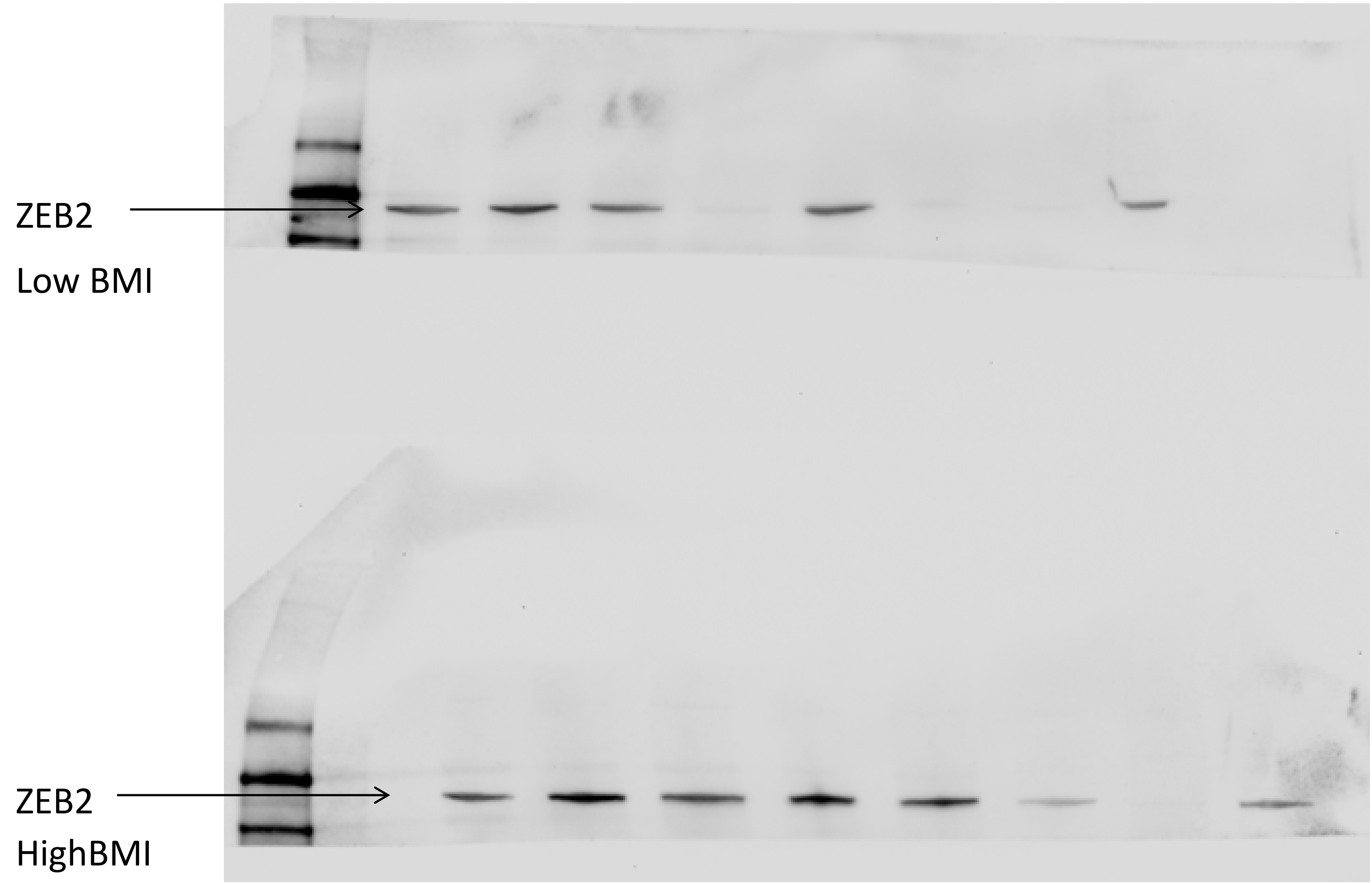

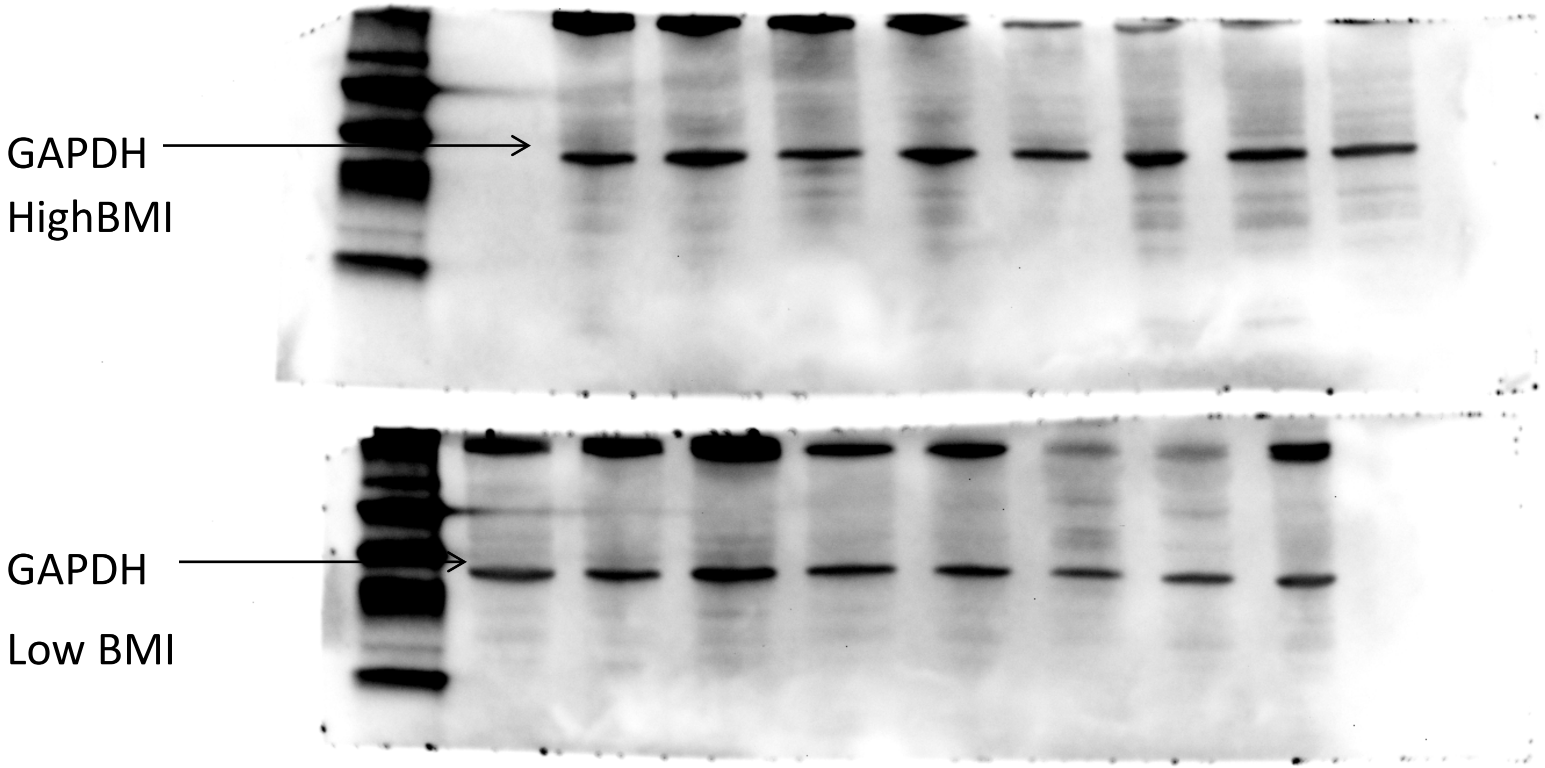
GAPDH
HighBMI
GAPDH
Low BMI

Supplementary Figure 4

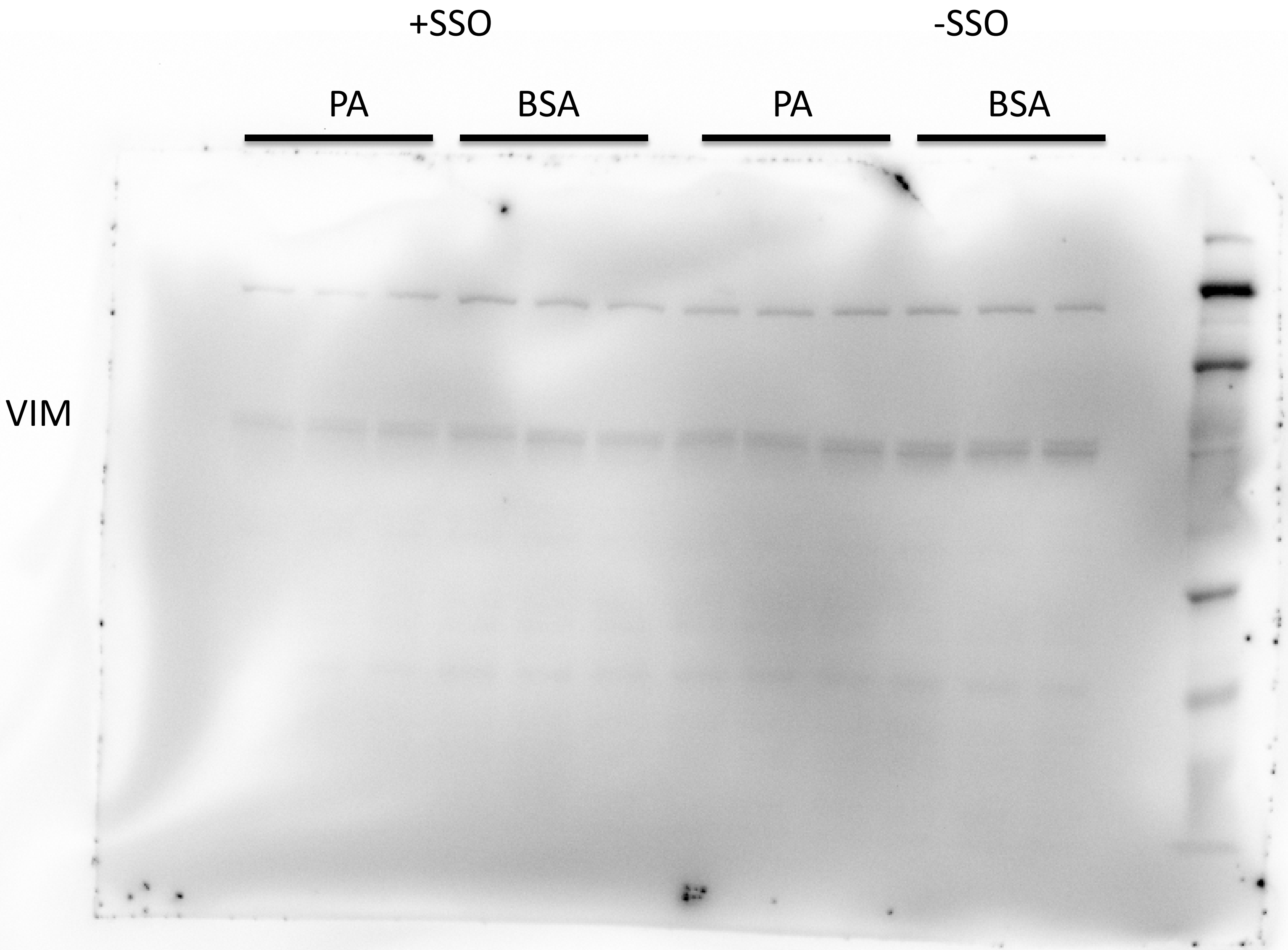

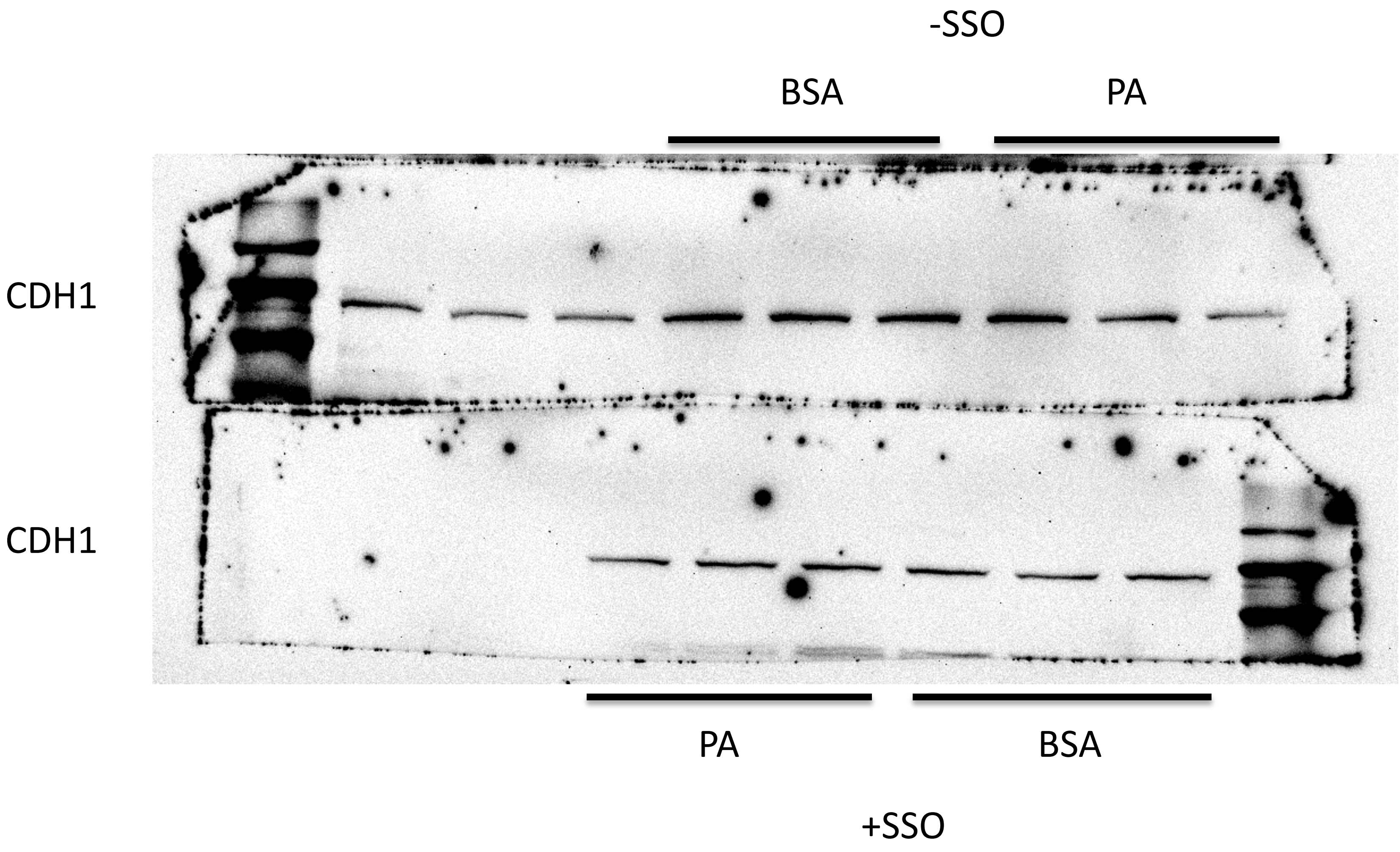
-SSO
BSA
PA
CDH1
CDH1
PA
BSA
+SSO

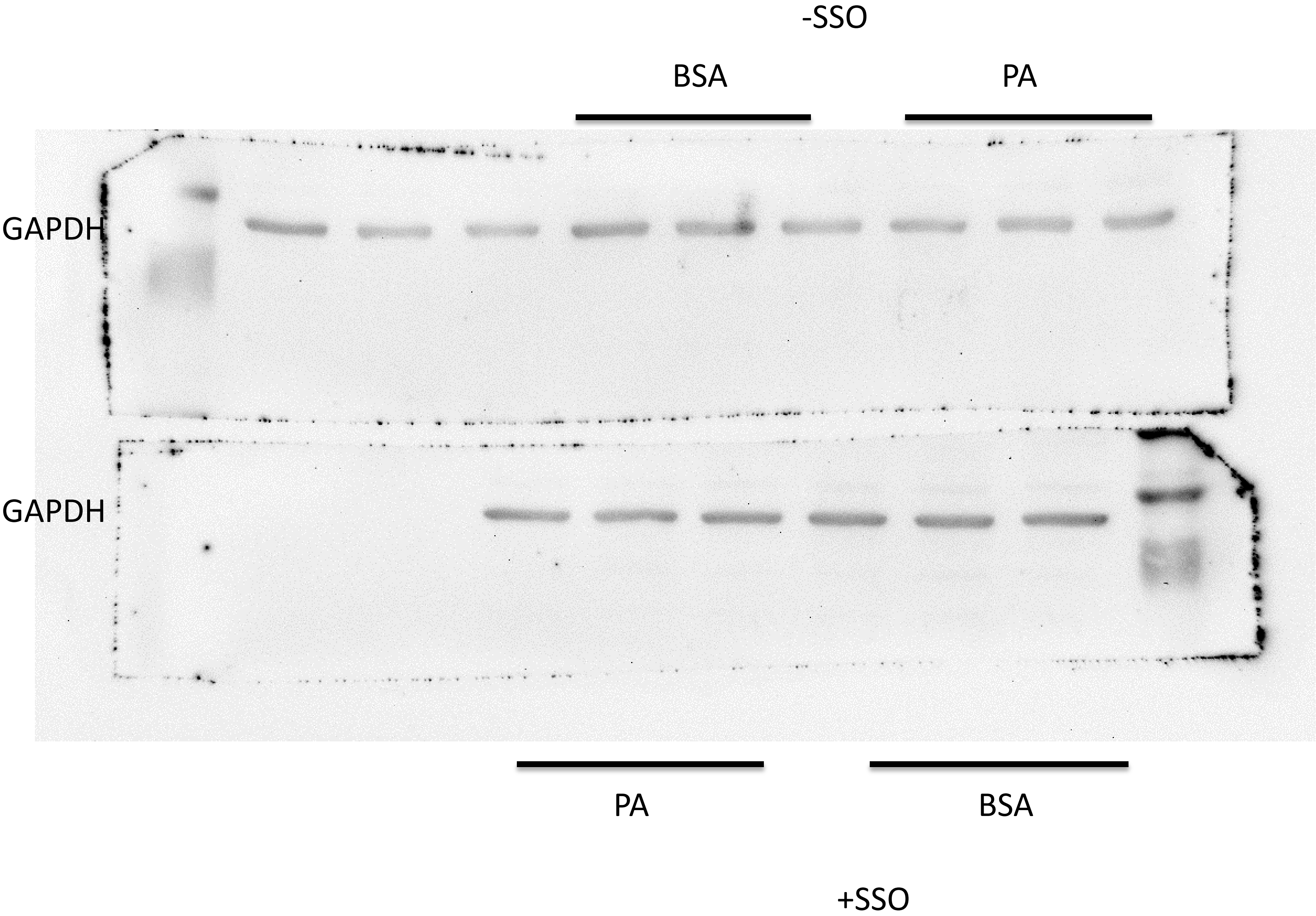
-SSO
BSA
PA
GAPDH
GAPDH
PA
BSA
+SSO

Supplementary Figure 5

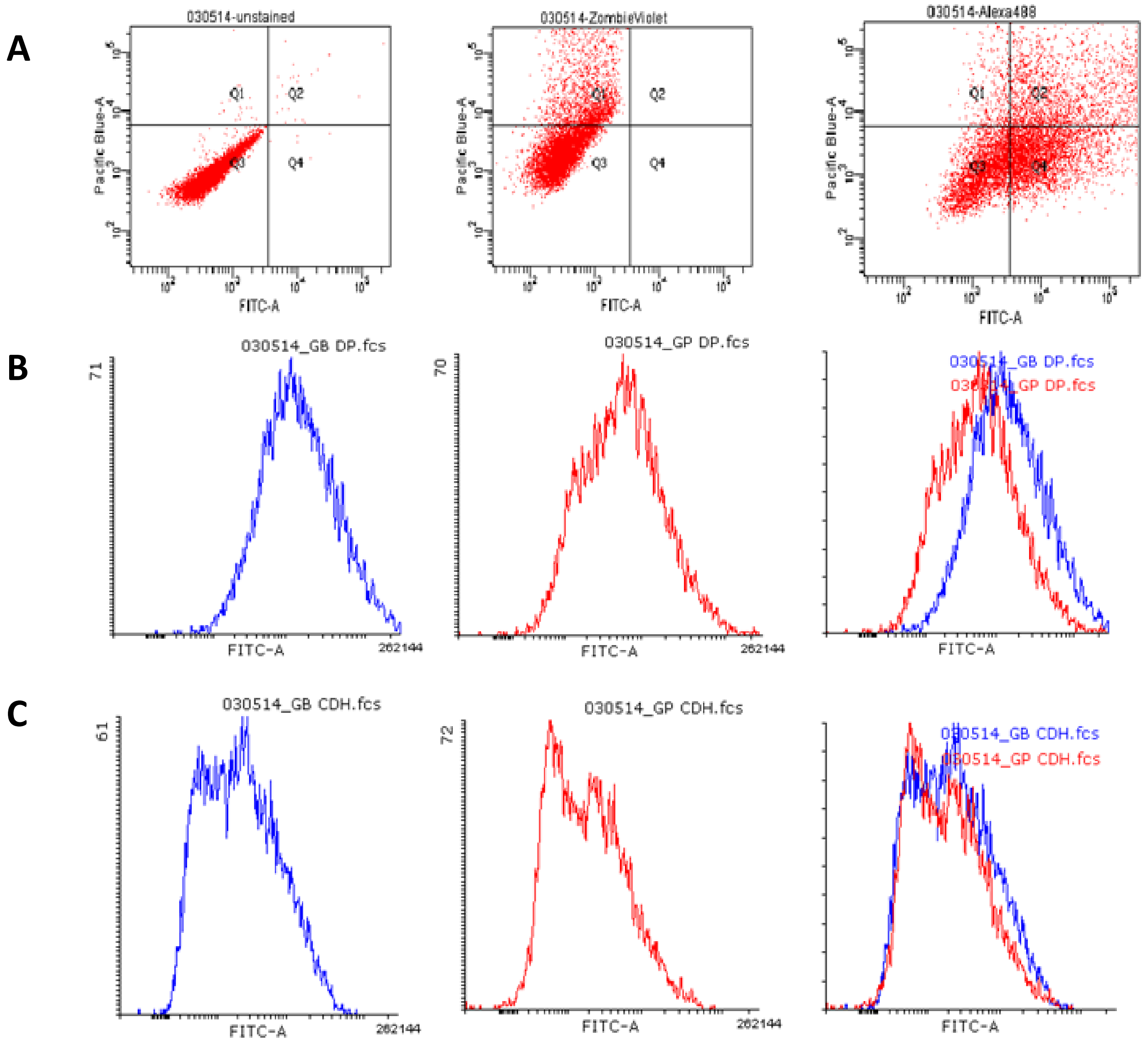

Supplementary Figure 5: A. (From left to right) Panels showing unstained control to set gating threshold Zombie Violet stained cells and GAPDH-Alexa488 stained cells. This gating was used for the subsequent marker assessment. The plot distributions show the signal intensity distributions for **B**. DSP and **C**. CDH1 from BSA (BLUE) or PA (RED) treated cells. The overlap images show that the DSP peak in the PA treated cells is clearly shifted towards the left, indicating a population of cells with reduced DSP expression in PA treated condition. The CDH1 plots show a distinct bi-modal distribution, where the low-intensity peak (lower CDH expression) is higher in the PA treated cells, while the high-intensity peak (higher CDH expression) is higher in the control cells, and is suggestive of sub-population of cells.

Supplementary Table 1

Sample_ID	CD36	FABP1	FABP4	FABP5	SLC27A2	SLC27A5	CDH2	FN1	SNAI1	SNAI2	TWIST1	TWIST2	VIM	ZEB1	ZEB2	CDH1	CLDN4	CLDN7	MUC1	TJP3	EMTSCR	Age	Gender	Gender_bin	Height	Weight	BMI	Pathologic M	Pathologic N	Pathologic T	Pathologic_T_bin	Pathologic Stage
TCGA-BC-4072	10.501	13.571	0.827	7.397	10.286	10.243	11.947	16.748	5.973	7.891	3.549	1.965	12.783	9.206	9.312	12.776	10.957	10.027	4.981	9.615	31.018	74	FEMALE	0				M0	N0	T3	1	Stage IIIA
TCGA-BC-4073	11.302	14.306	5.742	6.633	9.226	8.344	10.975	17.594	6.315	5.916	3.516	1.194	13.305	9.812	9.52	13.298	11.673	8.292	8.258	9.694	26.933	73	MALE	1				M0	N0	T3	1	Stage IIIA
TCGA-BC-A10Q	4.275	6.168	-1.118	1.882	11.589	6.162	8.881	16.395	6.398	7.077	2.204	1.69	14.042	8.537	7.816	14.609	13.553	13.337	7.062	10.448	14.031	72	FEMALE	0				MX	NX	T2	0	
TCGA-BC-A10R	6.628	13.138	6.432	3.45	12.195	10.613	11.631	17.655	7.167	7.503	0.713	1.45	13.246	10.092	8.352	13.202	6.139	8.05	6.128	8.916	35.373	66	FEMALE	0				MX	NX	T3	1	
TCGA-BC-A10S	10.447	16.316	10.195	6.337	13.338	14.553	11.525	17.002	8.054	8.266	1.994	0.187	13.84	8.452	8.447	11.84	6.367	11.35	6.416	9.281	32.513	81	MALE	1		74		MX	NX	T3	1	
TCGA-BC-A10T	10.477	14.131	7.016	3.72	12.357	14.276	11.989	16.872	8.461	7.707	2.819	1.913	13.979	9.591	9.245	11.608	6.667	9.921	6.179	6.843	41.356	76	MALE	1		86		MX	NX	T4	1	
TCGA-BC-A10U	8.062	13.438	1.479	3.752	10.878	11.827	11.4	16.843	3.649	8.724	-1.106		11.312	8.844	6.976	10.623	11.391	9.223	6.615	7.252	21.539	69	MALE	1				MX	NX	T2	0	
TCGA-BC-A10W	10.371	12.851	1.374	6.137	8.997	8.376	11.026	17.681	4.776	6.611	2.567		12.495	9.688	8.555	11.928	11.712	9.362	5.106	8.38	26.911	50	MALE	1		71		MX	NX	T4	1	
TCGA-BC-A10X	10.078	15.008	4.774	3.13	12.798	14.073	11.858	17.754	8.533	8.667	3.653	2.589	12.87	9.711	9.768	13.227	7.861	8.339	5.606	7.858	42.513	52	FEMALE	0		65		MX	N0	T3a	1	Stage IIIA
TCGA-BC-A10Y	11.201	11.795	6.443	5.183	12.671	13.443	11.422	15.718	3.98	6.085	0.52	-1.065	12.543	8.65	7.478	12.73	5.842	10.929	6.723	8.62	20.488	76	MALE	1	175	77	25.14286	MX	NX	T4	1	
TCGA-BC-A10Z	11.078	13.362	9.215	1.787	13.426	12.793	12.409	16.74	4.431	4.487	0.372		10.533	8.442	6.387	12.692	8.169	9.78	-0.213	3.831	29.543	62	FEMALE	0		79		MX	N0	T1	0	Stage I
TCGA-BC-A110	9.773	15.204	9.313	6.544	12.97	14.785	10.679	16.256	9.15	7.663	3.415	3.415	14.534	8.973	8.977	12.393	8.746	10.193	6	8.465	37.266	51	FEMALE	0	155	91	37.87721	MX	NX	T1	0	
TCGA-BC-A112	9.069	9.59	3.005	5.826	6.175	6.776	11.593	18.59	6.558	8.512	2.799	2.099	13.2	9.216	8.706	13.006	12.551	11.009	11.422	9.505	23.779	80	MALE	1	165	97	35.62902	MX	NX	T3	1	
TCGA-BC-A216	11.366	13.841	7.027	6.303	11.579	10.678	10.709	16.688	3.419	7.27	4.275	-0.973	11.944	8.983	8.511	11.406	10.722	10.582	9.357	9.969	18.792	62	FEMALE	0	164	151	56.14218	M0	NX	T3	1	Stage IIIA
TCGA-BC-A217	8.589	7.978	3.952	1.805	10.014	11.692	11.797	15.799	4.283	7.821	0.583		11.366	8.887	6.935	11.923	6.964	10.629	7.51	8.616	21.831	75	FEMALE	0	163	81	30.48666	M0	NX	T2	0	Stage II
TCGA-BC-A3KF	11.16	15.36	6.933	3.692	12.968	12.537	11.445	16.324	3.777	9.969	-1.352		12.057	8.52	7.285	14.104	8.29	10.679	-0.352	9.323	25.981	66	FEMALE	0	173	58	19.3792	M0	NX	T1	0	Stage I
TCGA-BC-A3KG	11.667	6.336	4.271	4.679	13.084	7.759	12.103	15.974	2.357	4.978	1.826	-1.344	11.819	9.422	6.909	10.393	0.978	7.73	3.241	7.698	34.004	68	FEMALE	0	159	64	25.31545	M0	N0	T2	0	Stage II
TCGA-BC-A5W4	7.229	15.12	5.796	2.537	11.027	13.782	11.592	16.39	1.314	9.636			10.147	6.6	4.314	12.956	12.907	11.159	2.73	7.051	13.191	69	MALE	1	183	106	31.65218	M0	NX	T3a	1	Stage IIIA
TCGA-BC-A69H	9.947	15.68	4.59	9.206	10.01	10.147	11.287	15.663	6.488	6.912	2.546		13.191	6.736	6.775	11.36	11.904	10.225	4.404	8.914	22.79	64	MALE	1	173	70	23.38869	M0	NX	T2	0	Stage II
TCGA-BC-A69I	11.336	15.15	9.682	4.601	11.755	14.979	10.577	16.647	8.273	7.918	2.923	1.186	13.706	8.877	6.781	9.306	3.06	10.884	0.601	5.923	47.113	69	MALE	1	180	95	29.32099	M0	N0	T1	0	Stage I
TCGA-BD-A2L6	10.605	15.507	8.158	4.811	12.624	14.795	11.335	16.787	5.455	5.983	2.489	-0.971	12.82	8.241	7.199	12.844	8.678	10.619	2.029	9.067	26.102	69	MALE	1	168	110	38.97392	MX	NX	T2	0	
TCGA-BD-A3EP	9.483	13.702	7.196	5.846	12.033	13.18	11.2	16.82	8.11	6.419	-0.512	2.073	13.976	9.515	9.114	11.209	10.578	8.324	4.533	6.321	35.752	75	FEMALE	0	168	71	25.1559	M0	N0	T1	0	Stage I
TCGA-BD-A3ER	9.808	15.776	7.712	5.655	13.366	12.611	10.699	18.047	7.876	6.717	3.655	2.375	13.426	9.61	8.815	12.569	11.063	10.718	3.96	9.257	33.652	62	MALE	1	188	111	31.40561	MX	NX	T2	0	Stage II
TCGA-BW-A5NO	9.657	13.724	6.667	3.999	12.434	13.7	12.202	16.683	3.892	7.134	0.191	0.191	12.234	8.366	7.558	12.685	7.791	11.08	3.651	6.806	26.44	50	MALE	1				MX	NX	T3a	1	Stage IIIA
TCGA-BW-A5NP	10.531	14.244	4.772	5.408	10.039	7.884	11.065	15.204	3.135	4.772	0.965	0.55	11.53	9.747	6.693	11.344	3.919	8.316	5.535	9.462	25.085	26	FEMALE	0				M1	N0	T2	0	Stage IV
TCGA-BW-A5NQ	12.943	13.792	5.652	6.638	9.759	10.562	10.313	16.224	4.081	5.537	-0.048		12.646	8.256	8.23	10.908	7.483	8.226	1.952	5.475	31.194	63	MALE	1				MX	NX	T1	0	Stage I
TCGA-CC-5258	10.561	15.535	1.251	8.707	9.899	9.673	11.745	18.098	7.321	7.048	3.099		13.17	9.447	8.7	10.734	4.099	5.119	4.951	8.081	45.644	48	MALE	1		55		M0	N0	T2	0	Stage II
TCGA-CC-5259	8.636	17.306	5.732	5.331	10.958	14.658	11.443	17.088	4.388	5.08	2.272	1.687	12.059	7.71	5.642	12.051	8.302	8.226	5.08	7.272	26.436	60	FEMALE	0	153	49	20.93212	M0	N0	T4	1	Stage IIIC
TCGA-CC-5260	4.454	5.128	1.567	4.332	10.217	6.637	8.377	15.715	6.889	7.549	5.026	3.415	13.751	8.543	9.634	13.013	13.562	11.995	6.62	9.186	24.523	61	FEMALE	0	153	46	19.65056	M0	N0	T4	1	Stage IIIC
TCGA-CC-5261	6.986	10.85	4.542	5.386	11.337	9.55	11.695	16.581	7.64	7.898	4.313	4.542	13.752	9.368	9.77	13.403	11.672	11.345	4.584	10.024	34.532	44	MALE	1	175	58	18.93878	M0	N0	T2	0	Stage II
TCGA-CC-5262	9.132	14.875	7.176	6.481	13.034	13.001	11.36	17.304	6.885	7.267	4.916	1.653	14.196	8.394	8.4	10.736	5.138	7.714	4.189	8.319	44.277	67	MALE	1	170	62	21.45329	M0	N0	T4	1	Stage IIIC
TCGA-CC-5263	13.671	8.041	3.573	5.374	7.751	6.807	11.914	16.998	5.596	5.374	1.426	0.204	13.136	9.459	8.444	12.384	11.046	5.809	12.294	8.85	22.168	35	MALE	1	163	59	22.20633	M0	N0	T3	1	Stage IIIA
TCGA-CC-5264	10.076	13.692	3.049	5.25	9.185	9.145	10.722	17.171	5.063	5.503	0.727	-1.595	12.126	8.896	7.736	13.744	13.59	11.04	5.371	9.43	13.174	71	MALE	1	160	52	20.3125	M0	N0	T3	1	Stage IIIA
TCGA-CC-A1HT	7.475	5.287	0.439	7.501	9.458	6.928	12.118	15.741	6.888	6.522	4.048	0.924	13.776	9.317	9.32	12.77	9.988	7.36	9.395	6.8	32.342	50	MALE	1	163	54	20.32444	M0	N0	T3	1	Stage IIIA
TCGA-CC-A3M9	7.822	2.373	4.11	8.803	4.543	5.879	8.855	15.891	6.24	12.594	9.67	-0.211	16.634	9.107	10.916	5.789	3.036	6.28	6.822	-1.212	68.978	45	MALE	1	174	61	20.14797	M0	N0	T3	1	Stage IIIA
TCGA-CC-A3MA	7.64	10.932	1.541	5.143	8.128	10.154	12.032	16.476	8.014	8.569	5.652	2.318	14.863	9.134	8.982	13.545	12.832	11.896	11.287	7.613	28.867	61	MALE	1	168	66	23.38435	M0	N0	T3	1	Stage IIIA
TCGA-CC-A3MB	8.94	14.351	4.813	5.323	10.233	11.244	11.305	16.985	5.481	5.752	1.43	0.208	12.003	9.732	7.637	11.981	4.456	8.484	4.773	9.926	30.913	36	MALE	1	176	58	18.72417	M0	N0	T3	1	Stage IIIA
TCGA-CC-A3MC	8.079	12.402	4.587	5.134	12.894	10.406	11.445	18.134	7.362	9.421	3.002	-0.699	12.909	8.359	8.109	11.739	10.408	9.595	8.916	8.815	28.569	54	MALE	1	163	52	19.57168	M0	N0	T3	1	Stage IIIA
TCGA-CC-A5UC	10.613	17.265	9.615	8.486	11.183	10.906	9.927	16.734	6.849	8.259	3.21		13.948	8.606	7.795	10.923	7.751	8.018	6.243	8.646	33.748	63	MALE	1	165	57	20.93664	M0	N0	T3	1	Stage IIIA
TCGA-CC-A5UD	9.273	9.08	1.36	5.638	9.202	8.642	12.04	16.768	5.447	7.417	3.36	7.228	12.368	9.606	6.745	12.809	8.066	6.115	5.286	3.497	45.207	45	MALE	1	170	62	21.45329	M0	N0	T3	1	Stage IIIA
TCGA-CC-A5UE	11.599	15.165	5.021	6.525	10.017	12.169	11.482	16.547	4.299	6.582	-0.91		11.457	7.973	6.079	10.504	13.035	2.897	5.566	8.83	22.679	48	MALE	1	175	65	21.22449	M0	N0	T4	1	Stage IIIB
TCGA-CC-A7IF	9.714	13.549	9.345	5.438	13.9	14.045	11.436	15.927	5.745	8.486	2.726	0.556	12.675	8.219	6.784	12.201	5.643	8.189	6.85	8.363	31.309	59	MALE	1	169	58	20.30741	M0	N0	T3	1	Stage IIIA
TCGA-CC-A7IG	8.213	9.348	5.247	7.348	9.379	8.008	10.677	14.282	6.582	6.093	3.531	0.139	12.436	8.03	6.058	9.56	9.175	8.281	7.087	9.117	24.608	47	MALE	1	174	62	20.47827	M0	N0	T2	0	Stage II
TCGA-CC-A7IH	11.569	13.516	7.618	2.466	11.719	13.683	10.131	16.114	4.136	8.399	2.176	0.006	11.937	10.066	7.675	9.967	5.176	7.099	3.65	2.328	42.42	58	MALE	1	175	56	18.28571	M0	N0	T3	1	Stage IIIA
TCGA-CC-A7II	9.043	4.838	8.214	8.854	7.229	9.349	10.811	14.842	6.695	7.783	9.337	2.11	14.329	8.099	8.023	11.124	6.68	7.041	8.102	2.57	46.512	54	MALE	1	168	52	18.42404	M0	N0	T3	1	Stage IIIA
TCGA-CC-A7IJ	5.723		9.344	9.89	4.798	7.427	9.542	16.04	5.597	10.657	10.123	7.968	12.899	9.989	9.738	5.766	4.548	5.244	6.655	0.275	70.064	56	MALE	1	165	54	19.83471	M0	N0	T2	0	Stage II
TCGA-CC-A7IK	13.072	9.998	6.702	4.994	9.491	14.165	10.961	18.725	1.802	8.691	4.352	5.789	11.577	10.036	7.56	10.634	9.259	6.722	1.316	-1.006	52.567	59	MALE	1	168	57	20.19558	M0	N0	T3	1	Stage IIIA
TCGA-CC-A7IL	10.149	14.938	8.547	5.859	13.624	14.02	11.91	15.784	1.989	8.706	-0.011		10.917	8.139	5.118	11.747	3.076	9.941	3.381	5.138	29.269	61	MALE	1	174	63	20.80856	M0	N0	T3	1	Stage IIIA
TCGA-DD-A113	10.293	5.339	3.974	5.017	10.718	11.809	11.965	15.929	4.658	7.11	1.515	-0.07	12.543	9.126	7.913	13.468	10.041	10.437	4.389	8.818	23.533	55	FEMALE	0	154	56	23.61275	M0	N0	T2	0	Stage II
TCGA-DD-A114	9.556	11.436	4.118	6.416	9.639	9.695	10.962	17.141	6.943	7.993	3.621	1.118	14.215	9.702	9.405	11.481	11.666	8.995	6.22	10.604	32.134	42	MALE	1	173	74	24.72518	M0		T2	0	Stage II
TCGA-DD-A115	10.257	13.747	7.573	5.671	10.776	14.88	12.009	17.541	5.952	6.263	0.648	0.648	12.89	8.862	8.307	12.69	9.655	10.725	2.456	8.749	28.846	53	MALE	1	168	79	27.99036	M0	N0	T3	1	Stage IIIA
TCGA-DD-A116	12.137	13.712	7.668	4.775	11.581	15.973	10.316	16.107	4.427	9.705	1.383	8.532	12.688	8.834	6.74	8.059	4.668	7.284	5.809	4.383	48.53	68	MALE	1	175	73	23.83673	M0	N0	T3	1	Stage IIIA
TCGA-DD-A118	8.236	10.321	5.543	4.806	10.081	8.881	4.932	17.619	5.362	7.079	2.569	1.347	13.003	9.409	8.672	14.268	2.569	7.32	2.347	8.751	34.737	77	FEMALE	0	169	75	26.25958	M0	N0	T2	0	Stage II
TCGA-DD-A119	7.973	14.666	7.699	4.133	13.741	14.379	10.85	16.567	7.013	8.905	2.297	2.489	12.94	8.108	7.961	11.55	10.666	10.683	5.639	8.375	30.216	40	MALE	1	160	58	22.65625	M1	N0	T3a	1	Stage IV
TCGA-DD-A11A	11.692	14.919	10.918	6.192	12.046	14.052	11.747	16.777	4.87	6.927	0.063		12.257	8.986	7.455	12.328	10.337	8.804	5.472	6.222	25.917	67	MALE	1	156	67	27.53123	M0	N0	T1	0	Stage I
TCGA-DD-A11B	11.488	14.759	10.496	3.856	12.527	14.413	11.18	17.125	5.711	8.008	0.156	-0.844	11.41	9.146	7.404	11.171	2.326	9.345	1.741	7.664	37.049	73	MALE	1	181	134	40.90229	M0	N0	T1	0	Stage I
TCGA-DD-A11C	11.509	13.543	7.69	5.091	11.456	14.546	12.691	17.62	6.421	7.706	2.091	0.507	13.535	9.522	8.993	12.457	1.243	6.676	4.536	7.221	46.953	69	MALE	1	186	123	35.55324	M0	N0	T1	0	Stage I
TCGA-DD-A11D	10.771	14.437	8.996	5.839	13.567	13.121	11.74	17.754	5.488	5.073	1.167	1.167	12.329	8.799	7.736	13.366	4.414	10.037	2.166	10.885	30.386	57	FEMALE	0	163	73	27.47563	M0	N0	T1	0	Stage I
TCGA-DD-A1EA	9.342	12.064	4.83	2.186	11.084	13.887	10.825	17.385	3.356	9.311		-0.814	12.015	9.432	8.194	10.744	5.23	6.346	2.356	3.645	41.382	68	MALE	1	168	94	33.30499	M0	N0	T2	0	Stage II
TCGA-DD-A1EB	10.953		7.709	4.175	12.21	15.304	12.21	14.743	5.199	8.095	2.317	-0.683	12.425	9.536	8	12.971	3.84	6.236	2.317	6.975	39.503	72	FEMALE	0	164	57	21.19274	M0	N0	T1	0	Stage I
TCGA-DD-A1EC	10.44	2.768	3.003	6.173	8.862	9.087	9.362	15.917	4.851	7.561	2.681	-1.319	13.042	8.566	9.507	11.999	8.218	8.387	4.488	10.136	26.939	20	FEMALE	0	157	50	20.2848	M0	N0	T1	0	Stage I
TCGA-DD-A1ED	11.815	15.105	9.893	4.919	12.591	14.622	11.324	16.643	8.001	7.85	0.246	7.475	12.768	9.823	9.103	10.39	9.476	7.494	3.947	7.922	44.005	68	MALE	1	168	85	30.11621	M0	N0	T1	0	Stage I
TCGA-DD-A1EE	12.148	9.947	7.956	3.754	11.801	11.358	10.454	16.707	4.367	8.158	0.988		11.618	9.588	7.806	8.891	10.413	9.223	3.31	7.101	30.747	73	MALE	1	175	77	25.14286	M0	N0	T3	1	Stage IIIA
TCGA-DD-A1EF	11.016	14.493	7.735	5.435	11.267	10.024	8.973	15.658	4.735	7.234	3.056	1.735	12.554	9.331	8.396	13.594	3.735	10.321	2.735	7.978	33.31	57	FEMALE	0	164	81	30.116	M0	N0	T1	0	Stage I
TCGA-DD-A1EG	8.92	13.583	3.633	5.884	12.057	12.207	11.854	17.886	6.758	8.499	3.847	1.311	13.417	8.683	8.457	12.638	11.287	10.368	2.69	8.38	35.35	76	MALE	1	168	82	29.05329	M0	N0	T1	0	Stage I
TCGA-DD-A1EH	10.692	14.414	4.216	5.525	0.693	9.302	12.842	16.499	6.647	6.506	2.014	1.014	12.692	9.473	8.508	13.029	5.351	10.663	6.983	10.241	29.929	23	MALE	1	179	70	21.84701	M0	N0	T3	1	Stage III
TCGA-DD-A1EI	9.484	15.19	6.887	5.757	11.708	11.705	11.809	17.276	6.644	6.867	2.872	2.789	13.533	9.653	9.131	12.414	5.95	7.353	4.852	8.437	41.571	46	MALE	1	175	69	22.53061	M0	N0	T1	0	Stage I
TCGA-DD-A1EJ	10.41	16.978	6.467	7.063	10.73	9.728	11.544	16.541	3.772	5.148		-1.621	12.126	8.905	7.697	12.005	2.08	7.405	1.38	7.222	34.022	71	FEMALE	0	154	64	26.986	M0	N1	T1	0	Stage IIIC
TCGA-DD-A1EK	10.254	13.875	5.787	6.537	13.341	13.361	11.906	17.033	5.626	8.991			12.77	8.632	8.38	11.942	8	10.018	4.872	8.776	29.727	64	FEMALE	0	159	97	38.36874	M1	N0	T4	1	Stage IVB
TCGA-DD-A1EL	13.002	13.416	5.798	6.815	8.551	11.726	10.34	16.831	2.841	9.921	1.449	-0.551	11.727	9.284	7.818	8.842	9.212	7.034	0.449	3.493	40.627	23	MALE	1	182	54	16.30238	M0	N0	T2	0	Stage II
TCGA-DD-A39V	12.338	15.241	7.985	5.695	8.597	14.333	10.216	16.046	4.001	10.552	1.914		12.689	8.327	7.297	8.113	3.083	9.194	6.063	0.914	43.674	77	MALE	1	174	79	26.09328	M0	NX	T2	0	Stage II
TCGA-DD-A39W	13.503	15.931	10.363	5.762	10.057	12.589	11.102	17.264	4.54	10.555	0.839	6.754	11.27	9.751	7.266	10.435	0.254	7.042	2.062	3.647	55.9	29	FEMALE	0	177	74	23.62029	M0	N0	T3	1	Stage III
TCGA-DD-A39X	8.293	14.958	8.228	6.237	11.745	13.246	10.39	16.938	6.976	8.039		2.055	13.342	7.859	7.81	12.698	11.648	12.529	7.645	9.916	18.974	78	FEMALE	0	152	48	20.77562	M0	NX	T1	0	Stage I
TCGA-DD-A39Y	12.168	15.98	3.589	8.663	8.755	10.231	10.621	16.623	6.397	4.693	1.42	-0.165	11.623	7.245	6.21	11.531	10.777	9.831	7.446	9.84	15.239	67	MALE	1	176	85	27.4406	M0	NX	T1	0	Stage I
TCGA-DD-A39Z	8.835	17.049	8.55	4.081	10.626	12.452	11.37	15.966	3.64	8.118			11.958	7.838	5.691	9.972	3.292	9.125	4.003	9.878	28.311	43	FEMALE	0	165	61	22.40588	M0	NX	T2	0	Stage II
TCGA-DD-A3A1	9.094	17.208	2.13	3.004	11.221	12.835	11.117	18.001	4.902	5.902	-0.455		11.384	7.403	6.427	12.336	12.689	11.798	4.452	8.536	14.871	65	MALE	1	185	78	22.79036	M0	N0	T3b	1	Stage IIIA
TCGA-DD-A3A2	10.545	7.409	7.64	3.093	13.384	14.93	11.452	14.736	5.189	7.256	1.634	-0.366	11.181	8.841	7.695	12.003	3.541	4.158	0.634	7.634	39.648	76	FEMALE	0	178	85	26.82742	M0	N0	T1	0	Stage I
TCGA-DD-A3A3	9.315	16.269	7.399	4.469	13.072	13.661	9.695	16.576	2.884	6.749		4.325	12.289	6.585	5.503	11.277	5.503	11.567	8.749	8.781	18.73	45	MALE	1	167	71	25.45807	M0	N0	T1	0	Stage I
TCGA-DD-A3A4	11.836	8.114	8.666	3.462	12.14	13.668	9.786	16.304	3.114	7.747			12.809	9.032	7.089	13.036	3.561	6.632	3.742	9.439	29.472	37	MALE	1	189	65	18.19658	M0	N0	T3	1	Stage IIIA
TCGA-DD-A3A5	9.806	13.78	7.891	3.661	11.963	15.645	11.469	15.84	5.406	8.345	2.743		12.727	7.793	6.693	12.334	4.432	10.415	1.574	6.821	35.44	66	FEMALE	0	170	58	20.0692	M0	N0	T3	1	Stage III
TCGA-DD-A3A6	14.502	5.34	14.154	6.12	3.723	8.154	3.816	14.134	8.28	9.24	11.402	8.011	15.153	8.011	10.965	12.174	3.401	3.904	6.064	0.816	62.653	72	FEMALE	0	175	52	16.97959	M0	N0	T2	0	Stage II
TCGA-DD-A3A7	9.13	14.981	3.289	3.119	10.386	13.535	9.541	16.275	3.819	6.673	0.119	1.119	11.416	7.92	6.026	9.333	11.042	8.627	7.741	10.204	15.961	67	MALE	1	188	80	22.63468	M0	N0	T3b	1	Stage IIIB
TCGA-DD-A3A8	11.66	12.496	11.086	5.764	12.176	15.755	10.302	16.71	4.47	10.015	-0.484	8.181	12.088	9.093	6.023	9.011	1.516	8.367	3.101	7.152	47.25	75	MALE	1	177	99	31.60011	M0	N0	T2	0	Stage II
TCGA-DD-A3A9	7.154	14.852	6.641	6.061	5.709	10.918	10.579	16.962	7.732	7.59	4.046	3.161	14.184	8.774	7.569	10.968	3.853	7.9	5.392	8.742	43.744	64	FEMALE	0	168	62	21.96712	M1	N0	T4	1	Stage IVB
TCGA-DD-A4NA	5.609	3.3	3.777	4.236	3.236	5.152	13.249	15.149	5.571	7.232	5.531	1.777	13.436	10.096	9.015	14.107	7.555	4.391	13.831	10.311	30.861	67	FEMALE	0	166	93	33.74946	M0	N1	T2	0	Stage IIIC
TCGA-DD-A4NB	8.294	12.223	4.772	5.127	12.229	11.803	9.38	16.994	7.853	9.384	6.593	2.515	14.382	10.359	10.497	13.157	4.1	7.428	5.821	7.153	50.299	25	MALE	1	185	69	20.1607	M0	N0	T1	0	Stage I
TCGA-DD-A4ND	10.496	15.078	7.212	6.413	12.27	11.144	11.154	17.781	8.465	7.748	3.475	3.89	14.318	10.001	9.826	12.122	10.399	9.319	8.84	9.841	36.135	56	FEMALE	0	162	89	33.91251	M0	N0	T1	0	Stage I
TCGA-DD-A4NE	8.613	14.082	1.216	2.386	11.097	11.397	10.142	16.626	5.406	10.185	0.216	0.216	11.944	9.271	7.834	12.377	10.584	11.077	4.123	9.688	23.992	75	FEMALE	0	170	79	27.33564	M0	N0	T3a	1	Stage IIIA
TCGA-DD-A4NF	13.879	10.184	8.986	5.329	9.471	14.587	9.683	17.02	4.469	9.936	0.806	1.806	11.836	10.427	7.672	7.609	1.543	5.68	2.221	-0.779	57.382	72	MALE	1	173	86	28.73467	M0	N0	T1	0	Stage I
TCGA-DD-A4NG	11.251	13.923	4.955	3.618	12.433	12.077	11.577	17.601	3.54	5.252			12.187	9.559	8.187	10.791	1.955	8.264	6.042	8.138	32.712	77	MALE	1	164	94	34.94943	M0	NX	T3a	1	Stage IIIA
TCGA-DD-A4NH	9.867	14.228	7.379	6.575	11.131	7.698	11.007	16.717	6.869	7.388	3.108		12.599	8.973	8.595	12.381	4.78	10.404	8.81	9.106	29.776	65	FEMALE	0	157	81	32.86137	M0	N0	T3b	1	Stage IIIB
TCGA-DD-A4NI	10.926	15.464	9.532	4.506	13.504	14.45	11.228	17.445	7	7.171	-0.742	-0.742	12.942	9.546	8.913	10.375	6.553	10.364	4.428	7.053	33.988	67	MALE	1	172	99	33.46403	M0	NX	T2	0	Stage II
TCGA-DD-A4NJ	9.481	13.862	4.836	5.62	12.552	11.606	11.27	16.261	5.524	9.146	3.367	1.666	12.943	9.486	8.88	12.194	7.058	9.673	6.632	8.89	34.095	54	FEMALE	0	164	88	32.71862	M0	N0	T2	0	Stage II
TCGA-DD-A4NK	10.483	13.844	7.748	4.877	13.156	14.69	10.95	16.829	5.709	7.664	1.104		12.192	8.968	7.233	10.991	3.519	9.328	3.606	8.574	34.629	80	FEMALE	0		84		M0	N0	T3	1	Stage IIIA
TCGA-DD-A4NL	10.692	15.531	9.419	4.983	12.521	14.577	11.215	17.518	7.324	7.43	3.029	1.614	12.906	9.487	8.541	12.331	7.906	9.666	5.983	9.063	34.114	46	MALE	1	173	113	37.75602	M0	N0	T1	0	Stage I
TCGA-DD-A4NN	8.332	12.753	5.345	5.853	11.163	8.873	12.276	16.454	5.877	6.371	3.14	1.292	12.962	7.41	6.947	12.892	11.242	13.304	2.292	10.538	22.461	56	FEMALE	0	163	59	22.20633	M0	N0	T1	0	Stage I
TCGA-DD-A4NO	9.315	14.814	5.726	3.531	12.995	14.205	10.676	17.864	5.658	7.36	0.868	2.283	12.59	8.785	7.433	11.69	11.101	9.952	3.37	7.116	30.285	65	MALE	1	176	109	35.18853	M0	N0	T1	0	Stage I
TCGA-DD-A4NP	11.524	15.776	8.485	4.214	12.128	15.526	10.903	16.321	4.084	8.177	2.084	7.859	11.786	8.862	6.985	8.844	4.442	7.166	4.254	5.15	47.205	32	MALE	1	177	79	25.21625	M0	N0	T1	0	Stage I
TCGA-DD-A4NQ	11.644	14.294	5.084	6.897	9.966	10.912	10.225	18.109	7.969	6.291	2.606	0.799	12.359	8.564	8.152	10.477	11.99	9.008	5.886	8.204	29.507	60	MALE	1	159	75	29.66655	M0	N0	T2	0	Stage II
TCGA-DD-A4NR	9.292	16.098	6.815	8.617	10.86	11.236	10.011	17.412	6.234	7.137	3.022	2.285	14.275	8.081	9.588	12.813	12.162	11.694	8.673	10.238	22.464	85	FEMALE	0	64	54	131.8359	M0	N0	T1	0	Stage I
TCGA-DD-A4NS	11.037	12.919	7.341	5.57	11.353	13.59	11.103	16.847	8.275	8.082	4.538	3.57	14.45	9.906	10.184	11.903	10.235	10.26	11.204	8.882	34.472	61	FEMALE	0	167	58	20.79673	M0	N0	T1	0	Stage I
TCGA-DD-A4NV	10.855	13.748	9.103	5.017	12.967	13.651	11.116	16.193	6.726	6.028		1.536	12.96	9.128	8.993	12.33	6.111	9.939	2.411	5.141	36.749	61	MALE	1	179	129	40.26092	M0	N0	T3	1	Stage IIIA
TCGA-DD-A73A	10.429	15.621	7.752	5.561	12.169	13.396	11.604	18.129	4.909	10.867	-0.22		12.348	8.891	8.151	12.765	10.028	10.473	5.909	8.814	26.691	71	MALE	1	174	102	33.69005	M0	N0	T1	0	Stage I
TCGA-DD-A73B	10.568	16.065	7.205	4.711	13.093	14.651	10.363	16.604	3.418	9.177	-0.904		11.612	9.039	6.514	12.261	4.74	8.558	1.096	8.876	30.292	72	FEMALE	0	154	74	31.20256	M0	N0	T1	0	Stage I
TCGA-DD-A73C	11.24	3.171	8.722	5.414	12.891	15.284	11.751	16.245	4.394	8.097	1.586	1.586	13.033	10.091	8.722	12.462	6.404	4.788	2.849	4.879	44.124	65	FEMALE	0	159	49	19.38214	M0	N0	T3a	1	Stage IIIA
TCGA-DD-A73D	12.861	12.092	10.342	4.93	12.623	14.7	10.996	15.754	2.746	4.041			10.715	9.444	5.436	9.391	2.987	7.635	4.713	1.608	32.799	68	FEMALE	0	170	102	35.29412	MX	NX	T2	0	Stage II
TCGA-DD-A73E	14.294	9.352	8.075	2.856	11.226	14.625	11.281	17.046	3.8	6.756	0.741	3.478	11.219	9.454	6.582	9.246	4.8	8.008	3.326	6.326	38.653	66	MALE	1	172	108	36.50622	M0	N0	T1	0	Stage I
TCGA-DD-A73F	10.625	15.99	6.94	6.643	12.367	13.894	10.961	17.524	4.43	8.417	-0.614		13.21	8.825	8.741	12.841	4.193	10.582	3.386	7.73	32.762	77	FEMALE	0	164	69	25.65437	M0	N0	T1	0	Stage I
TCGA-DD-A73G	8.574	16.386	7.194	5.489	9.88	13.485	5.161	14.746	5.689	4.32	1.735	-0.266	12.536	6.617	5.982	12.116	5.161	10.588	5.056	8.859	14.741	73	FEMALE	0	155	44	18.31426	M0	N0	T1	0	Stage I
TCGA-ED-A459	9.049	14.785	1.773	5.916	11.809	10.822	10.361	16.371	6.059	7.857	1.773	-0.812	12.129	7.947	7.537	11.177	11.884	9.215	8.222	7.393	21.331	47	MALE	1	167	51	18.28678	M0	N0	T2	0	Stage II
TCGA-ED-A4XI	9.589	14.713	6.935	6.681	13.323	14.303	11.909	17.395	6.315	8.297	1.981	2.396	13.564	8.89	9.439	13.431	8.541	11.349	4.203	9.174	33.487	58	MALE	1	170	55	19.03114	M0	N0	T2	0	Stage II
TCGA-ED-A5KG	9.205	13.147	3.012	7.001	6.464	8.242	10.968	15.97	5.551	6.251	4.74	3.967	14.428	9.713	10.919	12.123	8.953	10.005	7.981	7.995	35.45	60	FEMALE	0	160	61	23.82813	M0	N0	T2	0	Stage II
TCGA-ED-A627	9.68	14.946	6.271	5.063	12.227	13.473	11.596	16.685	9.295	7.881	3.849	2.79	14.289	9.165	10.169	12.937	11.126	10.805	4.729	8.543	37.578	74	MALE	1	176	74	23.88946	M0	NX	T1	0	Stage I
TCGA-ED-A66X	10.915	14.024	9.622	7.863	11.409	11.285	8.383	16.91	7.306	7.68	4.838	2.302	14.173	8.691	9.498	13.141	11.647	12.242	6.819	10.152	25.78	35	MALE	1	170	61	21.10727	M0	N0	T3a	1	Stage IIIA
TCGA-ED-A66Y	7.13	14.724	2.521	5.153	9.472	10.899	12.228	16.57	4.409	8.797	2.521	2.369	11.819	9.725	7.442	12.625	3.007	1.521	1.521	10.557	46.649	51	FEMALE	0	160	59	23.04688	M0	N0	T3a	1	Stage IIIA
TCGA-ED-A7PY	8.413	15.123	8.031	2.778	11.42	12.501	11.365	16.512	5.015	6.218	2.778		11.585	6.218	5.585	13.034	8.702	12.524	5.14	9.88	15.995	20	FEMALE	0	156	40	16.43655	M0	NX	T2	0	Stage II
TCGA-ED-A7PZ	12.048	11.694	5.478	6.487	8.865	14.996	9.21	15.013	4.753	4.387	2.309		12.593	7.602	6.139	12.33	10.331	10.445	3.598	7.433	17.869	61	MALE	1	159	50	19.7777	M0	NX	T2	0	Stage II
TCGA-EP-A12J	9.569	11.204	8.136	2.642	11.255	14.296	11.03	17.755	3.883	11.073	0.767	-0.818	11.485	9.558	7.608	9.206	5.248	9.791	2.642	8.627	36.828	62	MALE	1	173			MX	NX	T1	0	Stage I
TCGA-EP-A26S	11.193	12.25	6.904	4.517	11.387	14.141	10.676	16.667	5.371	8.989	1.139		12.033	9.232	7.886	9.612	3.861	9.657	3.517	6.531	38.814	70	MALE	1	168	41	14.52664	MX	N0	T1	0	Stage I
TCGA-EP-A2KA	8.189	16.187	1.831	5.568	4.376	10.063	11.056	16.352	4.861	8.837	3.568	0.246	12.196	8.168	7.098	12.479	5.905	9.118	10.921	10.997	22.963	52	FEMALE	0	178			MX	NX	T3a	1	Stage IIIA
TCGA-EP-A2KB	10.767	15.981	7.84	6.095	10.653	12.464	11.967	16.185	4.437	6.581	2.53		12.135	9.171	6.32	12.303	1.267	7.149	1.752	6.624	40.23	46	FEMALE	0	165	81	29.75207	MX	NX	T1	0	Stage I
TCGA-EP-A2KC	11.523	13.721	8.101	4.826	12.166	13.083	11.337	16.5	4.456	8.787	1.683	0.099	12.319	9.032	7.634	9.799	9.193	8.728	4.525	5.42	34.183	62	MALE	1	171			MX	NX	T1	0	Stage I
TCGA-EP-A3JL	9.485	14.823	5.722	6.163	12.461	13.358	10.785	16.331	6.413	9.209	2.593	1.593	13.397	8.17	8.479	11.92	11.271	11.131	4.237	7.874	30.538	76	MALE	1	168	73	25.86451	MX	NX	T1	0	Stage I
TCGA-EP-A3RK	12.444	14.452	8.061	7.142	11.35	12.22	9.834	17.018	5.21	8.466	1.995	0.188	13.101	8.35	8.813	8.046	6.368	8.417	5.491	3.51	41.145	73	MALE	1	178	101	31.87729	MX	NX	T3a	1	Stage IIIA
TCGA-ES-A2HS	9.91	17.632	9.358	4.426	13.547	16.316	10.951	15.963	5.759	7.557	1.782	1.367	12.299	8.011	7.021	11.41	3.689	8.44	3.104	6.29	37.777	80	MALE	1	180	97	29.93827	MX	NX	T1	0	Stage I
TCGA-ES-A2HT	7.173	16.427	6.281	3.769	13.884	14.767	9.682	16.042	6.929	6.769	1.682	2.489	13.185	7.527	7.333	10.348	4.003	12.728	4.891	10.112	29.555	54	MALE	1	174	80	26.42357	MX	NX	T1	0	Stage I
TCGA-FV-A23B	10.034	16.183	5.137	4.957	8.094	12.994	12.081	17.751	3.625	6.638	1.455	-1.13	12.644	9.397	8.313	12.187	2.777	4.625	1.87	4.079	45.235	70	FEMALE	0				M0	N0	T2	0	Stage II
TCGA-FV-A2QQ	10.101	15.813	8.063	5.369	13.359	14.092	10.821	16.651	6.38	9.442	0.325	2.325	12.807	8.122	7.678	12.456	10.738	11.154	2.647	9.431	28.126	80	MALE	1				MX	N1	T1	0	Stage IVA
TCGA-FV-A2QR	9.221	12.887	5.645	5.662	12.32	12.518	10.871	16.714	6.286	6.889	3.253	-0.747	12.894	9.29	8.095	12.052	7.725	10.909	1.253	9.1	32.508	75	MALE	1				M0	N0	T1	0	Stage I
TCGA-FV-A3I0	6.026	5.987	1.232	4.554	10.237	6.227	7.169	15.027	7.121	6.883	2.932	3.987	14.552	8.209	7.616	14.033	13.275	12.482	7.226	8.759	17.719	76	FEMALE	0				M0	NX	T2	0	Stage II
TCGA-FV-A3I1	10.824	13.78	3.439	6.024	12.533	12.111	10.751	17.962	5.625	6.768	3.204	2.702	12.764	8.828	8.516	12.476	12.652	10.056	2.817	8.267	30.851	81	FEMALE	0				MX	N0	T2	0	Stage II
TCGA-FV-A3R2	9.421	13.782	-1.203	3.321	12.609	13.634	11.346	17.088	3.321	8.532	1.119		11.707	8.749	7.269	9.876	6.036	8.629	0.797	9.056	34.737	75	MALE	1		75		MX	NX	T1	0	Stage I
TCGA-FV-A3R3	9.166	12.985	8.465	5.305	10.297	11.156	9.887	17.507	8.054	9.425	2.196	1.781	13.96	10.136	9.451	14.165	8.958	10.864	1.518	8.909	37.983	38	FEMALE	0	161	97	37.4214	MX	NX	T1	0	Stage I
TCGA-FV-A495	8.797	15.246	8.342	5.437	11.687	14.796	10.986	15.749	6.678	8.598	2.463	1.24	13.621	7.306	7.397	11.259	8.973	10.536	5.655	7.82	29.794	51	FEMALE	0	172	70	23.66144	M0	NX	T2	0	Stage II
TCGA-FV-A496	9.002	13.642	5.451	3.756	10.796	14.373	11.761	16.306	2.949	5.949			11.668	9.082	6.197	12.901	5.649	9.514	4.979	7.899	22.969	84	FEMALE	0	162	54	20.57613	M0	NX	T1	0	Stage I
TCGA-FV-A4ZP	10.915	17.329	8.978	4.444	8.409	11.194	10.939	15.564	4.813	2.444	1.859	2.666	12.126	7.116	6.717	10.735	12.229	10.422	11.02	9.698	10.138	78	MALE	1	168	69	24.44728	M0	NX	T3	1	Stage IIIA
TCGA-FV-A4ZQ	12.046	14.395	-0.937	8.248	10.604	11.562	10.748	16.054	2.385	9.41	0.063	4.555	12.007	9.072	8.604	11.528	3.763	7.889	3.063	7.763	38.891	52	MALE	1				M0	NX	T1	0	Stage I
TCGA-G3-A25S	11.355	12.277	8.749	5.344	11.6	13.466	10.198	14.585	1.82	7.516	-0.764		11.304	7.98	6.118	10.158	10.724	9.528	6.445	10.65	11.254	64	MALE	1	166	73	26.49151	M0	N0	T1	0	Stage I
TCGA-G3-A25T	6.417	12.149	3.924	3.187	10.376	10.345	9.575	15.786	6.866	8.38	5.17	3.602	13.913	7.38	7.221	13.664	13.143	12.666	8.189	9.49	20.741	45	FEMALE	0	167	64	22.94812	M0	N0	T3	1	Stage IIIA
TCGA-G3-A25U	12.342	14.841	11.845	5.01	14.182	14.614	11.468	17.135	5.036	8.293	0.203		11.192	9.217	6.917	13.022	5.507	9.044	1.788	7.718	32.382	63	FEMALE	0	153	47	20.07775	M0	N0	T1	0	Stage I
TCGA-G3-A25V	10.381	15.564	8.091	5.622	13.2	13.726	11.407	17.415	7.404	7.582	1.754	2.602	13.098	8.875	8.706	12.396	7.94	9.207	5.455	9.092	34.753	68	MALE	1	187	90	25.73708	M0	N0	T1	0	Stage I
TCGA-G3-A25X	8.474	12.358	6.449	6.712	5.195	8.189	11.606	16.493	6.217	6.728	3.697	2.495	14.017	8.338	7.145	10.576	8.348	9.967	13.325	8.009	26.51	73	MALE	1	165	65	23.87511	M0	N0	T2	0	Stage II
TCGA-G3-A25Y	9.098	12.666	7.872	5.787	11.4	7.636	11.545	16.327	8.342	6.898	3.145	2.309	13.348	8.914	8.279	12.748	4.691	10.706	7.651	9.341	33.97	52	FEMALE	0	158	57	22.83288	M0	N0	T1	0	Stage I
TCGA-G3-A25Z	9.359	16.143	6.503	4.866	12.756	13.805	11.212	17.297	6.088	7.74	1.918		12.934	8.377	7.281	12.041	3.866	10.186	2.57	8.166	36.02	58	MALE	1	173	69	23.05456	M0	N0	T1	0	Stage I
TCGA-G3-A3CG	9.111	14.31	6.746	3.939	12.483	12.65	10.912	16.776	6.817	5.865	1.617	1.939	12.426	8.88	7.583	12.118	1.617	6.855	4.902	8.109	39.212	80	MALE	1	168	93	32.95068	M0	N0	T1	0	Stage I
TCGA-G3-A3CH	9.863	14.649	9.252	5.752	13.063	14.632	11.557	16.713	6.777	9.295	1.342	1.342	12.853	9.283	8.505	11.467	5.015	8.576	5.043	8.66	38.907	53	MALE	1	178	74	23.35564	M0	N0	T3a	1	Stage IIIA
TCGA-G3-A3CI	12.088	14.779	11.362	4.868	13.23	13.971	11.024	14.811	7.319	8.787	2.675	1.453	12.647	9.109	8.599	12.931	3.453	7.934	4.038	5.26	42.807	71	MALE	1	167	98	35.1393	M0	N0	T1	0	Stage I
TCGA-G3-A3CJ	11.401	15.514	10.472	6.297	12.486	14.131	10.708	14.825	5.037	8.372	1.932	-0.389	12.544	8.328	6.517	3.698	1.195	8.041	1.932	6.07	46.938	52	MALE	1	174	72	23.78121	M0	N0	T2	0	Stage II
TCGA-G3-A3CK	12.878	14.126	9.216	3.273	12.223	13.806	10.993	17.141	4.206	8.734	1.621	-0.186	11.432	9.395	7.979	10.67	7.167	7.113	2.721	4.569	39.077	61	MALE	1	170	67	23.18339	M0	N0	T1	0	Stage I
TCGA-G3-A5SI	10.253	13.405	6.793	2.311	13.346	13.35	10.592	15.873	5.584	5.896	1.311		12.074	8.296	6.584	12.811	4.37	10.807	4.427	7.514	26.284	44	MALE	1	177	74	23.62029	M0	N0	T2	0	Stage II
TCGA-G3-A5SJ	9.248	15.127	1.917	4.855	10.497	9.83	11.78	17.984	6.263	8.28	4.855	-0.668	13.071	9.228	8.043	13.047	11.862	10.577	8.474	10.141	24.735	59	MALE	1	171	87	29.75274	M0	NX	T1	0	Stage I
TCGA-G3-A5SK	10.415	15.875	7.795	4.074	14.453	14.63	11.679	17.446	5.466	6.264	1.074		11.721	8.521	8.409	13.211	3.396	9.935	1.074	7.051	35.914	58	MALE	1	169	67	23.45856	M0	NX	T1	0	Stage I
TCGA-G3-A5SL	11.704	14.091	9.888	3.975	13.492	14.823	10.661	16.844	5.098	11.42	1.752	6.56	12.401	9.561	7.542	11.423	3.255	7.471	3.811	3.868	52.011	70	MALE	1	180	88	27.16049	M0	NX	T2	0	Stage II
TCGA-G3-A5SM	8.851	13.858	5.679	5.509	12.325	14.101	10.34	16.461	6.969	8.094	2.75	2.05	13.498	9.511	8.875	12.576	8.928	7.558	5.542	5.708	38.237	58	MALE	1	184	106	31.30907	M0	NX	T2	0	Stage II
TCGA-G3-A6UC	10.289	12.545	7.591	3.609	13.689	15.011	11.217	16.565	2.747	5.145	0.872		11.583	9.197	6.787	11.869	6.364	10.091	5.917	4.747	25.124	65	MALE	1	182	91	27.47253	M0	N0	T3b	1	Stage IIIB
TCGA-G3-A7M5	9.597	15.24	10.037	4.784	13.063	15.557	10.531	17.028	6.58	5.497	1.199		11.602	7.834	5.954	12.34	4.138	11.459	3.074	5.9	29.314	76	MALE	1	164	68	25.28257	MX	NX	T1	0	Stage I
TCGA-G3-A7M6	8.198	12.335	6.702	6.823	9.221	11.936	12.534	15.677	8.146	6.634	2.369	0.668	13.781	8.808	8.113	13.181	10.793	9.562	5.634	8.122	29.439	60	FEMALE	0	158	77	30.84442	MX	NX	T1	0	Stage I
TCGA-G3-A7M8	12.522	15.217	10.266	6.132	13.187	15.321	9.903	16.155	8.259	6.983	1.193	-0.392	12.978	8.961	8.252	13.379	6.281	8.884	1.608	7.685	34.456	31	MALE	1				MX	NX	T1	0	Stage I
TCGA-GJ-A6C0	8.789	14.377	1.921	6.8	12.02	10.922	10.673	17.494	6.158	9.596	1.698	-0.887	12.982	8.713	8.035	12.498	4.668	8.248	4.573	9.045	35.43	75	FEMALE	0	160	90	35.15625	MX	NX	T2	0	Stage II
TCGA-HP-A5MZ	10.759	15.81	9.361	5.941	12.882	12.926	10.532	17.229	7.49	9.634	4.604	3.443	14.039	9.765	9.592	11.725	8.344	10.836	4.91	8.451	42.061	78	MALE	1	185	82	23.95909	MX	NX	T1	0	Stage I
TCGA-HP-A5N0	11.052	15.427	10.903	7.095	13.173	14.821	11.53	16.745	6.002	7.677	3.088	2.557	13.837	9.824	9.504	12.676	11.78	10.187	1.709	7.758	36.655	90	FEMALE	0	156	65	26.7094	M0	NX	TX		
TCGA-K7-A5RF	10.864	16.53	8.104	6.009	13.539	14.754	11.432	16.518	8.371	7.493	3.317	2.616	13.373	8.906	9.497	12.947	9.511	9.981	9.833	9.294	29.956	64	MALE	1	177	75	23.93948	MX	NX	T1	0	Stage I
TCGA-K7-A5RG	10.272	12.794	6.994	7.33	9.989	12.686	11.809	17.443	5.876	7.434	2.739	1.417	13.212	9.23	9.725	13.424	11.684	8.989	5.684	7.66	31.446	66	MALE	1	173	123	41.09726	MX	NX	T1	0	Stage I
TCGA-K7-A6G5	9.366	16.217	7.283	5.408	13.111	14.023	11.25	16.328	6.73	7.877	0.502	2.671	12.557	8.017	7.27	12.18	6.631	11.761	2.671	9.665	30.293	66	MALE	1	184	80	23.62949	MX	N0	T1	0	Stage I
TCGA-KR-A7K0	12.943	13.469	5.834	5.626	12.466	13.97	11.178	17.961	6.334	9.275	1.798	5.936	12.825	9.518	8.091	11.012	10.135	7.51	3.284	5.952	45.023	65	MALE	1	174	66	21.79945	M0	N0	T1	0	Stage I
TCGA-KR-A7K2	8.735	13.986	6.02	3.612	11.143	14.674	10.206	16.92	6.706	7.031	2.234	1.497	13.177	8.497	7.526	13.798	10.245	13.096	6.482	10.526	19.647	64	MALE	1	166	79	28.66889	M0	N0	T1	0	Stage I
TCGA-KR-A7K7	12.459	17.072	6.599	5.66	11.223	13.093	9.563	16.629	4.277	7.684	1.47	-0.53	11.666	6.888	6.412	7.547	11.676	9.053	5.993	9.404	20.386	61	FEMALE	0	168	125	44.28855	M0	N0	T2	0	Stage II
TCGA-KR-A7K8	11.258	15.145	9.298	7.033	12.388	12.481	11.614	15.85	6.27	7.418	3.449	0.542	14.007	8.178	8.225	12.163	3.127	9.242	5.808	5.001	40.21	57	MALE	1	170	109	37.71626	M0	N0	T1	0	Stage I
TCGA-LG-A6GG	9.126	15.011	5.102	4.259	10.934	14.122	11.454	17.524	3.695	7.017	0.756		12.066	8.349	7.188	11.141	4.756	9.341	4.126	8.572	30.112	79	FEMALE	0	158	65	26.03749	M0	NX	T2	0	Stage II
TCGA-MI-A75C	8.251	15.308	4.44	5.394	12.407	12.658	10.393	16.391	3.44	7.716		-0.56	11.616	7.127	5.798	11.433	11.228	10.641	5.726	8.822	14.073	64	MALE	1	175	84	27.42857	M0	N0	T1	0	Stage I
TCGA-MI-A75E	9.742	15.717	5.958	5.135	12.864	13.687	11.386	17.691	7.248	7.654	2.435	2.02	13.514	8.261	7.761	12.012	10.099	9.941	4.989	8.213	32.715	61	MALE	1	175	103	33.63265	M0	N0	T4	1	Stage IIIC
TCGA-MI-A75G	10.263	16.521	6.922	4.864	13.314	14.931	11.244	16.764	5.057	7.602	1.249	-0.751	11.971	8.373	6.082	11.764	6.095	9.859	1.571	8.698	29.604	63	MALE	1	178	69	21.77755	M0	N0	T2	0	Stage II
TCGA-MI-A75H	12.214	12.579	8.246	6.228	11.402	15.789	10.405	16.037	3.73	10.982	0.643	0.643	13.014	9.294	7.983	10.836	1.965	8.235	2.813	3.102	45.779	77	MALE	1	173	84	28.06642	MX	NX			
TCGA-MI-A75I	10.134	12.842	6.057	6.472	12.086	12.734	11.686	16.324	3.55	7.898	0.887	0.302	11.987	8.553	6.975	11	7.536	8.239	4.55	8.259	28.577	61	MALE	1	188	100	28.29335	MX	NX	T2	0	
TCGA-MR-A520	11.109	14.701	9.932	6.294	13.272	14.443	11.108	15.998	6.133	9.144	1.309	0.724	11.968	8.724	7.601	12.6	3.631	6.884	2.046	5.15	42.399											
TCGA-NI-A4U2	11.236	6.238	9.411	6.549	14.457	15.485	11.784	15.859	5.572	7.07	1.402		13.36	9.086	8.128	12.28	4.118	3.549	4.744	2.916	44.652	71	MALE	1	171	82	28.04282	MX	NX	T3	1	Stage IIIA
TCGA-O8-A75V	9.779	15.731	7.331	6.629	13.338	13.534	11.241	16.815	7.196	8.333	2.061	1.254	13.091	7.951	7.629	12.248	10.815	10.549	5.463	9.547	26.949	54	MALE	1	180	92	28.39506	MX	NX	T1	0	Stage I
TCGA-PD-A5DF	9.779	13.618	7.658	6.72	13.141	12.789	10.822	17.304	7.318	8.259	4.143	2.236	13.98	9.003	8.808	12.615	10.45	11.083	3.406	7.161	37.157	58	FEMALE	0	167	72	25.81663	MX	NX	T4	1	Stage IIIB
TCGA-QA-A7B7	8.861	13.414	2.909	4.762	10.899	11.114	9.264	17.133	2.987	5.369	1.739	-0.261	11.851	8.88	6.664	10.315	3.263	8.33	4.132	2.646	34.941	48	MALE	1	180	108	33.33333	MX	NX	T2	0	Stage II
TCGA-RC-A6M3	10.249	6.294		7.938	9.317	9.566	9.843	15.365	9.815	5.542	3.464	4.995	11.306	8.787	7.056	11.539	11.34	10.192	10.957	8.804	23.342											
TCGA-RC-A6M4	12.501	13.507	5.876	1.929	11.737	15.05	10.828	16.26	2.706	7.543			11.005	8.662	5.548	8.964	2.706	7.664	5.028	7.479	30.711											
TCGA-RC-A6M5	10.071	12.111	7.629	5.942	11.795	11.705	6.229	16.291	7.587	9.303	3.874	4.697	14.694	9.834	9.381	13.739	4.846	8.633	5.383	9.621	39.669											
TCGA-RC-A6M6	11.87	15.45	6.793	7.98	10.754	8.414	11.159	17.927	6.347	4.986	1.935	0.128	12.795	7.28	6.288	13.056	10.689	10.812	8.9	8.614	16.776											
TCGA-RC-A7S9	9.615	13.128	4.879	5.788	12.889	11.316	10.616	16.563	4.586	7.046	0.264		11.886	7.699	6.604	11.629	9.465	10.302	5.195	10.547	18.125	47	FEMALE	0	161	58	22.37568	M0	N0	T1	0	Stage I
TCGA-RG-A7D4	10.186	15.864	4.605	6.892	10.75	11.495	11.561	16.572	3.648	7.883	2.368		12.35	8.419	7.637	9.945	8.927	7.115	4.084	6.97	33.395	69	MALE	1	172	87	29.40779	M0	N0	T2	0	Stage II
TCGA-T1-A6J8	7.256	13.631	-0.75	3.337	12.736	14.353	11.317	16.878	5.4	7.405	-0.75	1.572	11.971	8.427	7.558	12.131	11.492	10.513	4.774	7.3	23.568	68	MALE	1	188	65	18.39067	M0	NX	T1	0	
TCGA-UB-A7MA	5.41	12.654	2.008	2.786	7.35	8.67	11.463	17.019	5.558	4.523	0.201	2.008	13.713	9.421	7.386	12.656	8.65	12.28	8.584	9.531	19.59	62	FEMALE	0	165	85	31.2213	M0	N0	T2b	0	Stage II
TCGA-UB-A7MB	10.982	14.502	9.982	5.534	13.136	12.457	11.17	16.937	3.153	10.459	-0.095		11.273	8.823	6.527	12.439	2.365	9.807	4.19	6.888	32.558	24	MALE	1	180	62	19.1358	MX	NX	T2	0	Stage II
TCGA-UB-A7MC	11.802	7.966	6.668	4.784	12.304	13.263	10.745	18.359	4.089	7.208	1.019	-1.303	11.525	9.777	6.935	11.499	9.685	9.466	3.826	2.697	31.181	59	MALE	1	176	78	25.18079	MX	N0	T3a	1	Stage IIIA
TCGA-UB-A7ME	8.237	13.423	5.03	5.708	11.264	12.803	12.362	16.227	6.524	6.094	3.833	1.57	13.271	7.746	8.036	14.3	12.357	11.278	6.428	10.205	21.097	51	MALE	1	165	75	27.54821	MX	NX	T1	0	Stage I
TCGA-UB-A7MF	9.671	14.178	4.942	6.312	10.543	10.183	11.441	16.094	4.475	3.505	2.09	-1.08	12.422	8.482	7.614	12.465	10.022	9.137	7.753	9.372	16.293	56	MALE	1	195	95	24.98356	MX	NX	T3a	1	Stage IIIA

Supplementary Table 2

EMT Score	CD36	FABP1	FABP4	FABP5	SLC27A2	SLC27A5	BMI	BMI_Log2
Pearson correlation	0.271	-0.133	0.346	0.178	-0.049	0.157	0.015	0.050
P-value (two-tailed)	0.000	0.068	0.000	0.014	0.499	0.030	0.862	0.552

BMI_Log2	EMT SCR	CD36	FABP1	FABP4	FABP5	SLC27A2	SLC27A5	BMI
Pearson correlation	0.050	0.262	0.002	0.091	0.118	0.065	-0.006	0.910
P-value (two-tailed)	0.552	0.002	0.985	0.281	0.161	0.444	0.940	0.000

BMI_Log2	CDH2	FN1	SNAI1	SNAI2	TWIST1	TWIST2	VIM	ZEB1	ZEB2	CDH1	CLDN4	CLDN7	MUC1	TJP3	EMT SCR	BMI
Pearson correlation	0.034	0.230	0.067	-0.072	-0.104	-0.075	-0.042	0.174	0.082	-0.125	0.061	-0.018	-0.005	-0.105	0.050	0.910
P-value (two-tailed)	0.687	0.006	0.429	0.394	0.235	0.441	0.615	0.037	0.328	0.137	0.473	0.827	0.952	0.210	0.552	0.000

Bonferroni corrected p-value 0.0016

Supplementary Table 3

Patient clinical information								Western Blot quantification								
TSS ID	BMI	SEX	Grade	T	N	M	STAGE	CD36	VIM	ZEB1	ZEB2	TGFB	PORCN	CDH	DSP	SNAIL
Patient Key:6680	16.28	M	3	2	0	0	II	1.18	0.64	0.67	0.74	0.39	0.24	0.34	0.34	0.38
Patient Key: 3993	19.89	F	2	3	0	0	III	1.81	1.72	1.18	1.26	0.46	2.59	0.88	0.20	0.24
Patient Key:2604	21.6	F	2	1	0	0	I	1.00	1.77	0.42	0.52	0.68	2.11	0.34	0.13	0.05
Patient Key:5828	21.75	M	3	3	0	0	III	0.09	0.63	0.01	0.02	0.30	1.08	0.07	0.08	0.00
Patient Key:3167	22.77	M	3	3A	0	1	IV	1.51	0.57	0.66	0.74	0.28	1.10	0.40	0.28	0.02
Patient Key:5858	22.82	M	2	1	0	0	I	0.08	0.13	0.01	0.01	0.13	0.09	0.03	0.24	0.04
Patient Key:2613	23.63	F	3	2	0	0	II	0.01	0.09	0.00	0.01	0.32	0.06	0.01	0.00	0.00
Patient Key:3013	23.77	M	3	3	0	0	IIIA	0.60	0.39	0.33	0.23	0.36	1.49	0.27	0.09	0.00
Patient Key: SW1	36.05	F	2	3	0	0	IIIA	1.64	2.24	0.69	0.68	0.86	2.17	0.73	0.11	0.76
Patient Key: 8118	35.14	M	2	3A	X	0	X	1.26	0.93	0.73	0.79	0.84	1.03	0.58	0.04	0.30
Patient Key: 10316	35.45	F	1	2	X	X	X	2.28	2.27	1.27	1.04	0.50	2.33	0.75	0.67	0.00
Patient Key:4068	35.49	M	3	1	0	0	I	1.15	0.91	0.69	0.82	0.61	1.54	0.68	0.05	0.14
Patient Key:3423	37.66	M	2	1	0	0	I	0.85	2.76	0.20	1.12	0.52	3.37	0.58	0.09	0.12
Patient Key: SW14	39.97	M	1	3	0	0	IIIA	1.56	1.49	0.70	0.16	0.76	1.40	0.21	0.00	1.63
Patient Key: 6796	35.32	F	3	2	1	0	IIIC	0.01	0.68	0.00	0.00	0.72	1.10	0.02	0.00	0.31
Patient Key: 3070	36.04	M	3	3	0	X	X	1.03	1.21	0.54	0.29	0.86	0.91	0.19	0.06	0.25

**TSS ID**	**Hx of disease**
Patient Key:6680	chronic HBV; dx with HCC 6/2/2009; HCC recurrence on 12/3/2009;
Patient Key: 3993	dx with HCC 7/23/1996; recurrence on 10/15/1996
Patient Key:2604	extreme smoking until 1996, dx with HCC w/o Cirrhosis 5/7/2001, recurrence 10/1/2002, recurrence 05/14/2003
Patient Key:5828	Chronic HBV; dx with HCC 05/29/2008; Metastatic HCC to lung 4/8/2009
Patient Key:3167	Chronic HBV; dx with HCC 4/8/2003;
Patient Key:5858	Chronic HBV: dx with cirrhosis in 2000, dx with HCC 6/13/2008;
Patient Key:2613	Smoking history for 45 yrs; occasional alcohol consumption; dx with HCC 2006; recurrence of HCC 2010;
Patient Key:3013	Chronic HCV; dx with HCC 1/18/2002; Metastatic HCC to sternum 10/19/2004
Patient Key: SW1	dx with HCC 2/9/2004
Patient Key: 8118	remote alcohol history; dx with HCC 1/10/2011
Patient Key: 10316	Chronic HCV; advance fibrosis; dx with HCC in 2012; reccurent HCC 5/6/2014; HCC with Cirrhosis 5/7/2014
Patient Key:4068	dx with HCC 2/27/2006
Patient Key:3423	nonalcoholic steatohepatitis with cirrhosis; dx with HCC 5/31/2005
Patient Key: SW14	dx with HCC 2007
Patient Key: 6796	dx with CCA 6/11/2009 then determined to be HCC 6/27/2009;
Patient Key: 3070	Heavy alcohol use; dx with HCC 6/25/2002;

**TSS ID**	**Treatment history**
Patient Key:6680	resection in 6/22/2009;
Patient Key: 3993	resection in 7/23/1996; alcohol ablation in 10/17/1996; Chemoembolization 4/9/1997
Patient Key:2604	hepatectomy in 5/14/2001; alcohol ablation 10/2/2002; alcohol ablation 05/15/2003
Patient Key:5828	embolization 6/4/2008; treated with entecavir for HBV 5/52/2008; right hepatectomy & left portal vein thrombectomy 7/1/2008; Radiofrequency ablation 11/4/2008; Sorafenib 6/24/2009, Sorafenib+doxorubicin 1/5/2010, Sorafenib only 6/22/2010; MC0812 Chemotherapy (Cisplatin, gemcitabine, and everolimus) 11/11/2010; Cisplatin & Gemcitabine only 3/3/2011; Pazopanib & everolimus 7/24/2012
Patient Key:3167	embolization right portal vein 4/14/2003; Hepatectomy, right adrenalectomy, & cholecystectomy 5/6/2003
Patient Key:5858	portal embolization on the right hepatic lobe 6/17/2008
Patient Key:2613	resection 2006 & 2010; laser ablation 10/14/2011, 6/7/2012, 9/20/2012, 10/4/2012, & 7/24/2013; Radiofrequency 10/28/2013; MR guided liver cyroablation 6/25/2014; MR cyroablation 1/7/2015;
Patient Key:3013	Lamivudine 1/18/2002; resection 1/21/2002; Epivir 5/28/2002;
Patient Key: SW1	right hepatectomy 2/26/2004; TheraSphere treatment 8/31/2006; Hospice care 3/21/2007
Patient Key: 8118	resection 1/14/2011;
Patient Key: 10316	resection 9/2012; ethanol ablation 5/13/2014
Patient Key:4068	right hepatectomy 3/3/2006
Patient Key:3423	resection 6/7/2005
Patient Key: SW14	resection 6/2007
Patient Key: 6796	resection 6/24/2009
Patient Key: 3070	resection 7/8/2002

Supplementary Table 4

HepG2	PA vs. BSA fold-change	Transcriptional regulation by TGF-β	Transcriptional regulation by WNT/β-catenin	Hep3B	PA vs. BSA fold-change	Transcriptional regulation by TGF-β	Transcriptional regulation by WNT/β-catenin
AHNAK	14.091	x		IL1RN	3.024		
COL5A2	3.672	x		MST1R	2.335		
WNT5B	3.630	x		TGFB2	2.194	x	
WNT11	3.095			SNAI3	2.174		
BMP2	2.745			TGFB3	2.033		
IL1RN	2.567			IGFBP4	1.995	x	
PPPDE2	2.555			ZEB2	1.858		
NODAL	2.520			BMP7	1.741		
FOXC2	2.479	x		NUDT13	1.617		
SOX10	2.229			SMAD2	1.576		
TSPAN13	2.204			TCF4	1.569	x	x
STEAP1	2.056			RGS2	1.562		
TGFB2	1.973	x		CDH2	1.558	x	
CDH1	1.950	x		COL3A1	1.555	x	
CALD1	1.749	x		SPP1	1.551		
MST1R	1.741			ESR1	1.548	x	
WNT5A	1.741	x		CALD1	1.491	x	
KRT7	1.709			CTNNB1	1.481	x	
VIM	1.606			GSK3B	1.468	x	
COL3A1	1.591	x		ILK	1.468		
MSN	1.569			TSPAN13	1.468		
ZEB2	1.562			PTP4A1	1.464		
MMP9	1.544			FGFBP1	1.447		
KRT19	1.488			VCAN	1.431		
SPARC	1.488			GNG11	1.418		
MMP3	1.485			GSC	1.418		
BMP7	1.478			TWIST1	1.418		
COL1A2	1.478			MAP1B	1.404		
GNG11	1.478			ZEB1	1.404		
GSC	1.478			PDGFRB	1.376		
TWIST1	1.478			SIP1	1.363		
PDGFRB	1.451			BMP1	1.357		
MMP2	1.414			F11R	1.357		
IGFBP4	1.408			EGFR	1.347		
VCAN	1.395			ITGAV	1.338		
TMEM132	1.382			PTK2	1.335		
OCLN	1.323			OCLN	1.326		
TFPI2	1.320			COL1A2	1.320		
SNAI3	1.310			COL5A2	1.281		
TCF4	1.301			KRT19	1.281		
MAP1B	1.298			KRT14	1.278		
ILK	1.295			TIMP1	1.275		
PTK2	1.260			SOX10	1.269		
RGS2	1.260			ITGA5	1.263		
NUDT13	1.251			PPPDE2	1.260		
FGFBP1	1.248			RAC1	1.260		
BMP1	1.243			STEAP1	1.254		
CTNNB1	1.243			MSN	1.248		
STAT3	1.214			STAT3	1.248		
AKT1	1.209			ITGB1	1.243		
F11R	1.209			ERBB3	1.228		
ERBB3	1.203			MMP9	1.223		
VPS13A	1.195			JAG1	1.200		
KRT14	1.187			AKT1	1.181		
SIP1	1.181			VPS13A	1.176		
TIMP1	1.178			NOTCH1	1.159		
PLEK2	1.176			DSP	1.157		
ITGA5	1.170			TCF3	1.151		
GSK3B	1.143			SNAI2	1.149		
TMEFF1	1.143			MMP2	1.146		
ITGB1	1.141			VIM	1.141		
SPP1	1.123			TFPI2	1.133		
TCF3	1.084			BMP2	1.128		
SMAD2	1.057			FN1	1.123		
CDH2	1.050			DSC2	1.117		
PTP4A1	1.050			WNT5B	1.110		
ESR1	1.043			NODAL	1.087		
FZD7	1.033			TMEM132	1.077		
ZEB1	1.028			CAV2	1.072		
SNAI1	1.026			CAMK2N1	1.062		
DSC2	1.009			TGFB1	1.045		
RAC1	1.005			SPARC	1.014		
JAG1	-1.019			PLEK2	1.007		
DSP	-1.069			CDH1	1.002		
TGFB1	-1.077			TMEFF1	-1.005		
SERPINE1	-1.102			SNAI1	-1.074		
FN1	-1.125			FZD7	-1.130		
CAMK2N1	-1.133			KRT7	-1.151		
NOTCH1	-1.178			WNT5A	-1.200		
ITGAV	-1.248			FOXC2	-1.376		
CAV2	-1.257			MMP3	-1.488		
TGFB3	-1.335			WNT11	-1.936		
SNAI2	-1.481			SERPINE1	-1.991		
EGFR	-2.047			AHNAK	-2.104		

Genes used for KEGG pathway enrichment analysis

Supplementary Table 5

CD36 vs TGFB signaling (KEGG)

Gene	Pearson's Correlation	P-value	FDR (BH)
CD36	1.000	0.002	0.013
BMP4	0.407	0.002	0.013
ROCK2	0.329	0.002	0.013
BMPR1B	-0.087	0.002	0.013
AMH	-0.143	0.002	0.013
COMP	-0.149	0.002	0.013
THBS2	-0.158	0.002	0.013
DCN	-0.165	0.002	0.013
BMP8B	-0.197	0.002	0.013
TGFB1	-0.200	0.002	0.013
INHBE	-0.203	0.002	0.013
BMP8A	-0.204	0.002	0.013
ID2	-0.205	0.002	0.013
LEFTY1	-0.134	0.004	0.023
SMAD5	-0.169	0.004	0.023
GDF5	0.386	0.006	0.030
SMAD3	0.203	0.006	0.030
TGFBR2	0.197	0.008	0.034
ID1	-0.135	0.008	0.034
SKP1	-0.166	0.008	0.034
TGFB3	-0.096	0.012	0.049
NODAL	0.185	0.014	0.052
INHBB	-0.115	0.014	0.052
CUL1	0.168	0.022	0.079
CREBBP	0.150	0.028	0.096
ZFYVE16	-0.143	0.030	0.099
E2F5	-0.115	0.036	0.114
RPS6KB2	-0.117	0.040	0.123
RBX1	-0.130	0.044	0.130
AMHR2	-0.074	0.052	0.149
MAPK1	0.127	0.054	0.150
PPP2CA	-0.131	0.058	0.156
ACVR1C	0.141	0.062	0.161
LEFTY2	-0.046	0.074	0.187
NOG	0.155	0.078	0.191
SMAD6	0.123	0.108	0.257
GDF6	-0.063	0.112	0.260
ZFYVE9	0.115	0.122	0.273
LTBP1	0.106	0.124	0.273
BMP2	-0.091	0.142	0.305
TGFB2	-0.063	0.146	0.306
PPP2R1A	0.095	0.150	0.307
SMURF1	-0.097	0.166	0.331
TNF	-0.084	0.172	0.336
THBS4	0.080	0.196	0.366
ACVR2A	-0.085	0.196	0.366
RBL2	0.087	0.206	0.376
SMAD1	0.081	0.234	0.418
THBS1	-0.072	0.277	0.487
PITX2	-0.060	0.283	0.488
ID4	-0.065	0.301	0.508
PPP2R1B	-0.068	0.329	0.545
SMAD4	0.056	0.411	0.667
RPS6KB1	0.049	0.419	0.668
BMP6	0.030	0.493	0.761
BMPR2	-0.047	0.509	0.761
ID3	-0.049	0.513	0.761
THBS3	-0.050	0.505	0.761
BMP5	0.015	0.557	0.812
SMAD7	0.031	0.593	0.828
RHOA	0.031	0.581	0.828
INHBA	-0.040	0.597	0.828
SMAD2	0.030	0.609	0.831
MYC	0.034	0.645	0.866
ACVR1	-0.036	0.663	0.866
RBL1	-0.037	0.665	0.866
SMURF2	0.021	0.721	0.885
ROCK1	0.020	0.711	0.885
ACVR2B	0.019	0.711	0.885
SP1	0.014	0.790	0.885
ACVRL1	0.009	0.774	0.885
FST	0.008	0.760	0.885
INHBC	-0.019	0.770	0.885
BMPR1A	-0.020	0.792	0.885
CHRD	-0.025	0.766	0.885
GDF7	-0.026	0.782	0.885
PPP2CB	-0.029	0.745	0.885
EP300	0.014	0.826	0.893
MAPK3	0.012	0.830	0.893
CDKN2B	-0.023	0.816	0.893
E2F4	0.000	0.894	0.938
TGFBR1	-0.007	0.888	0.938
IFNG	-0.007	0.928	0.962
BMP7	-0.012	0.980	0.984
TFDP1	-0.014	0.978	0.984
SMAD9	-0.014	0.984	0.984

CD36 vs WNT signaling (KEGG)

Gene	Pearson's Correlation	P-value	FDR (BH)
CD36	1.000	0.002	0.014
NKD1	0.568	0.002	0.014
AXIN2	0.474	0.002	0.014
CTNNB1	0.414	0.002	0.014
TCF7	0.384	0.002	0.014
DAAM1	0.360	0.002	0.014
ROCK2	0.329	0.002	0.014
PRKCA	0.303	0.002	0.014
SFRP5	-0.093	0.002	0.014
WNT9A	-0.105	0.002	0.014
PLCB4	-0.113	0.002	0.014
WNT2	-0.129	0.002	0.014
FZD1	-0.130	0.002	0.014
MMP7	-0.130	0.002	0.014
WNT7B	-0.139	0.002	0.014
WNT10A	-0.145	0.002	0.014
FZD7	-0.147	0.002	0.014
WNT4	-0.178	0.002	0.014
NKD2	-0.184	0.002	0.014
NFAT5	0.313	0.004	0.021
PPP3R1	0.262	0.004	0.021
CSNK1A1	0.182	0.004	0.021
WNT10B	-0.129	0.004	0.021
DKK2	-0.138	0.004	0.021
TCF7L1	-0.155	0.004	0.021
CTBP2	-0.162	0.004	0.021
PRKX	-0.168	0.004	0.021
BTRC	0.219	0.006	0.026
NFATC3	0.205	0.006	0.026
SMAD3	0.203	0.006	0.026
CER1	-0.053	0.006	0.026
FRAT1	-0.167	0.006	0.026
FRAT2	-0.178	0.006	0.026
LEF1	0.272	0.008	0.030
FZD4	0.247	0.008	0.030
PRKACB	0.195	0.008	0.030
PPP2R5A	-0.165	0.008	0.030
SKP1	-0.166	0.008	0.030
WNT2B	0.341	0.010	0.034
DAAM2	0.273	0.010	0.034
PRICKLE2	0.239	0.010	0.034
PPP2R5C	0.196	0.010	0.034
CCND2	0.413	0.012	0.038
NFATC4	0.350	0.012	0.038
SFRP4	0.307	0.012	0.038
CTNNBIP1	0.187	0.014	0.044
WIF1	0.300	0.020	0.060
VANGL2	-0.111	0.020	0.060
CUL1	0.168	0.022	0.065
WNT5B	-0.122	0.024	0.070
AXIN1	-0.140	0.026	0.074
CREBBP	0.150	0.028	0.078
DKK1	0.226	0.036	0.099
RBX1	-0.130	0.044	0.118
CAMK2B	0.184	0.048	0.121
CCND3	0.155	0.048	0.121
MAP3K7	0.150	0.048	0.121
SFRP2	-0.078	0.046	0.121
CSNK2A1	-0.121	0.056	0.138
PPP2CA	-0.131	0.058	0.141
RAC2	-0.099	0.070	0.167
TBL1X	0.140	0.078	0.184
MAPK8	0.123	0.082	0.187
PPP2R5B	0.115	0.082	0.187
SENP2	0.129	0.084	0.189
CHP1	-0.107	0.086	0.191
DVL1	0.132	0.088	0.192
DKK4	0.117	0.112	0.241
MAPK10	0.114	0.120	0.255
WNT16	0.100	0.122	0.255
CSNK2A2	0.105	0.134	0.277
FZD5	-0.096	0.144	0.293
PPP2R1A	0.095	0.150	0.301
FOSL1	0.087	0.152	0.301
FBXW11	-0.098	0.154	0.301
TCF7L2	0.096	0.160	0.309
PPARD	-0.084	0.170	0.324
DVL3	0.082	0.202	0.381
JUN	0.090	0.208	0.385
FZD9	0.080	0.212	0.385
WNT3A	0.075	0.212	0.385
APC2	-0.063	0.234	0.420
FZD8	-0.077	0.244	0.433
PPP2R5D	0.083	0.265	0.456
PLCB2	-0.071	0.263	0.456
MAPK9	-0.080	0.259	0.456
GSK3B	0.063	0.319	0.542
VANGL1	0.055	0.329	0.544
CACYBP	-0.066	0.331	0.544
PPP2R1B	-0.068	0.329	0.544
LRP6	0.062	0.335	0.544
CAMK2D	0.068	0.365	0.581
TP53	-0.062	0.363	0.581
DVL2	0.061	0.379	0.590
CHP2	-0.047	0.377	0.590
SMAD4	0.056	0.411	0.615
SFRP1	0.034	0.399	0.615
PSEN1	-0.053	0.403	0.615
NLK	-0.059	0.411	0.615
CSNK1A1L	0.048	0.421	0.623
RAC3	0.049	0.435	0.638
PRKCB	0.031	0.449	0.652
FZD2	-0.047	0.461	0.660
PLCB1	-0.057	0.463	0.660
PPP3CA	0.044	0.473	0.668
FZD10	0.025	0.483	0.669
PRKACA	-0.051	0.479	0.669
CHD8	-0.049	0.489	0.671
NFATC2	-0.048	0.499	0.679
CAMK2A	0.022	0.517	0.697
TBL1XR1	0.038	0.527	0.703
APC	-0.048	0.531	0.703
FZD3	-0.042	0.559	0.734
RHOA	0.031	0.581	0.748
PPP3R2	0.019	0.577	0.748
RUVBL1	-0.041	0.585	0.748
SMAD2	0.030	0.609	0.755
WNT8A	0.020	0.615	0.755
WNT7A	0.001	0.609	0.755
WNT11	-0.038	0.615	0.755
NFATC1	-0.039	0.599	0.755
PRKACG	-0.036	0.635	0.767
SOX17	-0.036	0.633	0.767
MYC	0.034	0.645	0.771
RAC1	-0.034	0.649	0.771
PPP3CC	0.007	0.695	0.819
ROCK1	0.020	0.711	0.832
PRICKLE1	-0.033	0.717	0.832
CCND1	0.014	0.731	0.842
PPP2CB	-0.029	0.745	0.852
WNT9B	-0.005	0.776	0.882
LRP5	0.008	0.802	0.893
PORCN	-0.020	0.804	0.893
PRKCG	-0.029	0.796	0.893
FZD6	-0.021	0.812	0.895
EP300	0.014	0.826	0.902
CAMK2G	0.008	0.830	0.902
WNT5A	0.003	0.848	0.908
WNT8B	-0.002	0.848	0.908
CSNK2B	0.006	0.876	0.932
CXXC4	0.002	0.902	0.940
SIAH1	0.002	0.894	0.940
CTBP1	-0.010	0.896	0.940
PPP3CB	0.001	0.914	0.945
WNT3	-0.006	0.940	0.966
PLCB3	-0.008	0.954	0.967
WNT6	-0.020	0.950	0.967
PPP2R5E	-0.008	0.974	0.981
WNT1	-0.022	0.986	0.986

CD36 vs PPAR/TFs

Gene	Pearson's Correlation	P-value	FDR (BH)
CD36	1.000	0.002	0.005
PPARD	0.349	0.002	0.005
NFKB1	0.233	0.002	0.005
NR1I2	-0.196	0.002	0.005
PPARA	0.296	0.006	0.013
HIF1A	0.209	0.014	0.026
FOS	0.190	0.032	0.050
NR1H2	0.065	0.341	0.469
PPARG	0.045	0.511	0.562
JUN	-0.058	0.499	0.562
STAT3	0.005	0.932	0.932

Supplementary Table 6

Low CD36 group						High CD36 group					
Gene Set Name	ES	NES	NOM p-val	FDR q-val	FWER p-val	Gene Set Name	ES	NES	NOM p-val	FDR q-val	FWER p-val
LIN_APC_TARGETS	0.481	1.873	0.000	1.000	0.604	WAKABAYASHI_ADIPOGENESIS_PPARG_RXRA_BOUND_WITH_H4K20ME1_MARK	-0.466	-2.100	0.000	0.099	0.113
LANDIS_BREAST_CANCER_PROGRESSION_UP	0.606	1.866	0.000	1.000	0.632	RUAN_RESPONSE_TO_TROGLITAZONE_UP	-0.660	-1.940	0.000	0.276	0.386
WAMUNYOKOLI_OVARIAN_CANCER_LMP_UP	0.454	1.856	0.002	1.000	0.656	GINESTIER_BREAST_CANCER_ZNF217_AMPLIFIED_UP	-0.593	-1.914	0.000	0.237	0.466
AMIT_EGF_RESPONSE_480_MCF10A	0.631	1.838	0.000	1.000	0.701	CHIANG_LIVER_CANCER_SUBCLASS_CTNNB1_UP	-0.850	-1.903	0.000	0.201	0.500
CREIGHTON_AKT1_SIGNALING_VIA_MTOR_UP	0.565	1.834	0.006	0.943	0.711	CHIANG_LIVER_CANCER_SUBCLASS_INTERFERON_DN	-0.789	-1.874	0.000	0.217	0.582
CHEN_HOXA5_TARGETS_9HR_DN	0.570	1.789	0.000	1.000	0.815	KANG_DOXORUBICIN_RESISTANCE_DN	-0.651	-1.842	0.006	0.250	0.680
BOYAULT_LIVER_CANCER_SUBCLASS_G1_UP	0.586	1.778	0.012	1.000	0.837	SUZUKI_RESPONSE_TO_TSA	-0.605	-1.824	0.004	0.256	0.724
JEPSEN_SMRT_TARGETS	0.566	1.775	0.002	1.000	0.847	SCHAEFFER_PROSTATE_DEVELOPMENT_AND_CANCER_BOX1_UP	-0.584	-1.819	0.014	0.237	0.739
LOCKWOOD_AMPLIFIED_IN_LUNG_CANCER	0.311	1.758	0.015	1.000	0.884	BOYAULT_LIVER_CANCER_SUBCLASS_G1_DN	-0.747	-1.819	0.006	0.211	0.739
COULOUARN_TEMPORAL_TGFB1_SIGNATURE_UP	0.500	1.755	0.004	1.000	0.889	MOOTHA_GLYCOGEN_METABOLISM	-0.630	-1.817	0.002	0.192	0.741
RAMASWAMY_METASTASIS_UP	0.461	1.753	0.008	0.983	0.892	WANG_CLASSIC_ADIPOGENIC_TARGETS_OF_PPARG	-0.725	-1.813	0.002	0.181	0.751
LI_AMPLIFIED_IN_LUNG_CANCER	0.484	1.748	0.008	0.950	0.899	GERHOLD_ADIPOGENESIS_UP	-0.549	-1.793	0.006	0.204	0.800
DARWICHE_SKIN_TUMOR_PROMOTER_UP	0.469	1.747	0.000	0.883	0.903	BOYAULT_LIVER_CANCER_SUBCLASS_G6_UP	-0.748	-1.782	0.012	0.213	0.834
TIMOFEEVA_GROWTH_STRESS_VIA_STAT1_DN	0.666	1.738	0.006	0.901	0.913	HOSHIDA_LIVER_CANCER_SURVIVAL_DN	-0.571	-1.775	0.027	0.210	0.846
LANDIS_ERBB2_BREAST_TUMORS_324_UP	0.469	1.737	0.002	0.851	0.914	RUAN_RESPONSE_TO_TNF_DN	-0.521	-1.744	0.008	0.266	0.891
ZEMBUTSU_SENSITIVITY_TO_NIMUSTINE	0.670	1.735	0.000	0.810	0.914	YANG_MUC2_TARGETS_DUODENUM_3MO_DN	-0.757	-1.740	0.012	0.260	0.900
CHARAFE_BREAST_CANCER_LUMINAL_VS_MESENCHYMAL_UP	0.489	1.731	0.014	0.795	0.920	LUCAS_HNF4A_TARGETS_UP	-0.534	-1.739	0.027	0.248	0.901
DACOSTA_UV_RESPONSE_VIA_ERCC3_TTD_UP	0.425	1.720	0.014	0.835	0.934	STEGER_ADIPOGENESIS_UP	-0.705	-1.737	0.008	0.238	0.903
BOYAULT_LIVER_CANCER_SUBCLASS_G2	0.651	1.718	0.007	0.808	0.935	CHEOK_RESPONSE_TO_HD_MTX_DN	-0.489	-1.724	0.010	0.254	0.920
DASU_IL6_SIGNALING_UP	0.614	1.709	0.004	0.833	0.940	IIZUKA_LIVER_CANCER_PROGRESSION_G2_G3_UP	-0.729	-1.723	0.028	0.243	0.921
WEIGEL_OXIDATIVE_STRESS_BY_TBH_AND_H2O2	0.545	1.709	0.004	0.795	0.940	BURTON_ADIPOGENESIS_10	-0.481	-1.722	0.021	0.234	0.921
GRAESSMANN_RESPONSE_TO_MC_AND_SERUM_DEPRIVATION_DN	0.426	1.701	0.002	0.822	0.955	RODWELL_AGING_KIDNEY_DN	-0.473	-1.720	0.006	0.227	0.924
GINESTIER_BREAST_CANCER_ZNF217_AMPLIFIED_DN	0.375	1.692	0.035	0.859	0.960	LEE_LIVER_CANCER_SURVIVAL_UP	-0.734	-1.717	0.017	0.223	0.927
POMEROY_MEDULLOBLASTOMA_DESMOPLASIC_VS_CLASSIC_DN	0.581	1.690	0.012	0.838	0.960	WOO_LIVER_CANCER_RECURRENCE_DN	-0.822	-1.714	0.008	0.220	0.933
WOO_LIVER_CANCER_RECURRENCE_UP	0.737	1.689	0.000	0.815	0.961	GUO_TARGETS_OF_IRS1_AND_IRS2	-0.522	-1.711	0.037	0.216	0.937
CHIARETTI_ACUTE_LYMPHOBLASTIC_LEUKEMIA_ZAP70	0.524	1.687	0.012	0.792	0.961	GUILLAUMOND_KLF10_TARGETS_DN	-0.644	-1.707	0.019	0.217	0.942
KAPOSI_LIVER_CANCER_MET_UP	0.638	1.685	0.006	0.780	0.964	LINDGREN_BLADDER_CANCER_WITH_LOH_IN_CHR9Q	-0.406	-1.703	0.058	0.216	0.946
CHARAFE_BREAST_CANCER_BASAL_VS_MESENCHYMAL_UP	0.613	1.685	0.010	0.754	0.964	SHEN_SMARCA2_TARGETS_UP	-0.410	-1.703	0.051	0.209	0.946
RICKMAN_METASTASIS_DN	0.466	1.682	0.021	0.746	0.965	BOYAULT_LIVER_CANCER_SUBCLASS_G123_DN	-0.846	-1.703	0.008	0.202	0.946
COLDREN_GEFITINIB_RESISTANCE_DN	0.597	1.680	0.016	0.733	0.966	KIM_GERMINAL_CENTER_T_HELPER_DN	-0.582	-1.696	0.016	0.207	0.955
SMID_BREAST_CANCER_RELAPSE_IN_LUNG_UP	0.745	1.674	0.006	0.751	0.973	FRASOR_RESPONSE_TO_SERM_OR_FULVESTRANT_UP	-0.579	-1.677	0.008	0.238	0.963
MEISSNER_BRAIN_HCP_WITH_H3K27ME3	0.597	1.668	0.002	0.769	0.975	LEE_LIVER_CANCER_DENA_DN	-0.663	-1.675	0.006	0.233	0.964
FERNANDEZ_BOUND_BY_MYC	0.340	1.665	0.008	0.766	0.976	BURTON_ADIPOGENESIS_6	-0.437	-1.675	0.039	0.227	0.964
LU_TUMOR_ANGIOGENESIS_UP	0.716	1.664	0.004	0.747	0.977	LANDIS_ERBB2_BREAST_PRENEOPLASTIC_DN	-0.477	-1.659	0.024	0.254	0.973
TOMLINS_METASTASIS_DN	0.569	1.664	0.019	0.731	0.977	MOOTHA_FFA_OXYDATION	-0.672	-1.655	0.078	0.257	0.973
JOHANSSON_GLIOMAGENESIS_BY_PDGFB_DN	0.703	1.660	0.010	0.737	0.981	LEE_LIVER_CANCER_CIPROFIBRATE_DN	-0.629	-1.649	0.012	0.263	0.977
TOMIDA_METASTASIS_UP	0.574	1.660	0.019	0.719	0.981	SOTIRIOU_BREAST_CANCER_GRADE_1_VS_3_DN	-0.520	-1.638	0.030	0.282	0.983
BENPORATH_ES_CORE_NINE_CORRELATED	0.513	1.657	0.014	0.716	0.983	WANG_RESPONSE_TO_ANDROGEN_UP	-0.474	-1.635	0.034	0.282	0.984
ZUCCHI_METASTASIS_DN	0.591	1.656	0.029	0.705	0.984	CHIANG_LIVER_CANCER_SUBCLASS_PROLIFERATION_DN	-0.836	-1.634	0.015	0.277	0.985
NIELSEN_SYNOVIAL_SARCOMA_UP	0.737	1.655	0.008	0.697	0.986	NADLER_OBESITY_DN	-0.456	-1.618	0.030	0.310	0.990
PATTERSON_DOCETAXEL_RESISTANCE	0.592	1.653	0.010	0.691	0.986	LEE_LIVER_CANCER_E2F1_DN	-0.604	-1.609	0.040	0.325	0.993
SHETH_LIVER_CANCER_VS_TXNIP_LOSS_PAM2	0.482	1.652	0.008	0.680	0.986	CHANGOLKAR_H2AFY_TARGETS_DN	-0.502	-1.608	0.021	0.319	0.994
TURASHVILI_BREAST_DUCTAL_CARCINOMA_VS_DUCTAL_NORMAL_UP	0.706	1.648	0.010	0.688	0.986	LEE_LIVER_CANCER_MYC_TGFA_DN	-0.656	-1.608	0.032	0.313	0.994
HUPER_BREAST_BASAL_VS_LUMINAL_DN	0.591	1.648	0.012	0.676	0.987	WAKABAYASHI_ADIPOGENESIS_PPARG_RXRA_BOUND_36HR	-0.328	-1.606	0.019	0.311	0.994
LEI_MYB_TARGETS	0.434	1.646	0.012	0.668	0.987	DING_LUNG_CANCER_EXPRESSION_BY_COPY_NUMBER	-0.400	-1.605	0.078	0.307	0.995
PIONTEK_PKD1_TARGETS_DN	0.675	1.645	0.008	0.663	0.988	IIZUKA_LIVER_CANCER_PROGRESSION_L1_G1_UP	-0.698	-1.603	0.033	0.305	0.995
MEISSNER_NPC_HCP_WITH_H3K27ME3	0.631	1.643	0.002	0.659	0.989	YANG_MUC2_TARGETS_DUODENUM_6MO_DN	-0.661	-1.601	0.015	0.304	0.995
KORKOLA_TERATOMA	0.643	1.642	0.010	0.651	0.990	BOYAULT_LIVER_CANCER_SUBCLASS_G12_DN	-0.851	-1.600	0.004	0.299	0.995
HAMAI_APOPTOSIS_VIA_TRAIL_DN	0.520	1.641	0.006	0.644	0.990	LEE_LIVER_CANCER_MYC_E2F1_DN	-0.655	-1.600	0.024	0.294	0.995
KANG_FLUOROURACIL_RESISTANCE_DN	0.703	1.638	0.008	0.649	0.990	NAKAYAMA_SOFT_TISSUE_TUMORS_PCA2_DN	-0.636	-1.592	0.026	0.306	0.995

Supplementary Table 7

Low BMI group						High BMI group					
**GENE SET**	**ES**	**NES**	**NOM p-val**	**FDR q-val**	**FWER p-val**	**GENE SET**	**ES**	**NES**	**NOM p-val**	**FDR q-val**	**FWER p-val**
YAMASHITA_LIVER_CANCER_WITH_EPCAM_UP	0.597	1.910	0.008	1.000	0.478	BARRIER_COLON_CANCER_RECURRENCE_UP	-0.530	-2.041	0.002	0.581	0.197
ZHAN_VARIABLE_EARLY_DIFFERENTIATION_GENES_DN	0.501	1.908	0.002	0.521	0.481	BYSTRYKH_HEMATOPOIESIS_STEM_CELL_AND_BRAIN_QTL_CIS	-0.382	-1.968	0.002	0.628	0.346
OZEN_MIR125B1_TARGETS	0.552	1.868	0.006	0.525	0.601	WENG_POR_TARGETS_LIVER_UP	-0.714	-1.952	0.000	0.480	0.385
NIKOLSKY_BREAST_CANCER_7P22_AMPLICON	0.645	1.862	0.006	0.416	0.621	ZAMORA_NOS2_TARGETS_DN	-0.439	-1.896	0.004	0.611	0.527
BOYAULT_LIVER_CANCER_SUBCLASS_G1_UP	0.614	1.820	0.002	0.508	0.733	WAKABAYASHI_ADIPOGENESIS_PPARG_RXRA_BOUND_WITH_H4K20ME	-0.413	-1.843	0.012	0.818	0.675
CHEN_HOXA5_TARGETS_9HR_DN	0.574	1.797	0.004	0.534	0.779	GUO_TARGETS_OF_IRS1_AND_IRS2	-0.568	-1.833	0.010	0.752	0.714
POMEROY_MEDULLOBLASTOMA_PROGNOSIS_DN	0.573	1.794	0.008	0.471	0.783	DITTMER_PTHLH_TARGETS_DN	-0.412	-1.825	0.010	0.701	0.741
WELCSH_BRCA1_TARGETS_DN	0.381	1.775	0.042	0.501	0.835	WARTERS_IR_RESPONSE_5GY	-0.585	-1.820	0.002	0.651	0.754
LEE_LIVER_CANCER_SURVIVAL_DN	0.549	1.765	0.008	0.488	0.853	HOEGERKORP_CD44_TARGETS_DIRECT_UP	-0.649	-1.820	0.002	0.579	0.754
MALONEY_RESPONSE_TO_17AAG_DN	0.440	1.764	0.034	0.446	0.856	HOSHIDA_LIVER_CANCER_SURVIVAL_DN	-0.602	-1.812	0.012	0.557	0.772
ZAMORA_NOS2_TARGETS_UP	0.446	1.724	0.018	0.594	0.905	GRADE_COLON_CANCER_DN	-0.560	-1.804	0.002	0.548	0.793
BOYAULT_LIVER_CANCER_SUBCLASS_G123_UP	0.663	1.717	0.006	0.581	0.914	CAIRO_LIVER_DEVELOPMENT_DN	-0.639	-1.800	0.008	0.525	0.805
SCHLOSSER_MYC_AND_SERUM_RESPONSE_SYNERGY	0.500	1.715	0.051	0.550	0.916	BRACHAT_RESPONSE_TO_CISPLATIN	-0.586	-1.800	0.014	0.485	0.805
CHAUHAN_RESPONSE_TO_METHOXYESTRADIOL_UP	0.513	1.712	0.017	0.526	0.920	KYNG_NORMAL_AGING_DN	-0.525	-1.799	0.002	0.452	0.809
RAMASWAMY_METASTASIS_UP	0.439	1.693	0.030	0.590	0.941	ACEVEDO_NORMAL_TISSUE_ADJACENT_TO_LIVER_TUMOR_UP	-0.407	-1.790	0.008	0.463	0.830
PETROVA_PROX1_TARGETS_UP	0.694	1.692	0.008	0.561	0.942	UEDA_PERIFERAL_CLOCK	-0.456	-1.784	0.017	0.461	0.847
PARK_HSC_AND_MULTIPOTENT_PROGENITORS	0.364	1.690	0.014	0.536	0.943	PIONTEK_PKD1_TARGETS_UP	-0.702	-1.772	0.004	0.488	0.868
AGUIRRE_PANCREATIC_CANCER_COPY_NUMBER_UP	0.306	1.674	0.028	0.583	0.956	GAZDA_DIAMOND_BLACKFAN_ANEMIA_ERYTHROID_UP	-0.550	-1.761	0.008	0.512	0.887
PEART_HDAC_PROLIFERATION_CLUSTER_UP	0.469	1.647	0.060	0.711	0.971	GROSS_HYPOXIA_VIA_ELK3_ONLY_UP	-0.630	-1.758	0.011	0.504	0.894
PRAMOONJAGO_SOX4_TARGETS_DN	0.472	1.643	0.020	0.698	0.974	HELLER_SILENCED_BY_METHYLATION_DN	-0.505	-1.753	0.002	0.507	0.900
MUELLER_PLURINET	0.439	1.633	0.056	0.723	0.981	FOURNIER_ACINAR_DEVELOPMENT_EARLY_UP	-0.627	-1.749	0.025	0.500	0.903
SANA_RESPONSE_TO_IFNG_DN	0.395	1.607	0.030	0.870	0.989	BOCHKIS_FOXA2_TARGETS	-0.435	-1.748	0.012	0.483	0.905
HSIAO_HOUSEKEEPING_GENES	0.329	1.606	0.144	0.837	0.989	PICCALUGA_ANGIOIMMUNOBLASTIC_LYMPHOMA_DN	-0.504	-1.748	0.024	0.463	0.906
NADERI_BREAST_CANCER_PROGNOSIS_UP	0.671	1.606	0.017	0.807	0.989	CHIANG_LIVER_CANCER_SUBCLASS_UNANNOTATED_UP	-0.710	-1.735	0.000	0.494	0.924
WONG_EMBRYONIC_STEM_CELL_CORE	0.456	1.604	0.089	0.786	0.990	HOWLIN_CITED1_TARGETS_2_DN	-0.640	-1.729	0.010	0.508	0.930
ZHANG_RESPONSE_TO_CANTHARIDIN_DN	0.399	1.603	0.082	0.763	0.990	HAHTOLA_MYCOSIS_FUNGOIDES_UP	-0.617	-1.721	0.008	0.527	0.942
COLLIS_PRKDC_SUBSTRATES	0.490	1.602	0.046	0.742	0.990	SERVITJA_LIVER_HNF1A_TARGETS_DN	-0.632	-1.718	0.000	0.521	0.946
LIN_APC_TARGETS	0.416	1.599	0.032	0.732	0.990	GERHOLD_ADIPOGENESIS_UP	-0.500	-1.715	0.018	0.517	0.948
BILANGES_SERUM_AND_RAPAMYCIN_SENSITIVE_GENES	0.541	1.599	0.091	0.710	0.990	LEE_LIVER_CANCER_CIPROFIBRATE_UP	-0.551	-1.713	0.002	0.510	0.950
BERTUCCI_INVASIVE_CARCINOMA_DUCTAL_VS_LOBULAR_UP	0.512	1.583	0.025	0.782	0.992	YAMASHITA_LIVER_CANCER_STEM_CELL_DN	-0.773	-1.703	0.002	0.540	0.956
FRASOR_RESPONSE_TO_SERM_OR_FULVESTRANT_DN	0.667	1.583	0.045	0.757	0.992	COULOUARN_TEMPORAL_TGFB1_SIGNATURE_DN	-0.500	-1.700	0.033	0.535	0.961
ZEMBUTSU_SENSITIVITY_TO_CYCLOPHOSPHAMIDE	0.601	1.581	0.032	0.741	0.992	VANLOO_SP3_TARGETS_DN	-0.494	-1.691	0.002	0.565	0.968
GRADE_COLON_AND_RECTAL_CANCER_UP	0.349	1.580	0.066	0.727	0.992	VANDESLUIS_COMMD1_TARGETS_GROUP_4_UP	-0.630	-1.686	0.002	0.576	0.975
NIKOLSKY_BREAST_CANCER_20Q12_Q13_AMPLICON	0.465	1.577	0.028	0.724	0.992	WENG_POR_DOSAGE	-0.715	-1.684	0.020	0.566	0.976
GARCIA_TARGETS_OF_FLI1_AND_DAX1_DN	0.447	1.571	0.053	0.736	0.992	DAUER_STAT3_TARGETS_UP	-0.613	-1.677	0.024	0.586	0.976
HEDENFALK_BREAST_CANCER_HEREDITARY_VS_SPORADIC	0.367	1.571	0.045	0.717	0.992	OHGUCHI_LIVER_HNF4A_TARGETS_DN	-0.716	-1.673	0.010	0.592	0.980
LIAO_HAVE_SOX4_BINDING_SITES	0.628	1.561	0.010	0.753	0.993	FLECHNER_PBL_KIDNEY_TRANSPLANT_OK_VS_DONOR_DN	-0.448	-1.669	0.036	0.595	0.981
OXFORD_RALA_OR_RALB_TARGETS_UP	0.678	1.557	0.054	0.755	0.993	ONDER_CDH1_TARGETS_1_UP	-0.446	-1.659	0.008	0.634	0.983
WU_APOPTOSIS_BY_CDKN1A_VIA_TP53	0.737	1.554	0.047	0.752	0.993	GROSS_HYPOXIA_VIA_ELK3_AND_HIF1A_UP	-0.469	-1.656	0.025	0.636	0.983
MODY_HIPPOCAMPUS_PRENATAL	0.481	1.548	0.081	0.764	0.994	HOSHIDA_LIVER_CANCER_SUBCLASS_S3	-0.677	-1.652	0.047	0.647	0.988
SONG_TARGETS_OF_IE86_CMV_PROTEIN	0.664	1.546	0.067	0.758	0.994	SMIRNOV_RESPONSE_TO_IR_6HR_UP	-0.399	-1.650	0.020	0.640	0.988
IRITANI_MAD1_TARGETS_DN	0.419	1.546	0.112	0.743	0.994	LUCAS_HNF4A_TARGETS_UP	-0.515	-1.644	0.045	0.655	0.991
BLUM_RESPONSE_TO_SALIRASIB_DN	0.479	1.545	0.081	0.730	0.995	LEE_LIVER_CANCER_MYC_TGFA_DN	-0.668	-1.643	0.018	0.647	0.992
NGO_MALIGNANT_GLIOMA_1P_LOH	0.491	1.544	0.073	0.718	0.995	GOLDRATH_IMMUNE_MEMORY	-0.491	-1.643	0.029	0.633	0.992
LE_EGR2_TARGETS_UP	0.611	1.544	0.061	0.705	0.995	YAMASHITA_LIVER_CANCER_WITH_EPCAM_DN	-0.845	-1.640	0.010	0.631	0.992
CROMER_METASTASIS_DN	0.449	1.540	0.055	0.707	0.995	BOYAULT_LIVER_CANCER_SUBCLASS_G1_DN	-0.688	-1.640	0.051	0.620	0.992
LU_EZH2_TARGETS_UP	0.395	1.539	0.062	0.698	0.995	PACHER_TARGETS_OF_IGF1_AND_IGF2_UP	-0.542	-1.639	0.004	0.608	0.992
KIM_ALL_DISORDERS_DURATION_CORR_DN	0.382	1.533	0.095	0.713	0.996	ZHENG_FOXP3_TARGETS_UP	-0.675	-1.638	0.019	0.600	0.992
LOCKWOOD_AMPLIFIED_IN_LUNG_CANCER	0.271	1.532	0.074	0.705	0.997	FALVELLA_SMOKERS_WITH_LUNG_CANCER	-0.487	-1.638	0.010	0.593	0.992
TOMIDA_METASTASIS_UP	0.532	1.529	0.046	0.705	0.997	LIANG_HEMATOPOIESIS_STEM_CELL_NUMBER_SMALL_VS_HUGE_UP	-0.473	-1.638	0.014	0.582	0.992

Figure 1: Fatty acid uptake and EMT markers in TCGA liver cancer dataset. A. 158 samples from the TCGA LIHC database were analyzed to determine the effect of patient's BMI on EMT and FA uptake gene expression. Box plot showing comparison of EMT scores, mRNA expression levels of hepatic FA uptake genes and *CD36* in groups of patients with BMI < 25 (Low BMI, n=75) or ≥ 25 (High BMI, n=83) P-values indicate significance levels from two-tailed Student's T-test. B. Scatter plots showing correlation between BMI (X-axis) and EMT score, *CD36*, *ZEB1* or *FN1* (Y-axis). The solid red line indicates linear fit, with 95% confidence intervals indicated by dotted black lines. r indicates Pearson's correlation coefficient and p-values indicate significance of correlation (two-tailed), with the red boxes marking significant associations from the multivariate regression analysis. C. Heatmap showing relative expression levels of hepatic fatty acid uptake genes and EMT genes in the TCGA liver cancer dataset. Gene clusters (vertical axis) were obtained by hierarchical clustering and samples (horizontal axis) were ordered according to the EMT score. D. Scatter plot showing association between the fatty acid uptake genes (Y-axis) and EMT score (X-axis) drawn from 158 patient samples in the TCGA dataset. The solid red line indicates linear fit, with 95% confidence intervals indicated by dotted black lines. r indicates Pearson's correlation coefficient and p-values indicate significance of correlation (two-tailed), with the red boxes marking significant associations from the multivariate regression analysis (n=158). Clinical data for each patient is available in Supplementary Table 1 (Bonferroni adjusted p-value cut-off for the analysis = 0.0016).

Figure 2: CD36 and EMT marker expression in human HCC tumors. A. Western blots showing expression levels of CD36 and various EMT markers in tumor samples grouped according to BMI. High BMI group (n=8) represents tumor samples obtained from individuals with BMI ≥ 30 and low BMI group (n=8) indicates individuals with BMI ≤ 25. Equal amounts of sample (40μg) were loaded in each well, run under identical conditions and blotted with specific primary antibodies against the indicated proteins. For clarity, blots have been cropped to show relevant bands (full length blots are presented in Supplementary Figure 3) B. Expression levels of proteins were determined by quantifying the

background-subtracted band intensities normalized to GAPDH. Each dot represents relative expression levels of individual samples, with the horizontal bar indicating the median of the distribution. P-values indicate significance of difference in means between low BMI and high BMI groups determined by Mann-Whitney U test, with red boxes marking significant associations from multivariate regression analysis. C. Scatter plot showing association between CD36 (X-axis) and EMT marker (Y-axis) expression. The solid lines indicate linear fit and the dotted lines indicate 95% confidence intervals. r indicates Pearson's correlation coefficient and p-values indicate significance of correlation (two-tailed), with red boxes marking significant associations from multivariate regression analysis (n=16). Specific clinical data for each patient is available in Supplementary Table 3.

Figure 3: PA mediated EMT induction. A. Scratch-wound cell migration assays with HepG2 and Hep3B cells treated with different concentrations of PA (0.1mM to 0.4mM) and BSA (control). The plot indicates average number of migrating cells in the wound area at 24 and 48 hours following 5-day treatment normalized to 0 hour time point (n=3). P-values indicate the significance levels compared to BSA at a given time point determined by Tukey's test following one-way ANOVA. B. Phase contrast microscopy images of HepG2 and Hep3B cells treated with BSA or PA (0.3mM) at 48 hours (40X magnification, scale bar=50µm). C. Confocal images of HepG2 demonstrating changes in morphology upon PA treatment. Keratin 18 and nuclear staining are indicated by green and blue fluorescence respectively. (40X magnification, scale bar=100µm). D. Modified Boyden's chamber assays with HepG2 and Hep3B cells. The bar graph indicates the average number of migrating or invading cells (±SEM, Standard Error of the Mean) counted in 5 fields of view per insert (n=3). P-values indicate significance levels determined by Student's t-test (two-tailed). E. Bright field images at 5X (scale bar=400µm) magnification showing fragmentation in monolayers of BSA or PA treated Hep3B or HepG2 cells following dispase treatment and application of mechanical stress. F. Particle analysis following dispase assay. Bar graphs showing average particle count and fragment diameter (±SEM) determined from 5

independent images panels obtained after dispase treatment and applying mechanical stress to BSA or PA treated HepG2 and Hep3B cells. P-values indicate significance levels determined by Student's t-test.

Figure 4: Cytotoxicity vs. metabolic activity. A. Bar graph showing average cytotoxicity (±SEM) determined using LDH assay in HepG2 or Hep3B cells following 5 day (with serum) and 2 day (without serum) treatment with BSA or PA at different concentrations (n=3). P-values indicate significance levels comparing average LDH release in PA treated cells with BSA treated cells determined by Student's t-test (two tailed). B. Bar graph showing the relative metabolic activity represented by average fluorescence (±SEM) from reduced AlamarBlue reagent (resazurin) in HepG2 or Hep3B cells following 5 day treatment (with serum) and 2 day (without serum) with BSA or PA (n=3). P-values indicate significance levels determined by Student's t-test (two tailed). Numbers above bars indicate the percentage of proliferating cells at 48 hours compared to 0 hours. C. Neutral lipids and fatty acids in HepG2 or Hep3B cells were stained with Oil Red O following 5 day (with serum) and 2 day (without serum) treatment with BSA or PA. Bright-field microscopy images were recorded at 40X magnification (scale bar=50µm). D. Relative levels of lipids and fatty acids in BSA and PA treated cells were quantified by eluting the Oil Red O dye and measuring relative absorbance at 500nm. Bar graph shows average absorbance (±SEM) in BSA and PA treated cells. P-values indicate significance levels comparing average absorbance in BSA or PA treated cells determined by Student's t-test (two tailed).

Figure 5: Population effect of PA on EMT marker expression. A. Density plots showing distribution of cells co-stained with zombie violet (Y-axis) and either CDH, DSP or VIM (X-axis) in HepG2 cells treated with BSA or PA (0.3mM). The bars represent staining thresholds for zombie violet (horizontal bar) or the protein markers (vertical bar). Lower left quadrant represents background staining, upper left quadrant represents cells stained with only zombie violet, upper right quadrant represent cells co-stained with zombie violet and protein marker and lower right quadrant represent cells stained with protein marker only. The numbers in the plot represent the percentage of total cells within that quadrant. B. Confocal images showing specific regions with altered expression levels of CDH and VIM in HepG2 and

Hep3B cells. Blue indicates nuclear staining and green indicates CDH or VIM staining. Images were recorded at 40X with identical exposure and PMT settings maintained between BSA and PA treated cells for a given marker (scale bar = 100μm). C Bar graph showing fold-change in mRNA expression levels of various EMT transcription factors determined by qRT-PCR in PA (0.3mM) treated HepG2 cells compared to corresponding expression levels in BSA treated cells (*GAPDH* normalized). P-values indicate significance levels compared to BSA determined by Student's t-test (two tailed), n = 3.

Figure 6: EMT pathways induced by PA. A. Heatmap showing relative fold-change in mRNA expression levels of various EMT-related genes in PA (0.3mM) vs. BSA treated cells measured by EMT-array. The enlarged heatmap shows fold change in expression levels of the components of TGFB- and Wnt-signaling pathways included in the array. B. The top 20 genes ranked according by fold-change in the array were enriched for KEGG pathways. The table shows the term (KEGG pathway) enriched, number and percentage of genes in the input set belonging to the pathway and significance levels of enrichment. C-F. Confocal images showing expression of β-catenin or SMAD2/3 (green) and nuclei (blue) in HepG2 and Hep3B cells treated with BSA or PA (0.3mM). The overlap panel shows pixels where both green and blue fluorescence co-localize, therefore indicating cells displaying translocation of β-catenin or SMAD2/3 transcription factors to the nucleus. Images were recorded at 40X with identical exposure and PMT settings maintained between BSA and PA treated cells for a given marker (scale bar = 100μm).

Figure 7: Correlation matrix heatmaps showing the association between mRNA expression z-scores (TCGA) of *CD36* and A. Wnt signaling pathway genes (KEGG) B. TGF beta signaling pathway (KEGG) C. PPARs and transcriptional mediators of inflammatory pathways known to be induced by free fatty acids. Genes are clustered with average linkage hierarchical clustering using 1-Pearson's correlation as distance metric. Black boxes in A and B show the nearest neighbors of CD36 with strong positive correlation, with enlarged list showing the names of genes in that cluster. D. Overview of the influence of elevated free fatty acid uptake on cell signaling pathways resulting in the induction of EMT program. Red

ovals represent genes that were upregulated in vitro in PA treated HCC cells; orange ovals represent genes that show positive correlation with *CD36* in the TCGA dataset; white ovals represent genes that did not show a significant correlation with CD36 in the TCGA dataset. Dashed black arrows show transport/translocation, solid black arrows and bar-headed lines indicate positive and negative regulation respectively. Blue arrows indicate consequence of activation of the proteins in context of the EMT program.

Figure 8: CD36 and TGF-beta/Wnt-signaling mediate PA effects. A. Western blots showing expression of CDH and VIM in HepG2 cells treated with BSA or PA (0.3mM) for 5 days with serum. Additionally, the cells were co-treated with either 100µM SSO (CD36 inhibitor) or DMSO (solvent control). Equal amounts of sample (40µg) were loaded in each well, run under identical conditions and blotted with specific primary antibodies against the indicated proteins. For clarity, blots have been cropped to show relevant bands (full length blots are presented in Supplementary Figure 4). B-C. Bar graphs showing quantification of blots indicating expression levels of CDH and VIM normalized to GAPDH in BSA or PA treated cells (n=3). P-values indicate significance levels comparing SSO treated (+SSO) BSA or PA treated cells with DMSO control (-SSO) cells determined by Student's t-test (two tailed). D. HepG2 cells were treated with PA (0.3mM) and DMSO (control), β-catenin/Tcf inhibitor III (100nM) or TGF-β1 RI kinase inhibitor (50nM) in presence of serum for 5-days, followed by wound-healing assay in serum-free media over 48 hours. Bar graph indicates average wound closure (wound closure = 100 - % open wound area) normalized to 0 hour time point (n=3). P-value indicates significance levels comparing the open wound area in inhibitor treated cells with control cells determined by Student's t-test (two tailed). E. HepG2 cells were treated with PA (0.3mM) and DMSO (control), CD36 inhibitor SSO (100µM) or porcupine inhibitor LGK974 (100nM) in presence of serum for 5-days, followed by Boyden's chamber migration assay over 48 hours in serum-free media, with 10% FBS containing regular medium as chemoattractant. Bar graph indicates the average number of migrating cells (n=3). P-value

indicates significance levels comparing the number of migrating cells with inhibitor treatment against control, determined by Student's t-test (two tailed).

Supplementary Figure 1: Differential effects of FFAs on migration

Supplementary Figure 2: mRNA expression levels of Hedgehog ligand *SHH* and downstream transcription factor *GLI1*in PA vs. BSA treated HepG2 cells

Supplementary Figure 3: Uncropped western blot images supporting Figure 2A

Supplementary Figure 4: Uncropped western blot images supporting Figure 8A

Supplementary Figure 5: Flow cytometry gating and intensity plots

Supplementary Table 1: Log2 transformed TCGA LIHC data from cBioPortal for FA Uptake Genes: CD36, FABP1, FABP4, FABP5, SLC27A2, SLC27A5; Mesenchymal Genes: CDH2, FN1, SNAI1, SNAI2, TWIST1, TWIST2, VIM, ZEB1, ZEB2; Epithelial Genes: CDH1, CLDN4, CLDN7, MUC1, TJP3; EMT scores; and Patient clinical data: Age, gender, height, weight, BMI, EMT score and pathological (TNM) staging

Supplementary Table 2: Pearson's correlation coefficient and p-values (two-tailed) between EMT score and FA uptake genes, BMI and FA uptake genes, and BMI and EMT genes (Bonferroni adjusted p-value cut-off = 0.0016)

Supplementary Table 3: Clinical information including BMI, sex, grade and pathological staging information and results of western blot quantification for the corresponding samples

Supplementary Table 3: Palmitate, oleate and linoleic acid free fatty acid levels in HCC vs. normal cases

Supplementary Table 4: Fold-change in gene expression levels of EMT genes in PA vs. BSA treated HepG2 or Hep3B cells. Genes were ranked by fold-change and the highlighted genes (top-20) were used for KEGG pathway enrichment analysis. Direct transcriptional targets of TGF and WNT signaling pathways are marked.

Supplementary Table 5: Correlation analysis comparing expression profile of CD36 in TCGA HCC dataset with KEGG Wnt/TGFB signaling pathways genes, PPAR genes and the transcriptional effectors of inflammatory pathways known to be induced by free fatty acids.

Supplementary Table 6: Gene set enrichment analysis of TCGA HCC data with patients stratified into low CD36 and high CD36 expression clusters (only the top 50 gene sets are listed)

Supplementary Table 7: Gene set enrichment analysis of TCGA HCC data with patients stratified into low BMI and high BMI groups (only the top 50 gene sets are listed)
